# Rational Design of Organelle-Targeted Fluorescent Probes: Insights from Artificial Intelligence

**DOI:** 10.34133/research.0075

**Published:** 2023-03-08

**Authors:** Jie Dong, Jie Qian, Kunqian Yu, Shuai Huang, Xiang Cheng, Fei Chen, Hualiang Jiang, Wenbin Zeng

**Affiliations:** ^1^ Xiangya School of Pharmaceutical Sciences, Central South University, Changsha 410083, P.R. China.; ^2^ National Engineering Research Center of Rice and Byproduct Deep Processing, School of Food Science and Engineering, Central South University of Forestry and Technology, Changsha 410004, P.R. China.; ^3^ State Key Laboratory of Drug Research, Drug Discovery and Design Center, Shanghai Institute of Materia Medica, Chinese Academy of Sciences, Shanghai 201203, P.R. China.

## Abstract

Monitoring the physiological changes of organelles is essential for understanding the local biological information of cells and for improving the diagnosis and therapy of diseases. Currently, fluorescent probes are considered as the most powerful tools for imaging and have been widely applied in biomedical fields. However, the expected targeting effects of these probes are often inconsistent with the real experiments. The design of fluorescent probes mainly depends on the empirical knowledge of researchers, which was inhibited by limited chemical space and low efficiency. Herein, we proposed a novel multilevel framework for the prediction of organelle-targeted fluorescent probes by employing advanced artificial intelligence algorithms. In this way, not only the targeting mechanism could be interpreted beyond intuitions but also a quick evaluation method could be established for the rational design. Furthermore, the targeting and imaging powers of the optimized and synthesized probes based on this methodology were verified by quantitative calculation and experiments.

## Introduction

Subcellular organelles, as subunits of cells, play an indispensable role in different physiological processes. The imbalance of biologically active species in them will cause their dysfunction, which has a serious impact on the health of organisms [1,2]. Fluorescent probes have become a powerful tool for intracellular visualization by taking advantage of their high sensitivity, fast response time, strong specificity, and real-time imaging and are popular in bioimaging and biosensing [3–5]. In recent years, a large number of works based on the specificity of fluorescent probes for imaging different organelles have been reported [6,7], including specific imaging of mitochondria, Golgi apparatus, lysosome, and endoplasmic reticulum [8–12]. On this basis, some fluorescent probes for the imaging of enzymes, reactive oxygen species (ROS), and viscosity in these organelles have also been reported. The reported probes have achieved relatively good detection and imaging effects in the imaging of subcellular organelles and biologically active substances [13–17]. The targeting ability of these probes in actual imaging experiments, however, cannot reach the expectation, although there are specific targeting groups of different subcellular organelles that have been summarized according to current researches. In particular, there are some fluorescent molecules that, although coupled with specific targeting groups for subcellular organelles, still cannot be specific [18,19]. One of the major causes is that most of these probes were designed mainly depending on empirical knowledge, which was limited by small chemical space and low efficiency. Therefore, how to solve the problem of discrepancies between experimental results and design, so that researchers can reasonably and accurately design specific subcellular organelle probes before the construction of probes, is a great challenge.

In recent years, with the explosive growth of biomedical data and the rapid development of computer software and hardware, artificial intelligence has increasingly penetrated deep into various aspects of drug research and development and has greatly boosted the process [20–22]. Especially in the field of drug design, prediction models based on artificial intelligence technology can be used to evaluate important properties in the early stage of drug development. For example, models established for predicting a series of important drug ADMET (absorption, distribution, metabolism, excretion, and toxicity) properties can effectively reduce the risk of failure due to poor pharmacokinetic properties in late-stage development, which can benefit from decreasing the cost and shortening the time [23–25]. In addition, artificial intelligence technology also embraces many methods for structure-based molecular transformation and optimization, which greatly expands the chemical space of drugs [26,27]. With these in mind, appropriate artificial intelligence models may have the potential to address the abovementioned challenges and provide a rapid prediction for organelle-targeted molecular probes, thus greatly reducing blindness.

Here, a new method integrated with advanced artificial intelligence with quantitative calculation was proposed to help provide new insights into the accurate design of specific subcellular organelle probes (as shown in the graphical abstract). Firstly, we collected the currently published fluorescent probes targeting subcellular organelles and chemical compounds with known subcellular localization. Then, we established a pretreated molecular library. On this basis, we developed a multilevel framework that consisted of a series of prediction models by employing advanced-artificial intelligence methods. The first level was a classification model (B-PvsC model) to uncover the reason why the subcellular organelle targeting of fluorescent probes were different from commonly seen chemical compounds. The second level was a binary classification model (B-MvsP model) expected to focus on mitochondrial targeting. The third level was a multiclassification model (M-PvsP model) expected to predict specific subcellular organelles targeting of a probe further. The last level was a colocalization model (B-McoL model) to estimate the targeting effect of a probe. Afterward, by taking advantage of the detailed explanations and summarized rules of these models, we designed several groups of probes on the basis of different fluorescence mechanisms and estimate them by our framework. Then, on this basis, we selected the fluorescent probes with better performance to synthesize them and verified their accuracy and biological application by experimental methods. In addition, we further combined the means of quantum chemistry to perform quantitative calculations on the synthesized molecules to explain their spectroscopic properties. With the above inspiring strategies, a method that enabled more accurate prediction of organelle-targeted fluorescent probes was successfully constructed. Thus, it would help researchers to design and construct novel organelle-targeted fluorescent probes more accurately and rationally, especially for mitochondria-targeted ones, and allow more effective detection of specific bioactive molecules, thereby advancing the field.

## Results

### Datasets

A total of 1,661 organelle-targeted fluorescent probes and 614 compounds that were not fluorescent probes (Np-Compounds) were manually collected from >10 thousand publications over the recent 10 years (2012–2022) (Table S1). These data items were used to construct datasets for the model building of our designed framework. All molecules were checked and converted into InChIKey for deduplication using ChemDes and PyBioMed [28,29]. Duplicate molecules within the same label were deleted, and duplicate molecules in different categories were retained. The dataset for building B-PvsC model to distinguish the mitochondria-targeted probes and commonly seen compounds consisted of 1,005 mitochondria-targeted fluorescent probes and 236 mitochondria-targeted Np-Compounds. After deduplication, that became 982 and 236, respectively. The dataset for B-MvsP model included 982 probes for mitochondrial targeting and 637 probes for other organelles. The dataset for M-PvsP model to predict specific subcellular organelle targeting of a probe consists of 41, 156, 370, 37, 1,005, and 52 fluorescent probes targeting the Golgi apparatus, endoplasmic reticulum, lysosome, cell membrane, mitochondria, and nucleus, respectively. After deduplication, that became 40, 153, 361, 36, 982, and 50, respectively.

In addition, for mitochondria-targeted fluorescent probes, we have also attempted to estimate the colocalization effect by predicting their correlation coefficients (B-McoL model). For the 1,005 mitochondrial targeting probes, their colocalization in the measurement system by different colocalization dyes was collected. The 914 data, for which the dyes were MitoTracker Green and MitoTracker Red (MTR), and numerical correlation coefficients were available, were selected and deduplicated according to InChIKey. Then, the arithmetic mean of multiple records for the same molecule was adopted. Since the colocalization data would only be reported when they obtained an expected probe with a good colocalization ability, the correlation coefficients were usually high. Specifically, the correlation coefficients of the retained 896 unique probes were centered overwhelmingly between 0.8 and 1. Therefore, we took 0.8 as the threshold and labeled 811 probes with correlation coefficients not <0.8 as the positive and 85 probes <0.8 as the negative. In addition, a synthetic minority oversampling technique [30] was adopted to oversample the minority class to compensate for the unbalanced sample distribution (811 positives versus 811 negatives; Fig. S1).

### The performance analysis of B-PvsC model

**Fig. 1.**
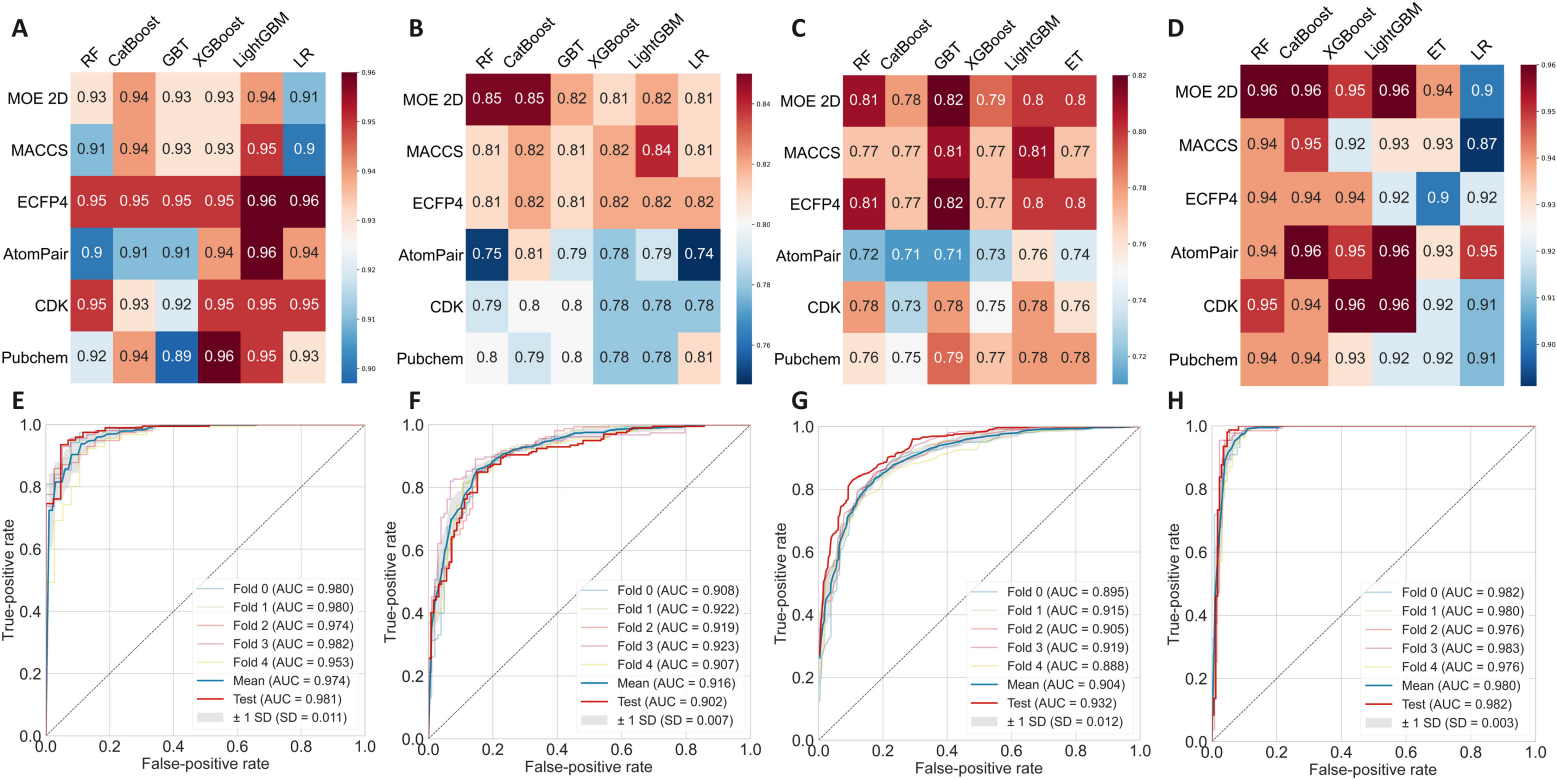
Results of machine learning models. The heatmaps displayed the accuracy for the test set of B-PvsC (A), B-MvsP (B), M-PvsP (C), and B-McoL (D) models constructed by different molecular representations and algorithms. The ROC curve plots for the CV and test set of the best B-PvsC (E), B-MvsP (F), M-PvsP (G), and B-McoL (H) models.

The optimal B-PvsC model for detecting mitochondria-targeted fluorescent probes and Np-Compounds was explored with a wide range of descriptors and algorithms. The best performance was achieved by ECFP4-LR model with an accuracy (ACC) of 0.93 and 0.95 for the cross-validation (CV) and test set, respectively (Table). The area under the curve (AUC) of 0.97 ± 0.01 (Mean ± SD) for CV showed excellent performance (Fig. 1E). In addition, 99.0% of probes and 79.07% of the Np-Compound in the test set were correctly predicted (Fig. S2). Among the 6 molecular representations, ECFP4 and Pubchem fingerprints achieved the highest accuracy on the test set, and the ECFP4-LR model was better during CV (Fig. 1A and Table S2).

**Table.** The best performance of the 4 models for the multilevel framework. The AUC, Recall, and Precision of the M-PvsP model were calculated with the weighted method.

Model	Feature	Algorithm	5-CV				Test			
			ACC	AUC	Recall	Precision	ACC	AUC	Recall	Precision
B-PvsC	ECFP4	LR	0.933	0.974	0.974	0.946	0.955	0.981	0.990	0.957
B-MvsP	MACCS	LightGBM	0.858	0.916	0.908	0.865	0.843	0.902	0.869	0.874
M-PvsP	MACCS	LightGBM	0.783	0.904	0.783	0.757	0.809	0.932	0.809	0.794
B-McoL	2D	LightGBM	0.941	0.980	0.961	0.927	0.963	0.982	0.981	0.944

### The performance analysis of B-MvsP model

The B-MvsP model tried to distinguish the probes targeting mitochondria and other organelles with mitochondria as positive and the other 5 organelles as negative. By comparing molecular descriptors/fingerprints and algorithms, the best model is the combination of MACCS fingerprints and LightGBM, with accuracy of 0.858 and 0.843 for the CV and test set, respectively (Table). The AUC of 0.916 ± 0.01 (Mean ± SD) for CV showed good performance (Fig. 1F). Among the 199 mitochondria-targeted probes in the test set, 86.93% of them (173 probes) were correctly predicted, and 80.00% of the 125 other probes were correctly predicted (Fig. S3). The 2D descriptors from Molecular Operating Environment (MOE), MACCS and ECFP4 fingerprints showed better performance in this prediction; all of them reported an accuracy of >0.8 in the test set. However, CDK fingerprints performed comparatively less well (Fig. 1B and Table S3).

### The performance analysis of M-PvsP model

The M-PvsP model was designed to further specify the more detailed localization of fluorescent probes among the 6 kinds of organelles. By comparing the predictive power of different kinds of descriptors and algorithms, the best model was the MACCS-LightGBM. The accuracy was 0.783 and 0.809 for the CV and test set, and the AUC was 0.904 and 0.932 (Table and Fig. 1G), respectively. In the test set, Golgi apparatus and nucleus probes, which have a smaller sample size, showed deficient prediction results in which most probes were incorrectly classified as mitochondria-targeted ones. While for the same smaller sample size of cell membrane-targeted probes, nearly 50% were correctly predicted. It can be noticed that because of the imbalance in sample size between the classes (up to 195:7 in the test set), 5 Golgi apparatus-targeted ones were misclassified as mitochondria (7 in total), while the endoplasmic reticulum, lysosome, and mitochondria all had high precision (70.00%, 80.52%, and 83.49%) in the MACCS-LightGBM model. For mitochondrial samples, it got a high recall rate of 93.33% (Fig. S4).

### The performance analysis of B-McoL model

The B-McoL model was developed to predict the colocalization ability of mitochondria-targeted fluorescent probes. A series of models were constructed by combining different algorithms and molecular descriptors. Among them, the best combination was the 2D-LightGBM model, with an accuracy of 0.941 for CV and 0.963 for the test set (Table). The AUC for CV was quite high reaching 0.98 (Fig. 1H). In addition to the optimized 2D molecular descriptors, CDK and AtomPair fingerprints also achieved comparable predictive performance, followed by MACCS fingerprints (Fig. 1D and Table S5). For the 154 probes of good colocalization ability, 98.05% were correctly predicted, and that was 94.74% for the 171 probes of weak colocalization ability. With a systematic oversampling of the inferior class, the error of assigning all data points to the dominant category due to unbalanced sample distribution was avoided. In addition, the decision boundaries of the LightGBM model were visualized via principal components analysis by compressing the information of the 2D descriptors into 2 principal components. Unlike fingerprints, the 2D molecular descriptors preserved considerable information after principal components analysis. Most of the data points fell into the regions of their right class, and the volume of information in the inferior class was not distorted by oversampling (Fig. S5).

### Model explanation and rules

**Fig. 2.**
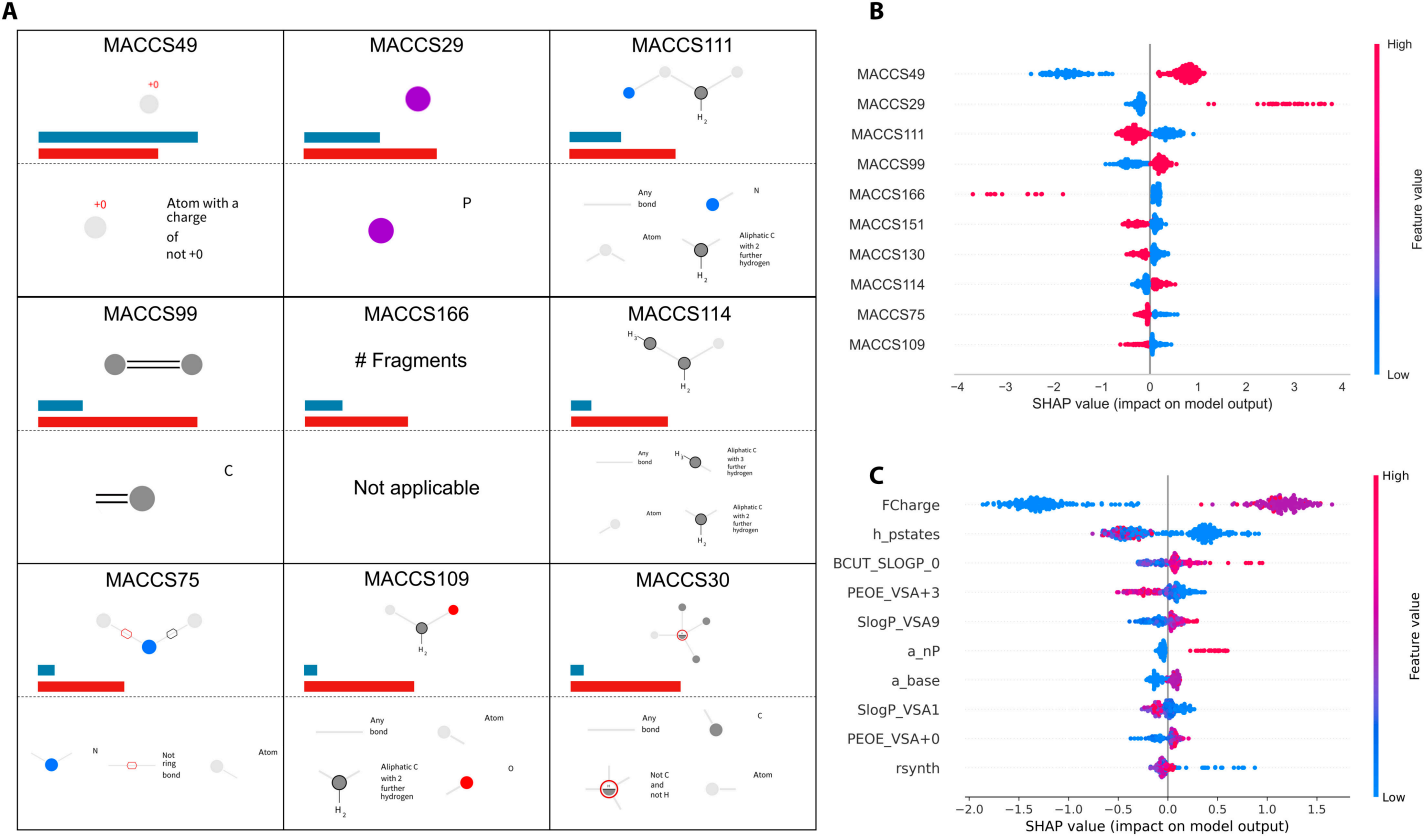
The explanation of the selected B-MvsP model. (A) The 9 most important MACCS keys that were codetermined in the Top15 of SHAP and feature importance methods driven by LightGBM algorithm. The bars represent the relative value of feature importance after Min-Max normalization (dark cyan, SHAP; claret, feature importance). The MACCS 166 is not applicable and represents whether a structure has disconnected fragments. (B) The SHAP values for the top 10 MACCS keys from the MACCS-LightGBM model. (C) The SHAP values for the top 10 MOE 2D descriptors from the 2D-LightGBM model.

To explore the relationships between molecular structures and the organelle-targeting effects, the model explanation was performed and rules were summarized. When focused on mitochondria-targeted ones, the B-MvsP model using MACCS fingerprints and LightGBM algorithm showed the best predictive power and good robustness. Therefore, we made an explanation for the model based on the interpretable SHAP (SHapley Additive exPlanations) values and the feature importance generated by LightGBM. Because of the differences between the selected algorithms, the feature importance calculated will not be identical, but the most important features will always have a higher score. Among the 166 MACCS keys, more than half of the Top50, Top30, and Top15 most important features selected preferentially via both methods overlapped, and 88% of the Top50 were mainly associated with heteroatoms such as nitrogen, oxygen, and phosphorus. A visualization of the 9 most important MACCS keys codetermined in the Top15 of SHAP and LightGBM methods is shown in Fig. 2A. Of these, MACCS166 cannot be visualized but represents whether a structure has disconnected fragments. This indicated that the charge was very important, as these fragments were usually anions and cations. MACCS49 and MACCS29, the 2 most important features identified by SHAP, represented charge and phosphorus elements, and both of them contributed positively to the mitochondrial targeting of probes (Fig. 2B). This was consistent with the conclusions of some published studies [31]. Moreover, the double carbon bonds (MACCS99) and ethyl groups (MACCS114) also had a facilitative effect on targeting mitochondria, while the presence of nitrogen-containing heterocycles and fragments had a negative effect (MACCS111 and MACCS75). Apparently, the double carbon bonds came from the conjugated structure that can produce fluorescence emission. Lipophilic groups were helpful for the probe to target mitochondria, while hydrophilic groups were the opposite. In addition to MACCS fingerprints, MOE 2D molecular descriptors covering lots of physicochemical and topological properties also had a good performance. Therefore, the 2D-LightGBM model was also interpreted to provide additional insights. The 10 most important descriptors determined by SHAP are shown in Fig. 2C. These descriptors described charge, molecular shape, surface area, energy, and synthetic feasibility. We speculated that probes with higher total formal charges, easily transformed conformations, and strong lipophilicity were more likely to target mitochondria. This could be confirmed and supplemented by the previous experimental analysis [31].

### Library design of mitochondria-targeted probes

To validate the practicality of the explanation and rules, and evaluate the predictive power of the framework, we first designed a series of fluorescent molecules based on excited-state intramolecular proton transfer (ESIPT), twisted intramolecular charge transfer (TICT), and ICT, which were 3 representative fluorescent mechanisms. Then, among these fluorescent molecules with different mechanisms, we further distinguished them into mitochondrial dyes and probes that could be specifically used for ROS, enzymes, and viscosity imaging in mitochondria according to their structural properties, which were 3 important biomedical applications of mitochondria-targeted probes. According to our plan, firstly, we designed 451 molecules of ICT and 70 molecules of ESIPT, respectively. Then, some of these molecules were connected with the corresponding ROS response sites [15,32] and alkaline phosphatase (ALP) sites [33,34] to further explore whether these molecules can still target mitochondria accurately and detect related biomarkers meanwhile. As a result, 37 ROS and 21 ALP probes were generated. In addition, we selected 54 molecules with TICT effect from ICT molecules for the imaging of intramitochondrial viscosity [35,36]. Finally, A library of 633 molecules designed above was set up, including 70, 451, 21, 37, and 54 molecules for ESIPT, ICT, ALP, ROS, and viscosity, respectively (Tables S7–S11).

### Prediction and screening of optimized probes

**Fig. 3.**
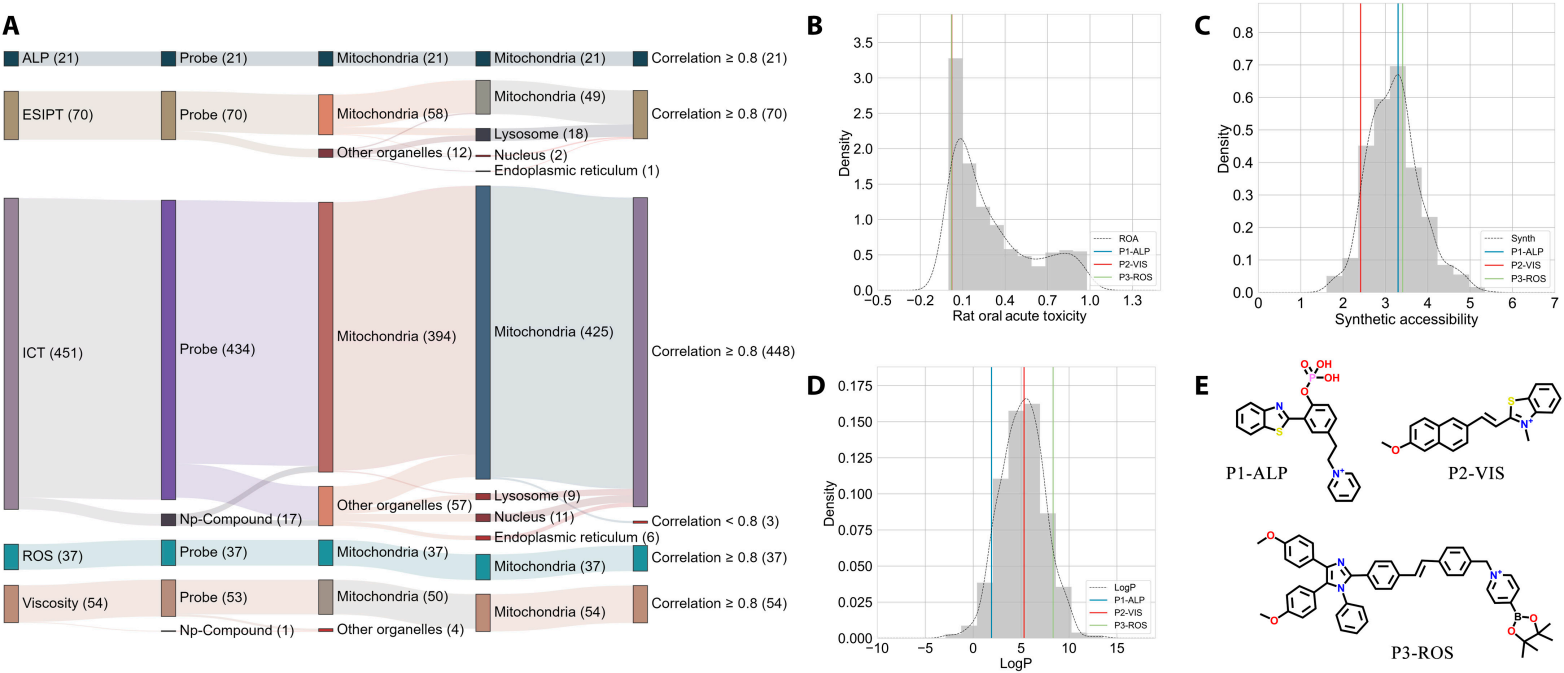
The prediction results of the designed probes by our multilevel framework. (A) The Sankey diagram displayed the predicted categories and the relationship between different models for ESIPT, ICT, ALP, ROS, and Viscosity sets. (B to D) The distribution of the important properties (rat oral acute toxicity, synthetic accessibility, and logP) for P1-ALP, P2-VIS, and P3-ROS compared with all designed probes. Here, rat oral acute toxicity represents the probability of a compound being of high toxicity; synthetic accessibility estimates the ease of synthesis of drug-like molecules, from 1 (very easy) to 10 (very difficult); logP represents the logarithm of the n-octanol/water distribution coefficient. The value of 3 was usually used as a threshold, and a higher one means high lipophilicity. (E) The molecular structures of P1-ALP, P2-VIS, and P3-ROS.

The molecular library mentioned above was fed into our multilevel framework to predict their organelle-targeting and colocalization effects. The predicted results are shown in Fig. 3A. We can see the prediction accuracy was quite good. In the first level, all structures in the ALP, ESIPT, and ROS sets were predicted as fluorescent probes by the B-PvsC model. In the ICT and viscosity sets, 17 and 1 structures were predicted as Np-compounds, respectively. This may be because some probes merely consist of a single fluorescent group but do not have obvious feature or recognition groups, so they are similar to Np-compounds. The second and third levels are the B-MvsP model and the M-PvsP model, respectively, which were used to classify the organelle targeting of fluorescent probes. As shown in the results, all structures in the ALP and ROS sets were similarly predicted as mitochondria-targeted both in the B-MvsP model and in the M-PvsP model. For the ESIPT set, no probes were predicted as Np-compounds as expected. In the B-MvsP model, only 12 probes were predicted as other organelle-targeted. Most of the 12 probes identified as other organelle-targeted by the second level were consistent with the third level while only 2 were reclassified as mitochondria-targeted. More importantly, most of the mitochondria-targeted ones predicted by the B-MvsP model were still kept there in the M-PvsP model, only 10 of them were predicted as lysosome-targeted, and 1 was predicted as nucleus-targeted. In the ICT set, only a few molecules were classified as Np-compounds and other organelle-targeted, while most of them were then back to be predicted as mitochondria-targeted by the M-PvsP model. As for the viscosity set, the M-PvsP model classified all structures as mitochondrial targeting, even though the B-MvsP model identified 4 other organelle-targeted. Similar to the ESIPT and ICT sets, the reason for this partial reassignment of structures to mitochondria-targeted by the M-PvsP model may be the tendency of classifiers to assign higher probabilities to categories with a larger sample size. Therefore, the simultaneous construction of binary and multiclassification models can compensate for the lack of fine classification and avoid an unbalanced prediction to improve the accuracy of the framework through a comprehensive evaluation. The last level is the B-McoL model that identifies the colocalization effect of mitochondria-targeted probes, and it was found that almost all designed probes possessed a good colocalization effect and only 3 probes in association with ICT might have poor colocalization effects.

To help us select the optimized probes, in addition to the rules summarized above, the physicochemical properties, synthetic accessibility, and safety were evaluated by an ADMETlab platform [24] for comprehensive screening and comparison. Among the dozens of properties from ADMETlab, rat oral acute toxicity could be a suitable indicator that estimates the biosafety of probes; synthetic accessibility could be a good indicator to balance the effectiveness and cost; logP could bridge the summarized rules (e.g., lipophilicity) and molecular structures in the form of numerical values. The probes retained after the filtering by our multilevel modeling were then fed into the platform. By analyzing the important properties and considering our experimental feasibility, we chose P1-ALP, P2-VIS, and P3-ROS from each set to forward. Figure 3B to D displays the distribution of rat oral acute toxicity, synthetic accessibility, and logP of the selected probes. It was shown that all the selected probes were low toxic with an acceptable synthetic difficulty. Among them, P1-ALP obtained a logP of 1.918 which was not so lipophilic but in the optimal range for a drug-like molecule.

### Quantitative calculation

**Fig. 4.**
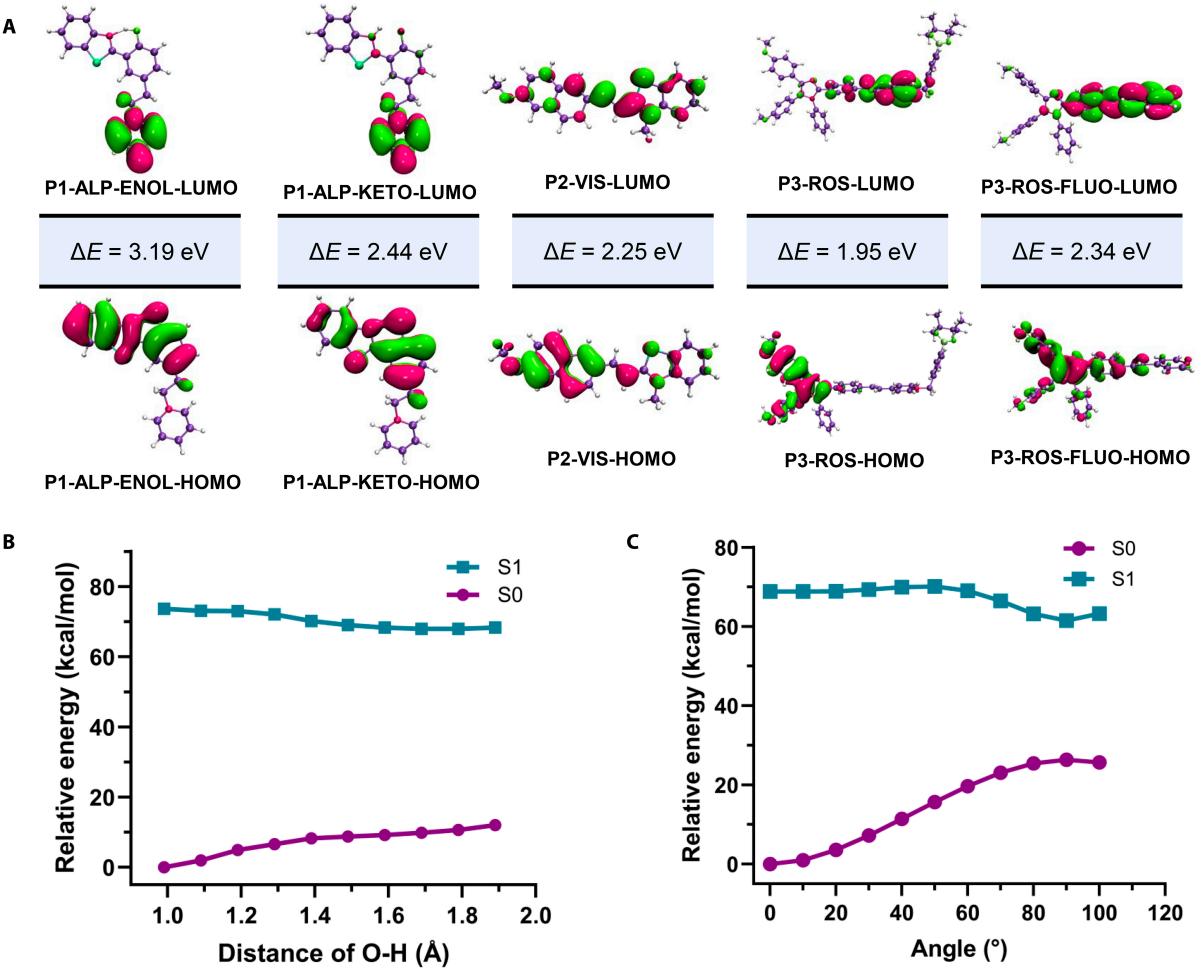
The quantitative calculation results of selected probes. (A) The molecular orbitals and excitation energy of selected probes and fluorophores. The P1-ALP-ENOL-HOMO means the highest occupied molecular orbital of P1-ALP in the enol form, and so on. Δ*E* represents the energy gap between the molecular orbitals. (B) The ESIPT process of P1-ALP. (C) Potential energy curve of TICT corresponding to P2-VIS. The ground-state structure of each compound was calculated by using Gaussian16 based on PBE0/Def2-SVP, and the excited state based on PBE0 (P1-ALP) and CAM-B3LYP (P2-VIS and P3-ROS). The single-point energy was calculated on the basis of PBE0/Def2-TZVP (P1-ALP) and CAM-B3LYP/Def2-TZVP (P2-VIS and P3-ROS).

We first optimized the ground-state structure of our selected probes and then calculated the ultraviolet (UV) absorption of each compound based on different functionals based on this structure. As shown in Table S6, we found that for each compound, the functional suitable for them was not the same because of the different nature of their respective charge transfer. So next, we performed optimization of excited states separately to obtain their fluorescence emission based on the functionals suitable for them. As shown in Table S6, according to the comparison of the calculated results with our experimental data, it was demonstrated that the method we chose can correctly describe the excited-state properties of each compound. Therefore, we performed further calculations on each compound separately to obtain their respective characteristic data. First, the ESIPT properties were calculated for P1-ALP. As shown in Fig. 4, according to our calculation results, there was no energy barrier to overcome for the occurrence of ESIPT, so the fluorophore can be rapidly converted from an enol to a keto structure after being excited. The calculated excitation energy of the enol form is 3.19 eV, and the excitation energy of the keto form emission is 2.44 eV, which was also in good agreement with our experimental data (3.30 and 2.55 eV). This indicated that, for P1-ALP, our calculations can adequately describe our experiments. Then, we calculated the TICT properties of the viscosity probe P2-VIS. Through the potential energy surface scan, we can find that the excited P2-VIS can undergo further distortion without an energy barrier to reach the state of TICT, which corresponded to the molecule. There was no fluorescence emission phenomenon in the state of a dilute solution, and then because of environmental constraints, P2-VIS will be forced to stay in the state of planar intramolecular charge transfer (PICT) without further relaxation. According to the calculation, the excitation energy of PICT of P2-VIS was 2.25 eV, which was basically consistent with the fluorescence emission observed in our experiments (2.25 eV). For P3-ROS, we found that the fluorescence wavelength of the probe appeared blue-shifted after the response, so we calculated the fluorescence emission of P3-ROS before and after the response. According to our calculation results, the excitation energy of the probe after the response was 2.34 eV, which was also consistent with our experimental data (540 nm, 2.29 eV, respectively).

### Synthesis of P1-ALP, P2-VIS, and P3-ROS

The detailed synthesis process and schematic diagram can be found in the Supplementary Materials.

### Characterization of P1-ALP and the response to ALP

**Fig. 5.**
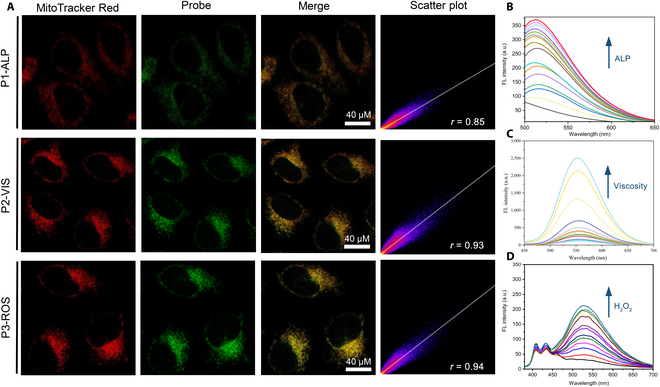
The optical characterization and mitochondrial colocalization imaging of selected probes. (A) The cell imaging for P1-ALP, P2-VIS, and P3-ROS. From left to right, they are the mitochondrial fluorescence image of MTR, the mitochondrial fluorescence image of the probe, the respective merge image, and the corresponding scatter plot. The scale was 40 μM. The “*r*” represents Pearson correlation coefficient. (B) The fluorescence spectrum of P1-ALP response to different concentrations of ALP. (C) The fluorescence spectrum of P2-VIS response to different viscosities. (D) The fluorescence spectrum of P3-ROS response to different concentrations of hydrogen peroxide. a.u., arbitrary units.

After obtaining P1-ALP, we first measured the UV spectrum and fluorescence spectrum of P1-ALP. As shown in Fig. S6, when the probe was added with LAP, the fluorescence emission of the system was about 390 nm. After incubation with ALP, a new and distinct fluorescence emission peak at 510 nm appeared in the spectrum, thus indicating that the probe P1-ALP could respond to ALP. According to the response time study, P1-ALP could respond completely to ALP within 28 min, which demonstrated high efficiency. To explore the environmental impacts, we first implemented a pH-effect experiment. As shown in Fig. S8, the probe P1-ALP could be used for detecting ALP over the pH value of 5 to 9 which proved the availability in the physiological environment. Therefore, our subsequent detection environment was phosphate-buffered saline:dimethyl sulfoxide (pH = 7.4). The probe and ALP were incubated in a water bath at 37 °C for 30 min. Additionally, according to the results of anti-interference experiments, the probe P1-ALP showed a specific response to ALP and good anti-interference performance (Fig. S7). Therefore, given the favorable characteristics, we tried to establish the standard detection curve. As shown in Fig. 5B, according to the results of the probe's response to different concentrations of ALP, the probe exhibited good linearity over the 0 to 120 U/l of ALP concentration. Generally, the above results indicated that P1-ALP could be used for specific quantitative detection of ALP, which provided a solid foundation for our subsequent imaging experiments.

### Characterization of P2-VIS and the response to viscosity determination

After obtaining P2-VIS, we measured the UV and absorption of P2-VIS. As shown in Fig. S18, the maximum absorption of P2-VIS is 550 nm. Because of the strong TICT effect of P2-VIS, P2-VIS basically has no fluorescence emission. Subsequently, we measured the fluorescence intensity of P2-VIS at different viscosities. As shown in Fig. 5C, fluorescence at 550 nm gradually increased with the increasing viscosity. The fluorescence intensity of the probe had a good phenomenon in the viscosity range of 2.66 to 1,319 cp, which indicated that P2-VIS could be satisfactory for in vivo viscosity imaging.

### Characterization of P3-ROS and the response to hydrogen peroxide

Similarly, for P3-ROS, we measured the UV and fluorescence spectra of the probe, and the results showed that the maximum absorption of P3-ROS was 425 nm (Fig. S24). After P3-ROS reacted with hydrogen peroxide, the UV absorption was 360 nm, and the fluorescence emission was 535 nm (Fig. S25). We measured the pH effect, selectivity, and response time respectively (Figs. S22 to S23). It was shown that the probe enabled a specific response to hydrogen peroxide in the pH range of 6 to 9 within 30 min. According to the response experiment of P3-ROS to hydrogen peroxide at different concentrations, the probe P3-ROS could detect quantitatively hydrogen peroxide between the hydrogen peroxide concentration of 0 to 10 μM (Fig. 5D).

### Bioimaging

After the success of in vitro response of the above 3 probes, further experiments were conducted for cell imaging, to verify the ability to target mitochondria. As shown in Figs. S10 and S27, P1-ALP and P3-ROS can be successfully used to image high intracellular ALP expression and ROS, respectively. We used MTR as a mitochondrial colocalization dye to evaluate the mitochondrial localization ability of the 3 probes. Consistent with our expectation, as shown in Fig. 5A, the colocalization coefficient of P1-ALP, P2-VIS, and P3-ROS with MTR reached 0.85, 0.93, and 0.94, respectively. Together, we successfully verified the accuracy of the mitochondrial targeting ability of the probes we screened based on our artificial intelligence framework.

## Discussion

When analyzing the 4 constructed models, we found that these models all achieved quite good performance that realized a reasonable multilevel evaluating framework. Both the B-PvsC and B-McoL models reported ACC reaching a maximum of 0.963 and a minimum of 0.933. In the B-MvsP model, the Recall values of mitochondria-targeted and other probes were 0.869 and 0.800, respectively. We can find a closer sample size between them (199 and 125). However, in the M-PvsP model, the sample size gap between categories increased obviously, resulting in categories with smaller sample sizes (e.g., Golgi apparatus) being more likely to be misclassified to the category with a larger proportion of sample size (e.g., mitochondria). This is because the classifier calculates the probability of each sample falling into each class in the prediction and determines which class the sample most likely belongs to by comparing the maximum of the 6 probabilities. Since the ROC curves are class skew independent, but related closely to the probability the classifier assigns to all samples in each class, it is clear that the AUC values for each class in the test set are still high (Fig. S4C). Thus, the M-PvsP model we constructed did successfully distinguish 6 types of organelle-targeting probes from the others. In this multiclassification model with imbalanced sample distribution, the probability values may reveal its true categories better than the output labels.

On the basis of the above statistical calculations, it was indicated that the positive charge was important for mitochondria-targeting molecules, which was in accordance with the mechanistic studies that positive charges could be pumped into mitochondria driven by mitochondrial membrane potential [37]. This explains that most of the current mitochondria-targeting probes are positively charged. Second, since triphenylphosphine is a relatively mature specific site for targeting mitochondria, this leads to the high importance of the P atom in our calculation results [7]. Third, the calculation results suggested that lipophilicity plays a more important role, as molecules with stronger lipophilic fragments can better cross the phospholipid membrane [37].

It was worth noting that the probes targeting the nucleus and lysosome were also positively charged, which means that the specificity of the mitochondria-targeted molecules could not rely only on the explanation based on the positive charge. In terms of molecular structure, the molecules targeting lysosome are highly similar to those targeting mitochondria, which makes it difficult to distinguish them from the view of the structure. Moreover, there were some special fluorescent molecules with multiple targeting abilities, which also affects the ability of predicted molecules to target mitochondria. These phenomena remind us that the probe design based on the consideration of multiple important factors should be better and a more refined multiclassification model may help.

Given the abovementioned mechanisms and analysis, it should be pointed out that there is still more that can be done to improve the predicting ability. First, the limited reaction types and structural scaffolds restricted the application domain of the models, which can easily lead to misclassification for the organelle categories with a small amount of data mentioned above. It suggests that we need to enlarge the data size of the model, which is a challenge in itself. Secondly, under the current prediction framework, we can combine the prediction results with structural similarity to make a better decision regarding the confusing organelle-targeted ones. Finally, the development of some new descriptors capable of elucidating the mechanism of fluorescent molecules will help to characterize the structure of such molecules, thereby improving the accuracy and specificity of the models.

During the screening of optimized probes, almost all designed molecules were predicted to have good mitochondrial targeting ability, as the molecular design of this library was based on the inspiration and rules obtained from the above artificial intelligence models. However, it was this situation that posed a challenge for us to select more reasonable probes. Therefore, we need to consider not only the targeting ability of these probes but also some important additional properties, as well as the complexity of the physiological environment and the synthesis conditions in the laboratory. For example, here, the logP value of P1-ALP was not prominent in the whole library, but it was still selected after comprehensive consideration. Notably, the colocalization experiments showed a colocalization coefficient (0.85) of P1-ALP lower than that of P2-VIS (0.93) and P3-ROS (0.94), which confirmed the rule we concluded: Lipophilicity was friendly to mitochondrial targeting.

According to our experimental results, the compound structures we predicted on the basis of the above descriptors have good accuracy for fluorescent molecules with different fluorescence mechanisms. The results of our synthesized P1-ALP, P2-VIS, and P3-ROS with ESIPT, TICT, and ICT properties show that P1-ALP, P2-VIS, and P3-ROS are not only suitable for detecting respective analytes in vitro and in cell imaging but also can accurately target mitochondria for the imaging of each detected object in the mitochondria.

In summary, we proposed a new method that integrated artificial intelligence with quantitative calculation to enable the rational design of organelle-targeted fluorescent probes. We firstly collected high-quality datasets concerning the organelle-targeted molecules and established a multilevel prediction framework by systematically comparing different algorithms and molecular descriptors, from which we obtained the structural features and rules of fluorescent molecules targeting mitochondria. Then, we applied these rules to design a library of mitochondria-targeted fluorescent probes with different fluorescence mechanisms. After assessment based on the framework and important physicochemical properties, 3 optimal probes were selected and synthesized. Their fluorescence mechanisms were then verified by quantitative calculation. As expected, in the experiments, they successfully achieved mitochondria targeting and subsequently detect ALP, viscosity, and hydrogen peroxide, respectively. Therefore, we believe that this work not only provides a great reference value for seeking fluorescent probes related to subcellular imaging but also helps to make a step forward in the intelligent design of molecular probes.

## Materials and Methods

### Molecular representation

In this work, different kinds of molecular descriptors and fingerprints were calculated to represent the physicochemical properties and structural features of molecules. The MOE software (version 2018, Chemical Computing Group, Montreal, QC, Canada) was used to calculate 2D descriptors, which consists of 206 descriptors in continuous and discrete values. The RDKit [38] and CDK software [39] were used to generate MACCS, ECFP4, AtomPair, Pubchem, and CDK fingerprints. Detailed information about these fingerprints can be found in ChemDes [28].

### Machine learning algorithm

In order to explore the most suitable machine learning models for the designed multilevel framework, 10 classical algorithms that cover tree ensemble models and linear models were employed, including simple decision tree [40], random forest (RF) [41], adaptive boosting (AdaBoost) [42], categorical boosting (CatBoost) [43], gradient boosting tree (GBT) [44], eXtreme gradient boosting (XGBoost) [45], light gradient boosting machine (LightGBM) [46], extra tree (ET) [47], logistic regression (LR) [48] and linear kernel support vector machine (SVM) [49]. In addition, the MolMapNet [50] was chosen as a representative deep learning method for the comparison. These algorithms are either simple or complex, based on different principles, and have their own advantages. They have been successfully applied in different scenarios. They were implemented in a customized Python (3.8.8) environment equipped with scikit-learn (1.0.2), molmap (1.3.6), xgboost (1.6.1), catboost (1.0.5), and lightgbm (3.2.1). All models using classical algorithms were constructed with 80% of the randomly split data set as the training set and the remaining as the test set to evaluate the model performance. In addition, a 5-fold CV of the training set was performed to ensure the robustness of the models. For MolMapNet, the dataset was randomly split into training set, validation set, and test set by 8:1:1 ratio.

### Feature selection and explanation

A customized feature selection pipeline was adopted to deprecate redundant features and avoid unnecessary computational costs. MOE 2D descriptors with variance of zero were dropped. If high correlation (> 0.95) were found between 2 descriptors, one of them was reserved. An interpretable explanatory method named SHAP [51] was used to provide perspectives from the feature contributions in order to have a better understanding of the constructed model and the predictions. In this context, the Shapley values represent the feature contributions to each prediction and have an additive property to provide an overview of which features have the most contributions to a model. More importantly, Shapley values can shed light on the directional influence of a feature in a single prediction, which can help us to attribute prediction errors. Moreover, tree-based feature importance is also used to provide insights into the ranks of feature contributions in the model.

### Quantitative calculation methods

Firstly, we used MOE to perform a conformational search on the ground-state structure of the compounds. After obtaining the structure with the lowest energy, we optimized the ground-state structure of each compound based on PBE0/Def2-SVP, and then because of the different charge transfer properties of each compound, we performed the structural optimization of excited states based on PBE0 and CAM-B3LYP, respectively. After obtaining the most stable structure, we calculated the single-point energies based on PBE0/Def2-TZVP and CAM-B3LYP/Def2-TZVP, respectively, to obtain more accurate excitation energies. In the calculation of the potential energy surface, we use the flexible scanning method to obtain the ESIPT and TICT or PICT potential energy surface.

### Chemical experimental characterization

Unless otherwise stated, all reagents and chemicals were purchased from qualified suppliers with required purities. All glassware was dried before use. Nuclear magnetic resonance spectra were measured on a Bruker AVANCE III HD 500-MHz spectrometer with tetramethylsilane as the internal standard. Mass spectrometry was performed on an Agilent Technologies 6530 quadrupole time-of-flight liquid chromatograph-mass spectrometer. UV-vis absorption spectra were performed on a UV-2550 scanning spectrophotometer (Shimadzu, Japan). Fluorescent spectra were recorded on a Hitachi F-2700 equipped with a 1-cm quartz cell. Dynamic light scattering measurements were performed at 25 °C on Zestier Nano ZS (Malvern Instruments Ltd, UK).

### Bioimaging

MCF-7 cells and Hela cells were purchased from the Institute of Basic Medical Sciences (IBMS) of the Chinese Academy of Medical Sciences. The cells were cultured in Dulbecco's modified Eagle's medium supplemented with 10% fetal bovine serum and 1% antibiotics (100 U/ml penicillin and 100 μg/ml streptomycin) at 37 °C in a 5% CO_2_ atmosphere. Cells were seeded in petri dishes (35 mm, Biosharp) for overnight culture. Then, the solution of selected probes (10 μM) and MTR (1 μM) were added to the cells and incubated for 30 min at 37 °C. After washing with phosphate-buffered saline 3 times, cells were imaged with a laser scanning confocal microscope (Leica TCS SP8, Germany).

## Data Availability

All data needed to evaluate the conclusions in the paper are present in the paper and/or the Supplementary Materials. You can also get them via: https://github.com/ifyoungnet/ChemCOTar.

## Supplementary Materials

Supplementary Materials Section S1. Supplementary materials for structure identification and cell cytotoxic assays methods Section S2. Supplementary materials for modeling pipeline and application domain Section S3. Figs. S1 to S33 Section S4. Tables S1 to S13
