# Supplementary material for: Rational Design of Organelle-Targeted Fluorescent Probes: Insights from Artificial Intelligence
Source: Research (Wash D C). 2023 Mar 8;6:0075. doi: 10.34133/research.0075 (PMC10013958; doi:10.34133/research.0075)
Supplement: Supplementary Materials — Section S1. Supplementary materials for structure identification and cell cytotoxic assays methods Section S2. Supplementary materials for modeling pipeline and application domain Section S3. Figs. S1 to S33 Section S4. Tables S1 to S13 [file research.0075.f1.docx]

Supplementary Materials for

**Rational Design of Organelle-targeted Fluorescent Probes: Insights from Artificial intelligence**

Jie Dong^1^, Jie Qian^2^, Kunqian Yu^3^, Shuai Huang^1^, Xiang Cheng^1^, Fei Chen^1^, Hualiang Jiang^3^, Wen-bin Zeng^1, *^

^1^ Xiangya School of Pharmaceutical Science, Central South University, Changsha 410083, P.R. China.

^2^ National Engineering Research Center of Rice and Byproduct Deep Processing, School of Food Science and Engineering, Central South University of Forestry and Technology, Changsha 410004, P.R. China.

^3^ State Key Laboratory of Drug Research, Drug Discovery and Design Center, Shanghai Institute of Materia Medica, Chinese Academy of Sciences, Shanghai, 201203, P.R. China.

**This file includes:**

1. **Supplementary materials for structure identification and cell cytotoxic assays methods**
2. **Supplementary materials for modeling pipeline and application domain**
3. **Supplementary Figures S1 to S33**
4. **Supplementary Tables S1 to S13**

* Corresponding author.

Wen-bin Zeng: wbzeng@hotmail.com, Tel: +8613787103346

1. **Supplementary materials for structure identification and cell cytotoxic assays methods**

**1.1 Materials and instruments**

All chemical reagents were purchased from Energy Chemical Co., Ltd (Shanghai, China). All chemical reagents and solvents are analytically pure and do not require further purification. ^1^H-NMR and ^13^C-NMR spectra were recorded on Bruker AVANCEIII 500M spectrometer (Rheinstetten, Germany) and Bruker AVANCEIII 400M spectrometer (Rheinstetten, Germany). High-resolution mass spectroscopy (HRMS) was obtained on an Orbitrap Velos Pro LC-MS spectrometer (Thermo Scientific). UV–vis absorption spectra were measured on a Shimadzu UV-2450 UV-VIS spectrophotometer. Fluorescence spectra were measured with a HITACHI F-2700 fluorescence spectrophotometer (HITACHI, Japan). Fluorescence images of cells were acquired on a Leica TCS SP8 laser scanning confocal microscope (Germany).

**1.2 Cell cytotoxic assays**

The cells were inoculated into petri dishes with DMEM, 10% fetal bovine serum, 1% penicillin and 1% streptomycin. After inoculating HepG2 cells in a 96-well plate for 24 hours, they were exposed to probe THBTA of different concentrations (0-20 μM) for 24 hours, 20 μL of MTT solution was added and incubated for 6 h, the supernatant was aspirated, and 150 μL of DMSO was added to dissolve the purple crystals. Measure the absorbance at 490 nm with a microplate reader.

- 1. **Compound synthesis**

Synthetic route of probe P1-ALP.


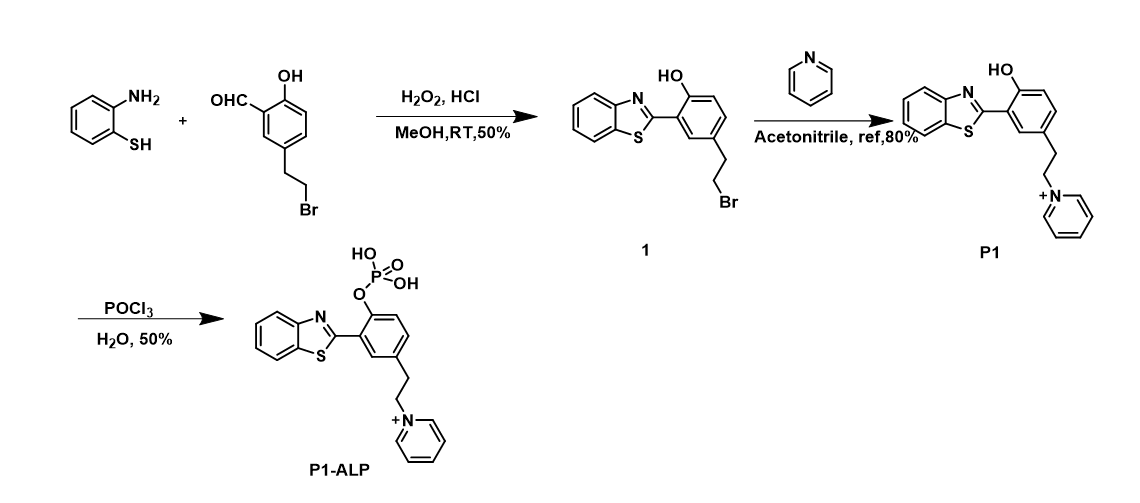


Synthetic route of probe P2-VIS.


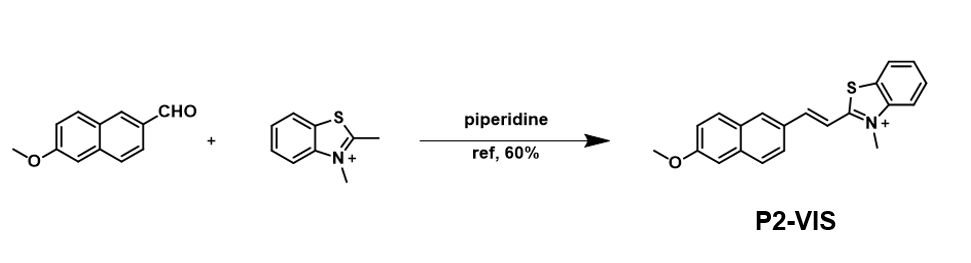


Synthetic route of probe P3-ROS.


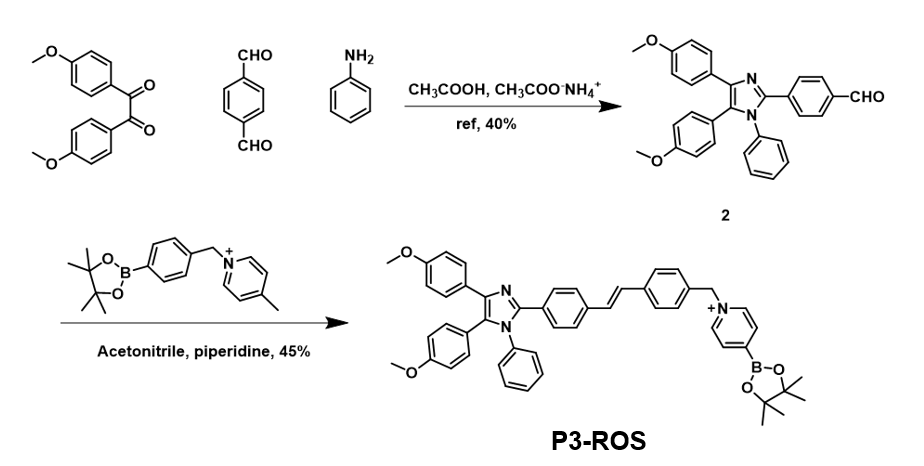


Synthesis of Compound **1**: Dissolve o-aminothiophenol (625 mg, 5 mM) and 5-(2-bromoethyl)-2-hydroxybenzaldehyde (1140 mg, 5 mM) in methanol respectively, add a small amount of hydrogen peroxide and hydrochloric acid, and stir the system at room temperature for 6 h. After the reaction was completed, the pH was adjusted to neutral, extracted three times with DCM, the organic layers were combined, dried over anhydrous magnesium sulfate, and then separated and purified by silica gel column to obtain product **1** (830 mg, 50%)^1^H NMR (500 MHz, CDCl3): δ: 12.45-12.47 (s, 1H), 7.98-7.96 (d, J = 3.5 Hz, 1H), 7.90-7.98(d, J = 3.5 Hz, 1H), 7.51-7.48 (t, J = 13.2 Hz, 2H), 7.42-7.39 (t, J = 10.3 Hz, 1H), 7.23-7.21(dd, J = 6.5 Hz, 1H), 7.06-7.04 (dd, J = 6.5 Hz，1H), 3.59-3.56 (t, J = 6.6 Hz，2H), 3.17-3.14(t, J = 6.0 Hz, 2H). ^13^C NMR (125 MHz, CDCl3): δ: 169.05, 156.93, 151.83, 133.12, 132.58, 129.96, 128.35, 126.77, 125.64, 122.24, 121.56, 118.16, 116.68, 37.141, 33.12, 31.44, 30.18.

Synthesis of Compound **P1**: Compound **1** (830 mg, 2.5 mM) was dissolved in acetonitrile, excess pyridine was added, and the system was refluxed at 90 °C for 4 h. After the reaction was complete, the system was distilled under reduced pressure and then separated and purified by silica gel column to obtain product **P1** (666 mg , 80%)^1^H NMR (CD3OD, 500 M Hz): δ: 8.94-8.93 (d, J = 6.0 Hz, 2H), 8.58-8.55 (t, J = 15.0 Hz, 1H), 8.09-8.06 (t, J = 14.0 Hz, 2H), 8.02-7.98 (t, J = 8.1, 2H), 7.60-7.59 (d, J = 6.0 Hz, 1H), 7.56-7.53 (t, J = 7.6 Hz, 1H), 7.47-7.44 (t, J = 7.5 Hz, 1H), 7.27-7.25 (dd, J = 5.0 Hz, 1H), 7.0-6.98 (dd, J = 5.05 Hz, 1H), 4.96-4.94 (t, J = 7.1 Hz, 2H), 3.38-3.35 (t, J = 7.0 Hz, 2H). HRMS(ESI) m/z: calculated [M-Br] ^+^:333.1057, found [M-Br] ^+^:333.1048。

Synthesis of Compound **P1-ALP**: Compound **P1** (333 mg, 1 mM) was dissolved in dry DCM (20 mL) and stirred at 0 °C. POCl_3_ (0.2 mL) and pyridine (0.3 mL) were added though syringe. After that, the reaction solution was stirred at room temperature for 3 h. Then poured into ice water (200 mL) and stirred overnight. and the reaction solution was extracted with DCM/EtOH. The combined organic phase was dried with Na_2_SO_4_, and concentrated under reduced pressure. After purified by the silica gel chromatography (DCM /EtOH, 3:1, v/v), compound **P1-ALP** was obtained as a colorless solid in 50 % yield. ^1^H NMR (500 MHz, CD3OD): δ: 8.95-8.93(d, J = 4.0 Hz, 2H), 8.29-8.25(t, J = 8.5 Hz,1H), 8.05-8.03(d, J = 4.0 Hz, 1H), 7.95-7.92(t, J = 6.0 Hz, 3H), 7.75(s, 1H), 7.47-7.44(m, J = 6.8 Hz, 2H), 7.09-7.07(d, J = 5.6 Hz, 1H), 6.85-6.83(d, J = 5.6 Hz, 1H,), 5.08-5.04(t, J = 7.1 Hz, 2H), 2.27-2.23(t, J = 6.1 Hz, 2H), 2.22(s, 6H). 31P-NMR (202 MHz, CD3OD): δ = -5.01. HRMS(ESI) m/z: calculated [M-Br] ^+^:413.0720, found[M-Br] ^+^:413.0717.

Synthesis of **P2-VIS**: 6-Methoxy-2-naphthaldehyde (931 mg, 5 mmol) and 2-Methylbenzothiazole salt (1.23 g, 7.5 mmol) were dissolved in 50 mL ethanol, and then piperidine (24 μL, 0.28 mmol) was added. The mixture was refluxed for about 12 h until the raw material disappeared. After cooling to room temperature, the reaction mixture was centrifuged, filtered and evaporated, then purified with column chromatography (silica gel, ethyl acetate: petroleum ether, 1:5, v/v) to yield probe 1 as an orange powder (yield: 65%). ^1^H NMR (400 MHz, DMSO-d6, ppm) δ/ppm: 3.92 (s, 3 H), 4.38 (s, 3 H), 7.26 (d, 1 H, J = 8.80 Hz), 7.42 (s, 1 H), 7.80 (t, 1 H, J = 7.60 Hz), 7.88 (t, 1 H, J = 7.80 Hz), 7.97 (t, 2 H, J = 9.00 Hz), 8.06 (d, 1 H, J = 15.60 Hz), 8.27 (m, 3 H), 8.46 (t, 2 H, J = 7.80 Hz); ^13^C NMR (100 MHz, DMSO-d6, ppm) δ/ppm: 36.9, 56.0, 107.0, 113.3, 117.3, 120.2, 124.7, 125.4, 128.2, 128.3, 128.5, 128.8, 129.8, 130.0, 131.2, 133.1, 136.9, 142.5, 149.3, 159.9, 172.3; HRMS m/z: Calcd. for [C_21_H_18_NOS^+^] 332.1104, found 332.1104.

Synthesis of Compound **2**: Compound terephthalaldehyde (1.34 g, 10 mmol) and aniline (1.116 g, 12 mmol) were added to a 50 mL round-bottomed flask, dissolved in 100 mL of glacial acetic acid, stirred at room temperature for 1.5 h, and then added to the system at room temperature Anisyl (2.7 g, 10 mmol) and ammonium acetate (5.4 g, 70 mmol) were heated to reflux at 90°C and monitored by TLC until the end of the reaction. The reaction was removed and cooled to room temperature, added dropwise to 200 mL of ice water, adjusted to near neutral pH with 0.1 mol·L-1 sodium hydroxide solution, resulting in a green precipitate, suction filtered, and washed with ice water 3 times to obtain a green solid . It was dried in a drying oven at 60°C, and was separated and purified by silica gel column chromatography (PE:EA=1:1) to obtain 1.50 g of yellow solid powder **2** with a yield of 40 %. ^1^H-NMR (500 MHz, CDCl_3_, TMS) δ: 9.96 (s, 1H), 7.73-7.75 (d, 2H), 7.59-7.61 (d, 2H), 7.53-7.55 (d, 2H), 7.30-7.32 (m , 3H), 7.03-7.08 (m, 4H), 6.81-6.83 (d, 2H), 6.76-6.78 (d, 2H), 3.79(s, 3H), 3.78 (s, 3H). ^13^C-NMR (125 MHz) , CDCl_3_): δ = 191.77, 159.36, 158.61, 144.85, 138.65, 136.93, 136.05, 135.34, 132.32, 131.06, 129.46, 129.37, 129.01, 128.68, 128.50, 128.35, 126.72, 122.31, 113.94, 113.70, 55.21, 55.15. m/z: calculated for C_30_H_24_N_2_O_3_, 460.1787; found [M+H]^+^: 461.1858.

Synthesis of Compound **P3-ROS**: Compound **2** (115 mg, 0.25 mmol) was added to a 50 mL round-bottomed flask, 10 mL of anhydrous EtOH was dissolved, compound PI (93 mg, 0.3 mmol) was added to the system at room temperature, and 20 μL of catalyst piperidine was added at 40 °C. The reaction was monitored by TLC until the end of the reaction. Cool to room temperature, evaporate the reaction system solvent anhydrous ethanol by rotary evaporator, and separate and purify by silica gel column chromatography (DCM:MeOH=10:1) to obtain 59.1 mg of red-brown solid **P3** with a yield of 45 %. ^1^H-NMR (500 MHz, d_6_-DMSO, TMS): δ = 9.09 (d, 2H), 8.23 ​​(d, 2H), 8.17 (d, 2H), 7.96-7.99 (d, 1H), 7.83-7.85 ( d, 2H), 7.64-7.66 (d, 2H), 7.53 (d, 1H), 7.42-7.47 (m, 5H), 7.35-7.39 (m, 3H), 7.28-7.30 (m, 2H), 7.14- 7.17 (d, 2H), 6.84-6.87 (dd, 4H), 5.77 (d, 2H), 3.73 (s, 6H), 1.24(s, 12H). ^13^C-NMR (125 MHz, d_6_-DMSO): δ = 159.51, 158.50, 145.15, 144.91, 137.72, 137.29, 136.58, 135.30, 132.92, 132.50, 131.30, 130.11, 129.75, 129.32, 129.21, 128.83, 128.44, 127.99, 127.94, 127.37, 124.70, 114.40, 114.14, 55.48, 31.73, 29.54, 29.47, 29.43, 29.28, 29.14, 29.03, 27.02, 22.54, 14.40. HR-MS (ESI), m/z: calculated :752.3654; found [M]^+^:752.3600.

1. **Supplementary materials for modeling pipeline and application domain**

The modeling and application domain service were provided in a GitHub repository (ChemCOTar): <https://github.com/ifyoungnet/ChemCOTar>

**ChemCOTar**


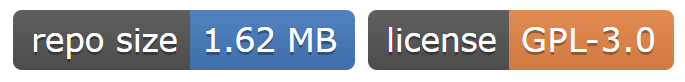

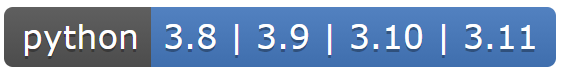


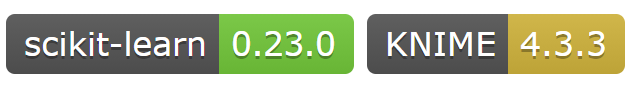


ChemCOTar aims to provide a multi-level framework to predict whether a compound is an organelle-targeted probe (B-PvsC model) and which organelles it might target (M-PvsP model).

##### This repository provides two workflows based on KNIME software:

1. Example workflow for multi-level prediction framework, including: a, B-PvsC model; b, B-MvsP model; c, M-PvsP model; d, B-McoL model. The detailed information about the four models could be found in the paper listed below.
2. Example workflow for application domain. Compare the S indices.TMean vs S indices.QMean and S indices.TMax vs S indices.QMax. We suggest that a molecule having a QMean within 2 fold standard deviation or higher QMax is more likely in the application domain. Users should analysis the result according to their own circumstances.

- S indices.TMean: Mean and standard deviation of the calculated mean similarity between each molecule and the whole training dataset (excluding itself).
- S indices.TMax: Mean and standard deviation of calculated max similarity between each molecule and the whole training dataset (excluding itself).
- S indices.QMean(Query_Mean): Mean of the calculated similarity between query molecule and the whole training dataset.
- S indices.QMax(Query_Max): Max of the calculated similarity between quary molecule and the whole training dataset.

| **Model** | **S indices.TMax** | **S indices.TMean** |
| --- | --- | --- |
| B-PvsC | 0.844±0.107 | 0.41±0.069 |
| B-MvsP | 0.861±0.091 | 0.444±0.058 |
| M-PvsP | 0.861±0.091 | 0.444±0.058 |
| B-McoL | 0.853±0.097 | 0.443±0.059 |

Here is a snapshot:
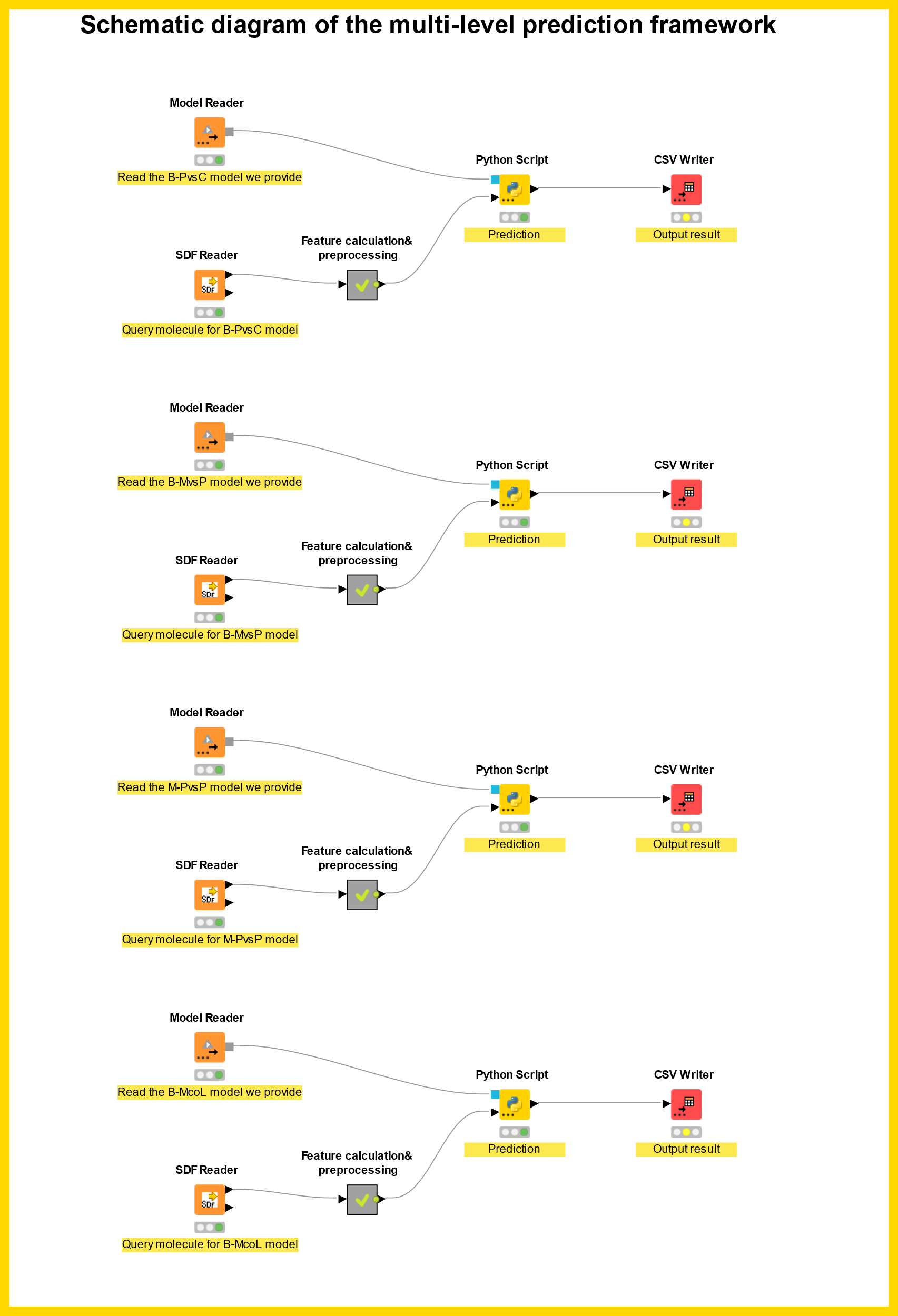


1. **Supplementary Figures S1 to S33**


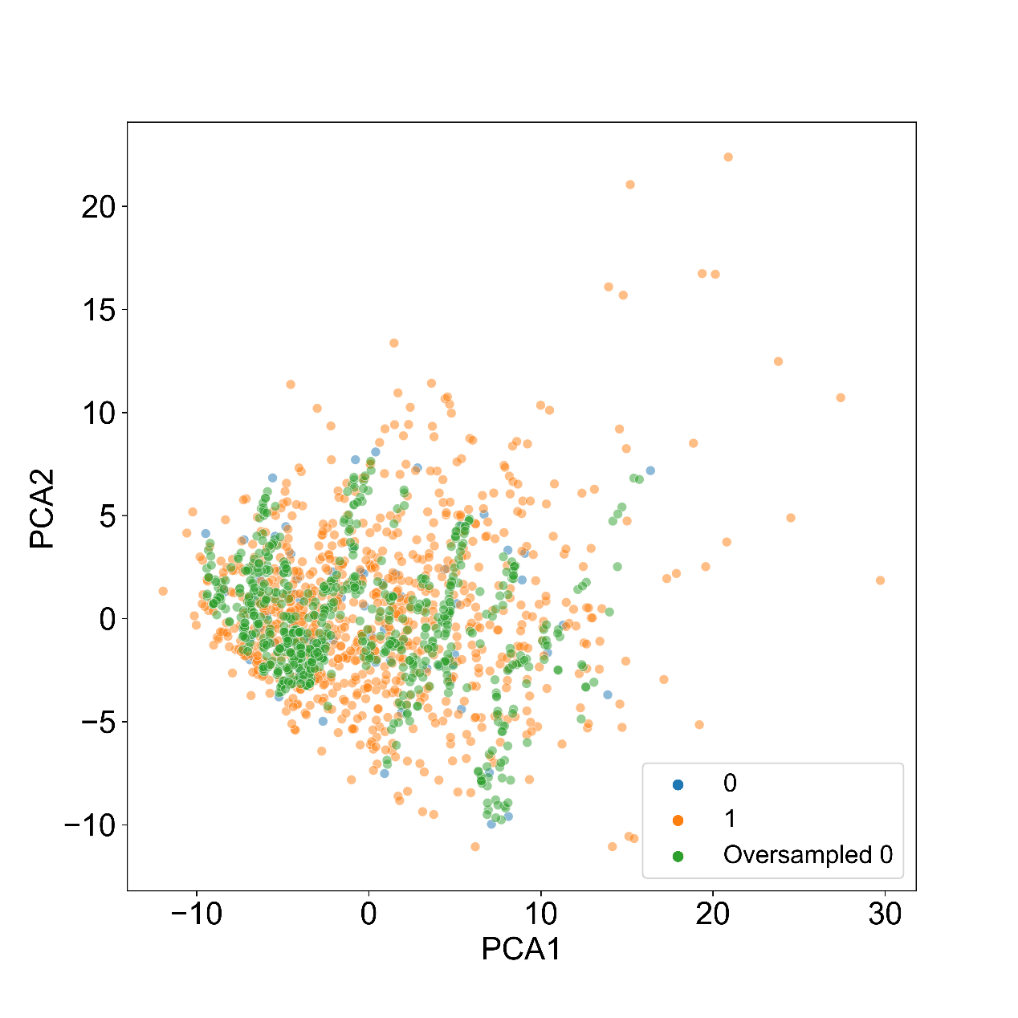


Figure S1.

Scatter plot of the B-McoL dataset after oversampling using SMOTE.


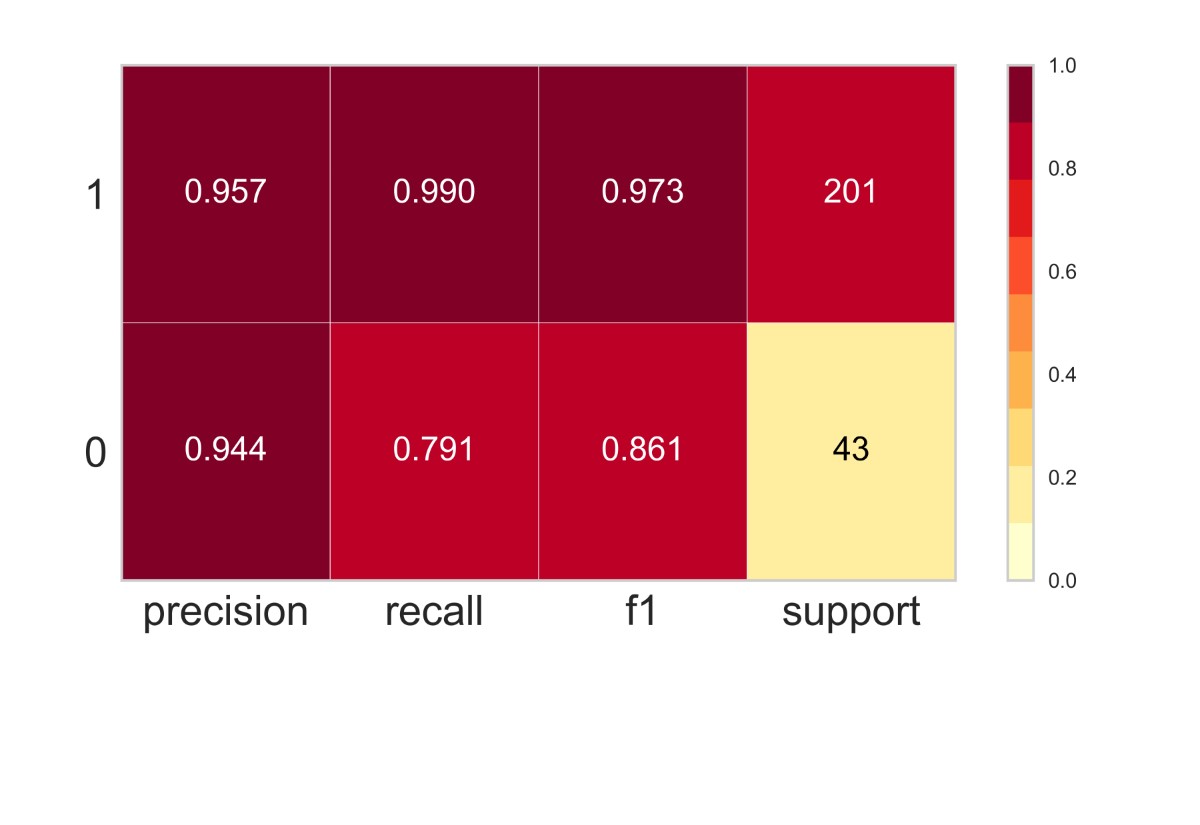

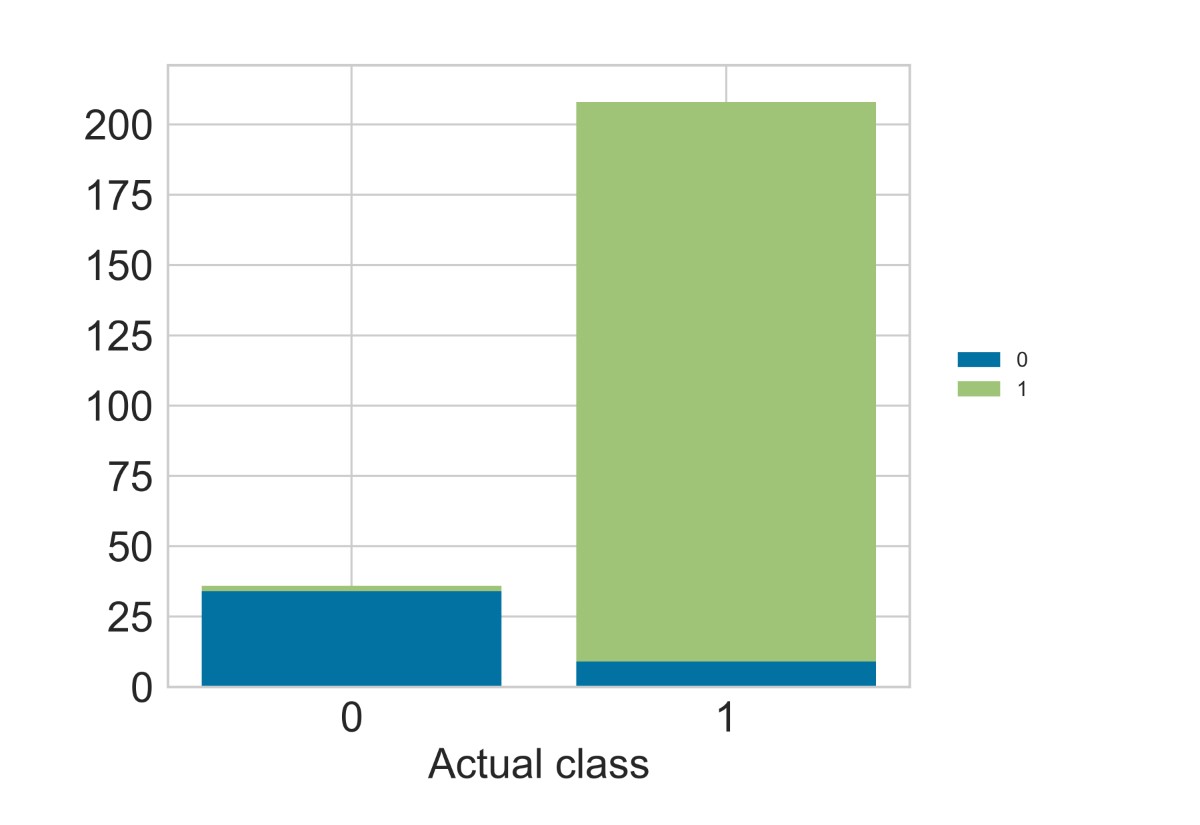


a

b

Figure S2.

(a) Classification report, (b) Prediction error plots of the best B-PvsC model (Class 0: Np-Compounds; Class 1: Probes).


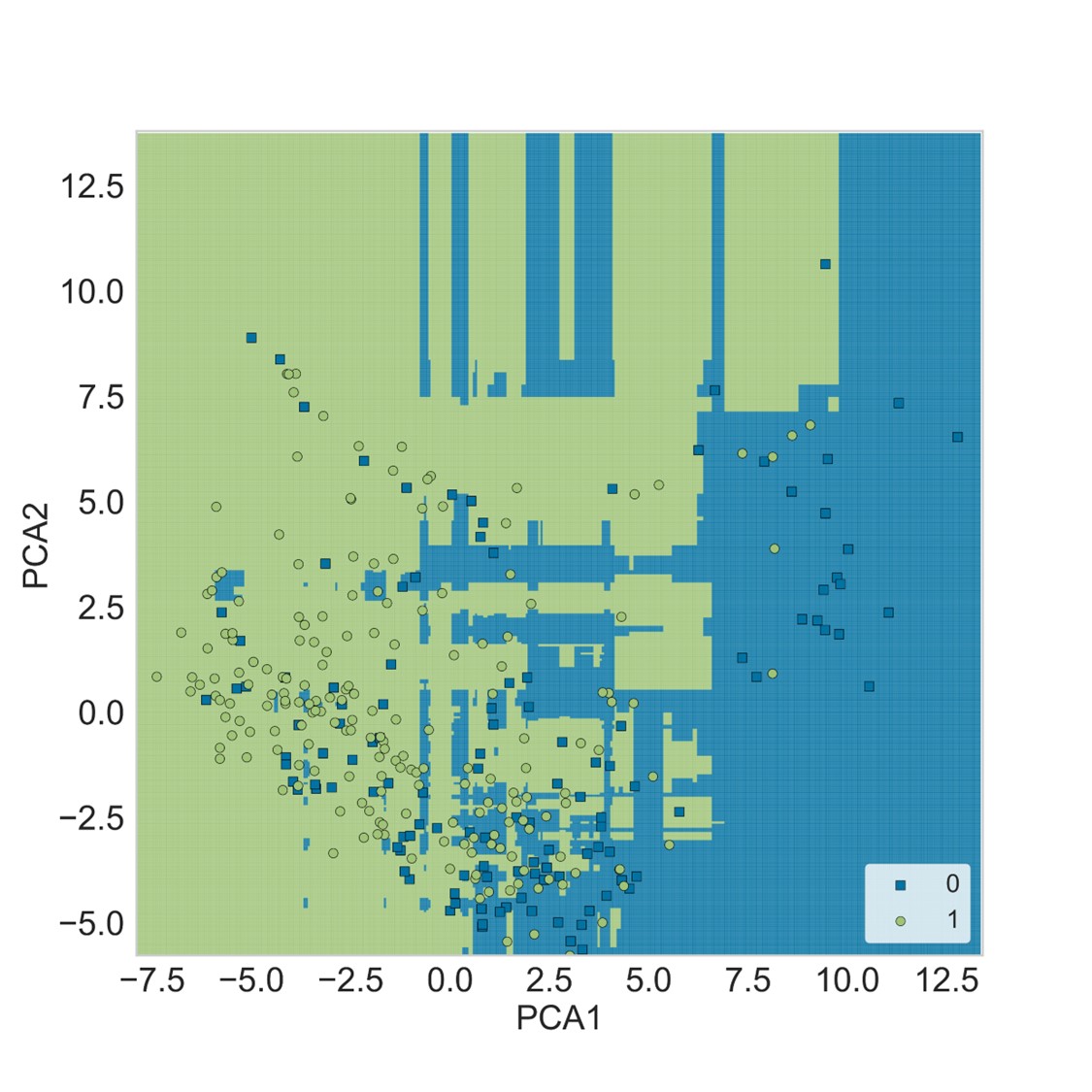

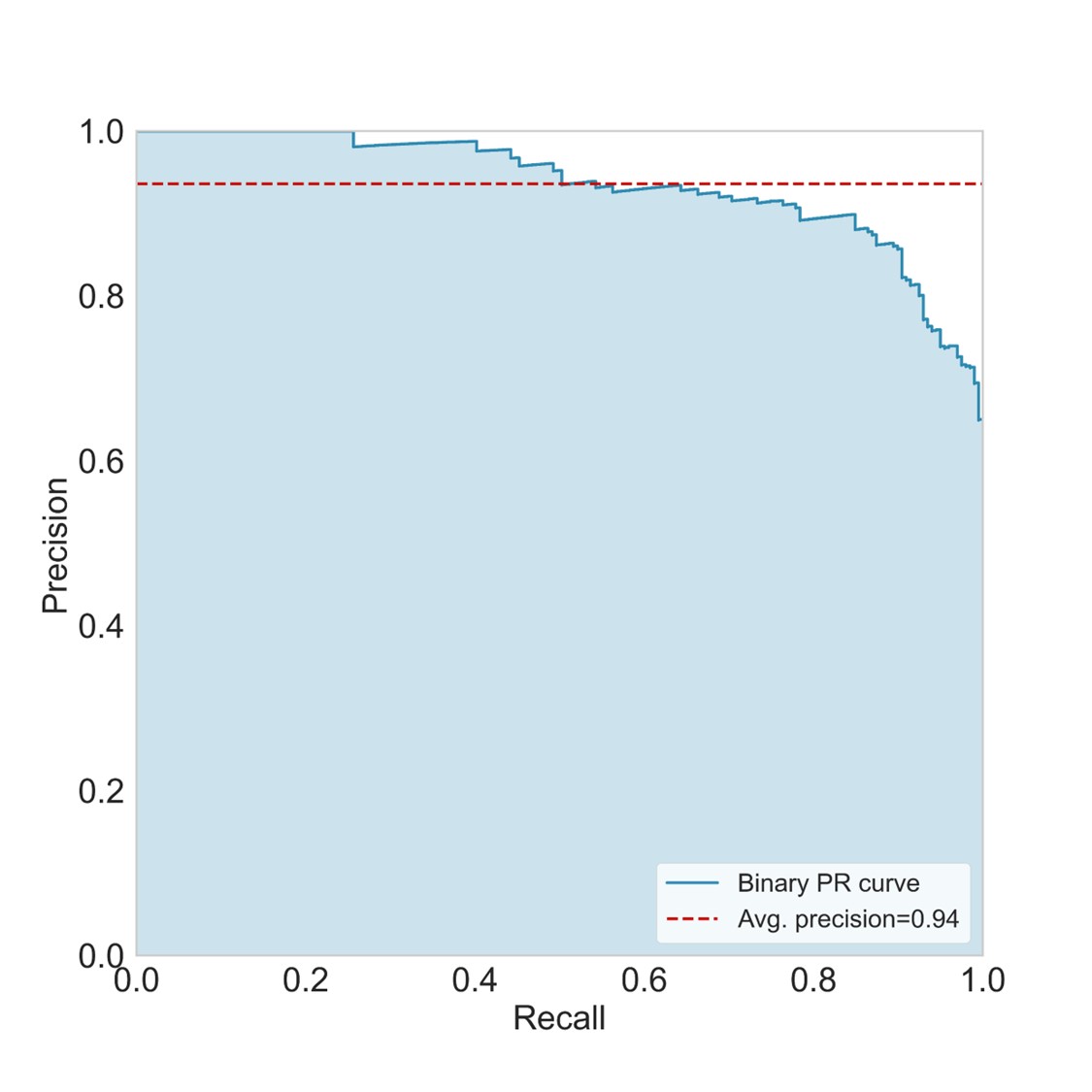

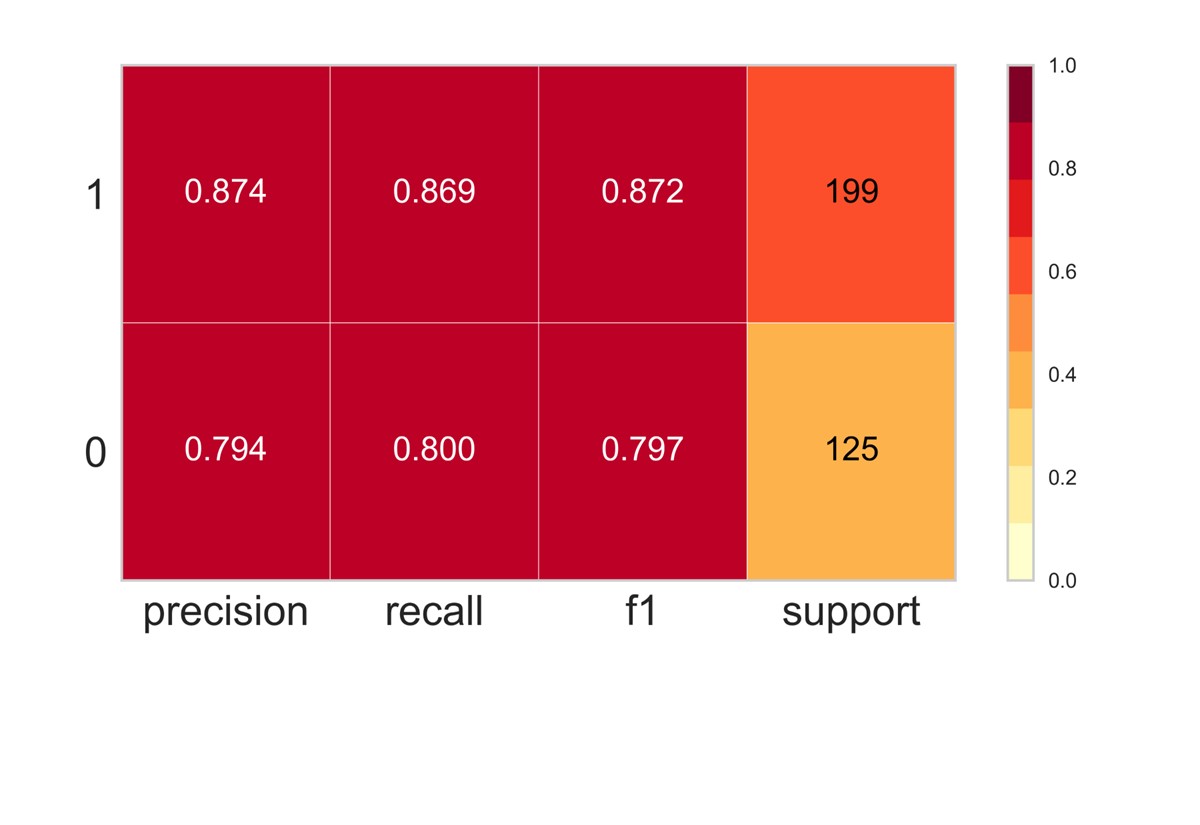

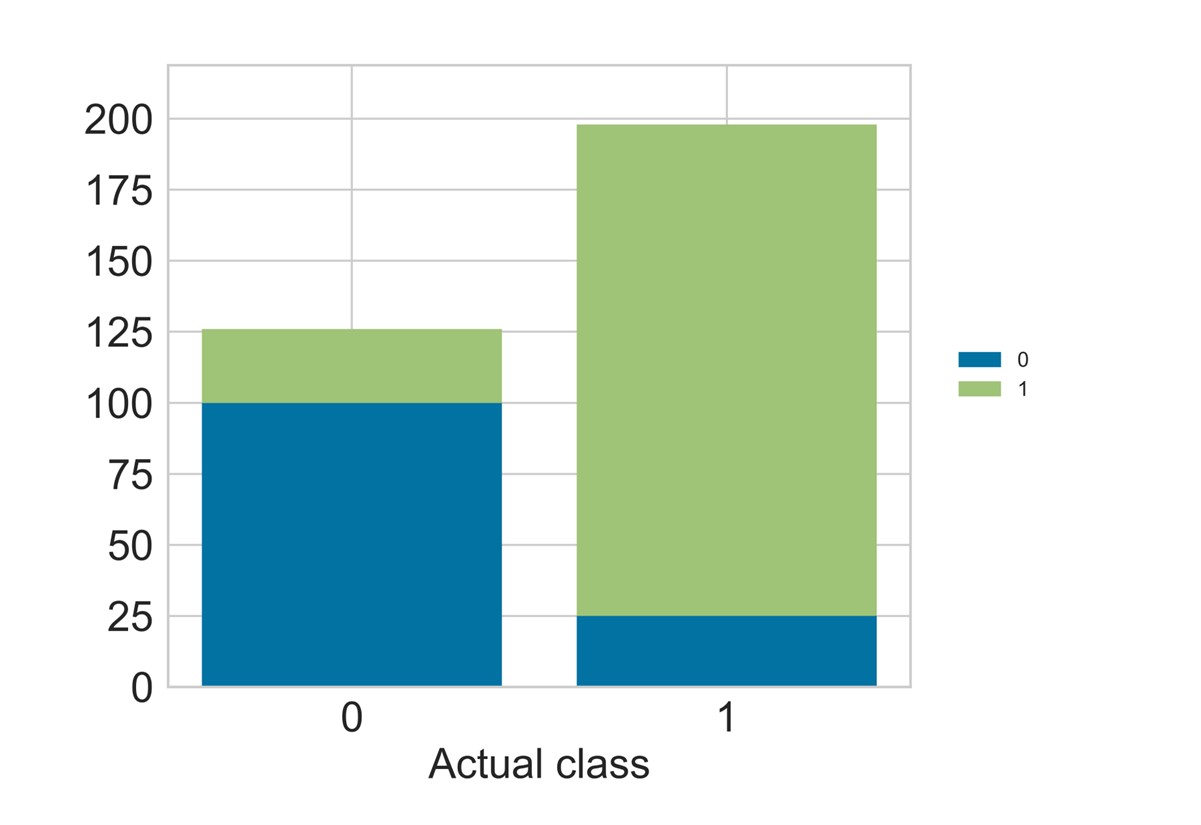


a

b

c

d

Figure S3.

(a) Classification report, (b) Prediction error, (c) Precision recall curve, and (d) Decision boundary plots of the best B-MvsP model (Class 0: Other organelle-targeted probes; Class 1: Mitochondria-targeted probes).


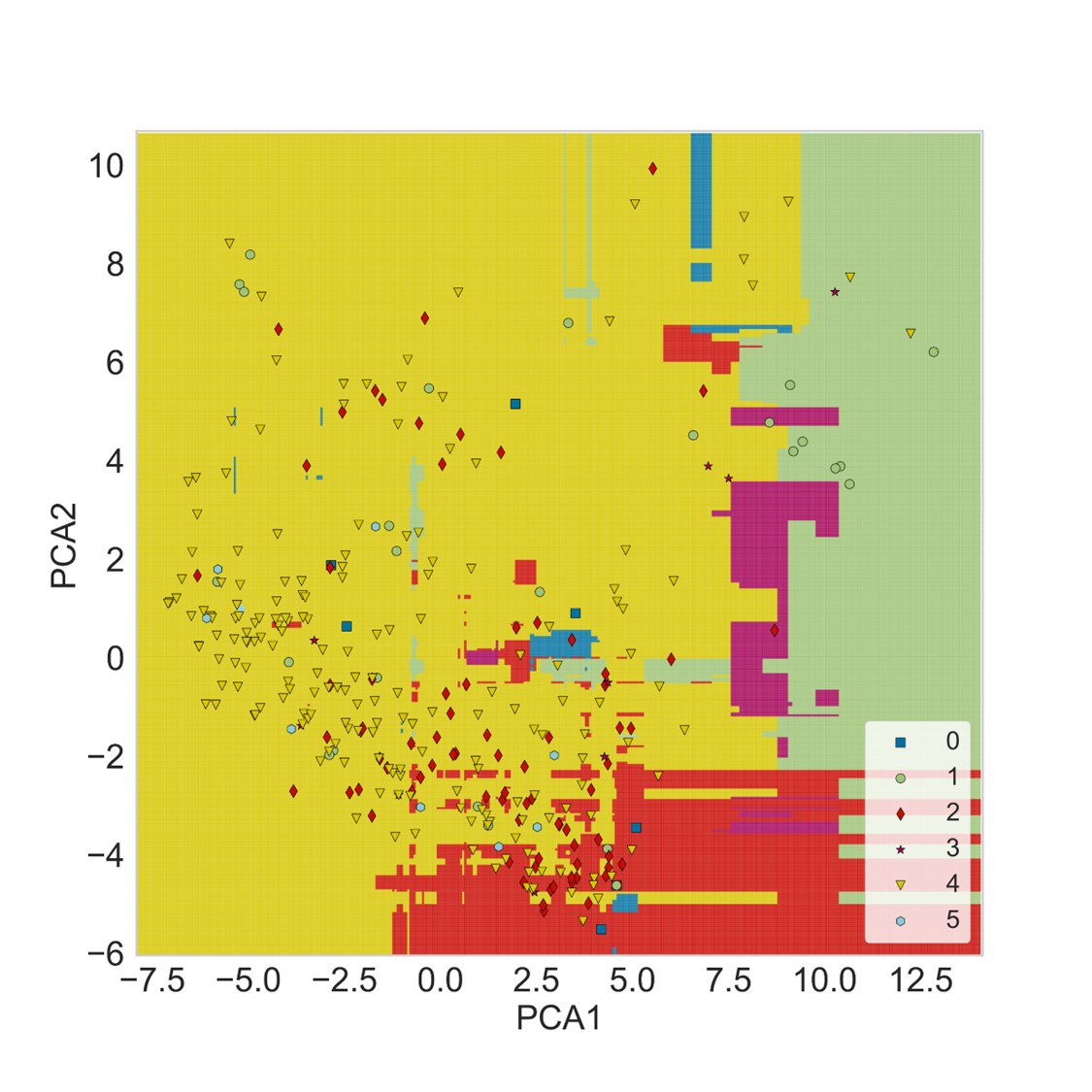

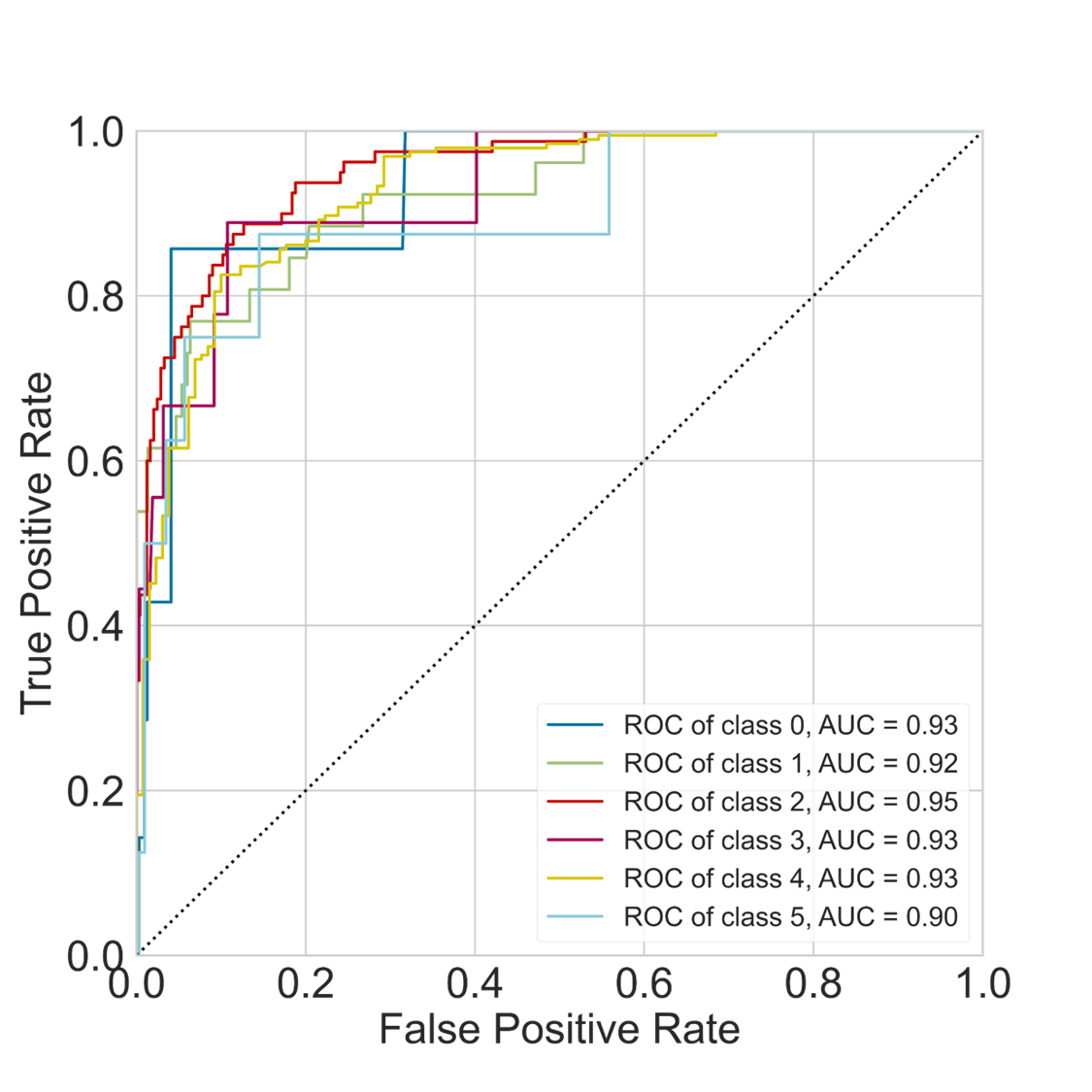

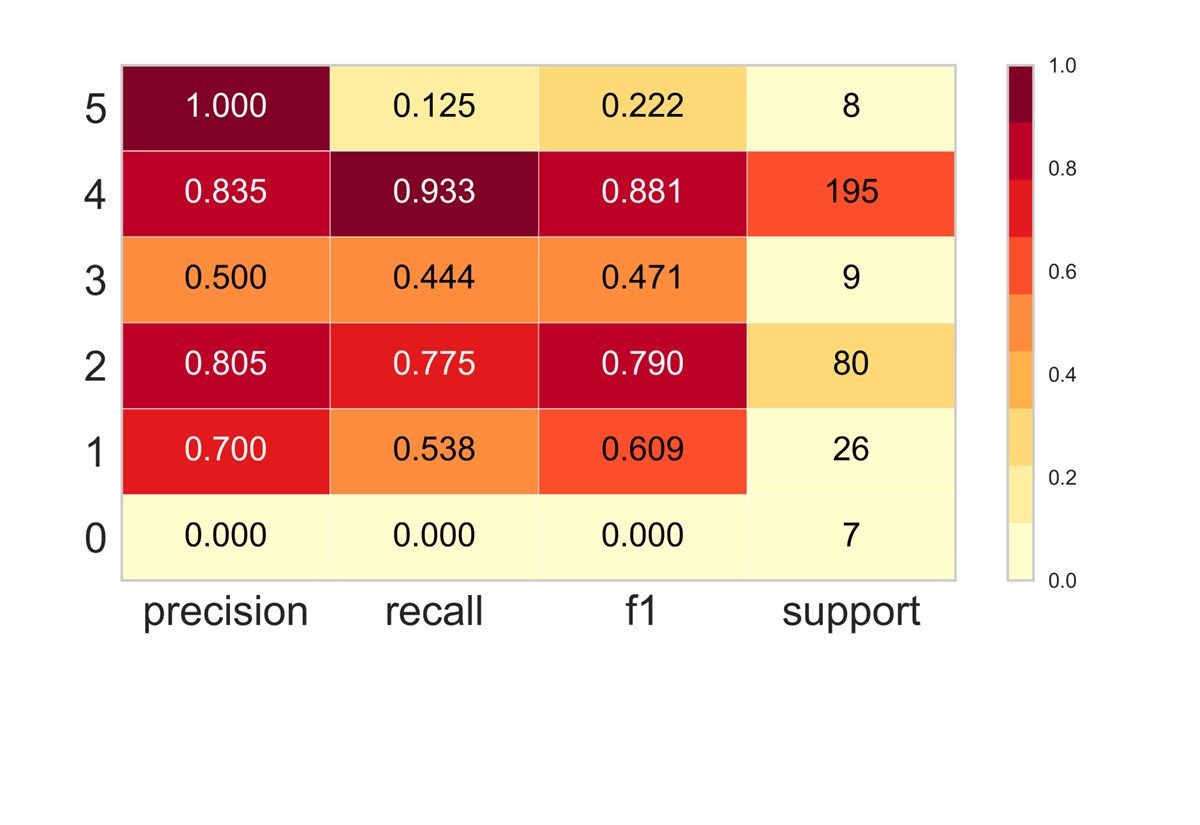

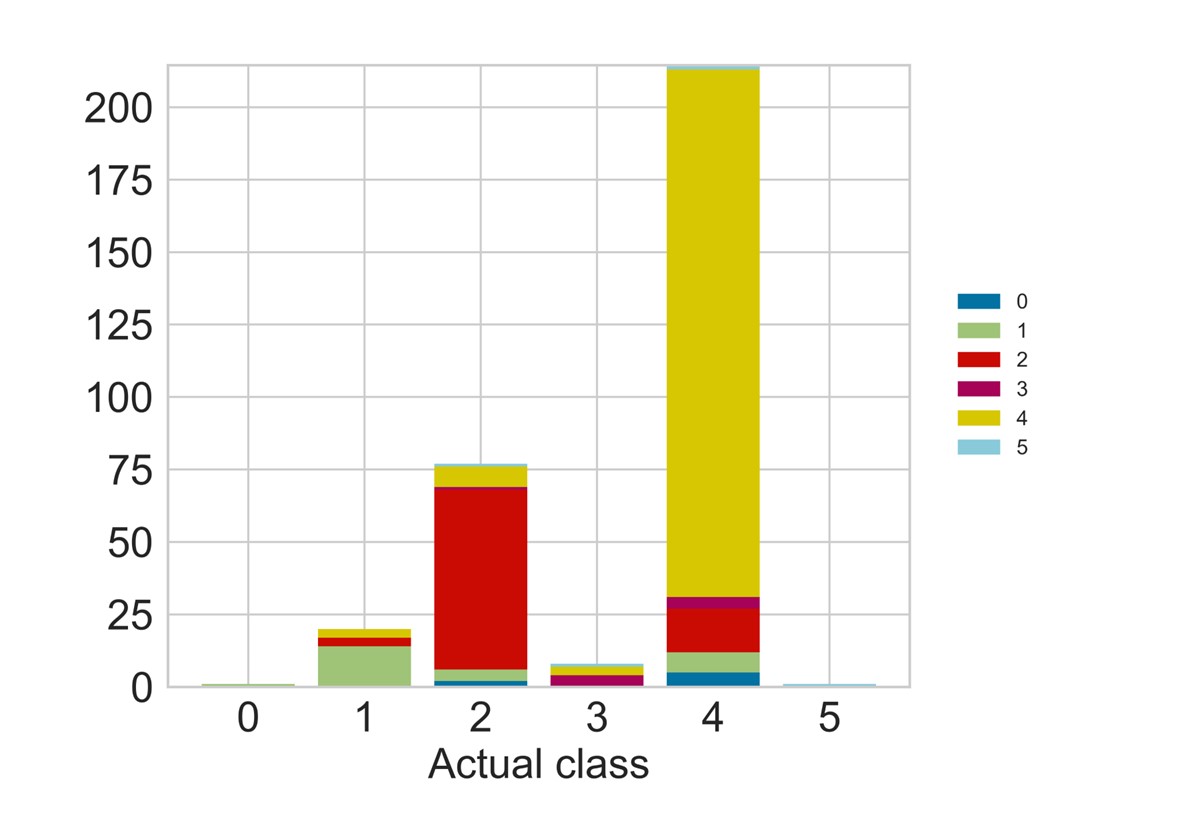


a

b

c

d

Figure S4.

(a) Classification report, (b) Prediction error, (c) ROC curve, and (d) Decision boundary plots of the best M-PvsP model (Class 0: Golgi apparatus-targeted probes; Class 1: Endoplasmic reticulum-targeted probes; Class 2: Lysosome-targeted probes; Class 3: Cell membrane-targeted probes; Class 4: Mitochondria-targeted probes; Class 5: Nucleus-targeted probes).


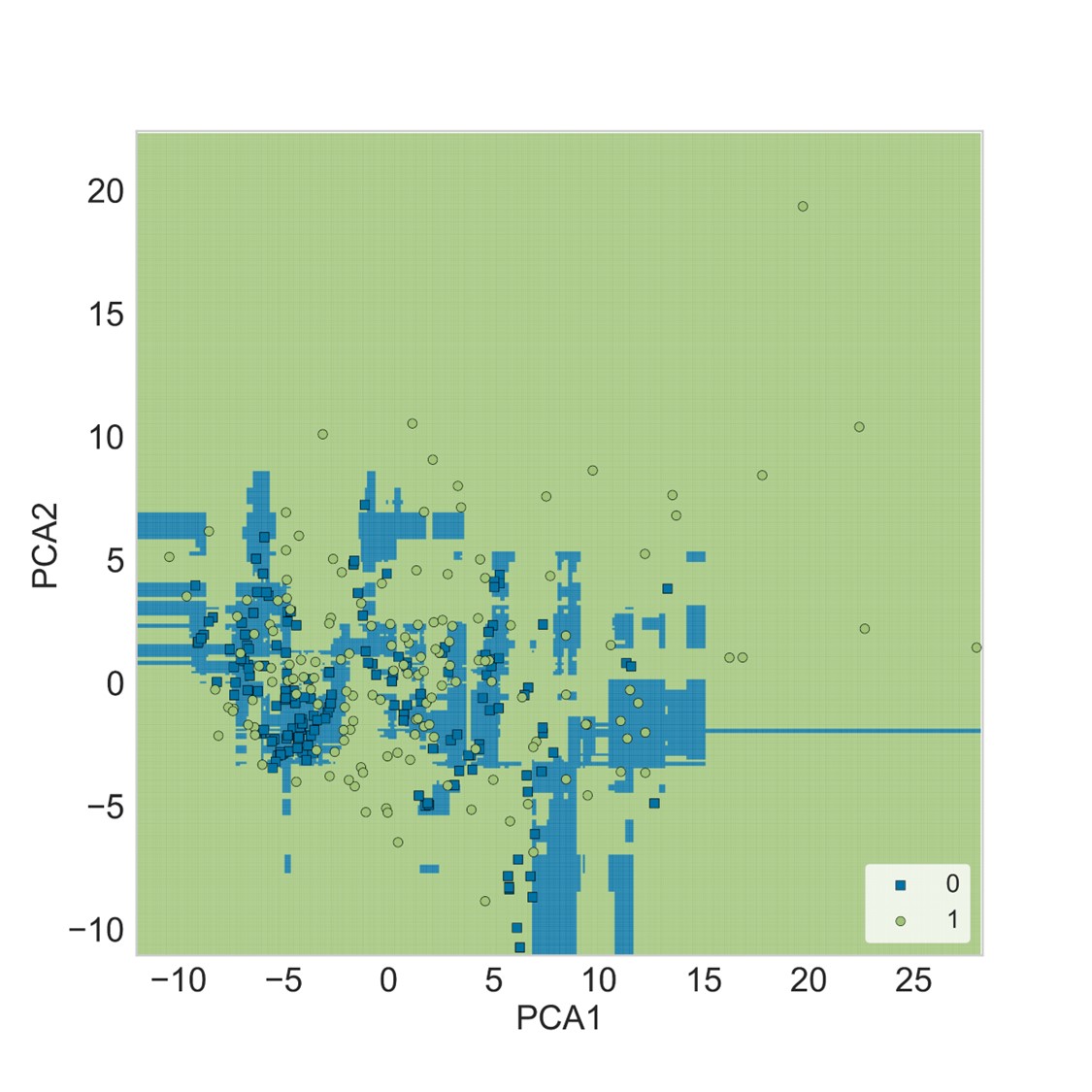

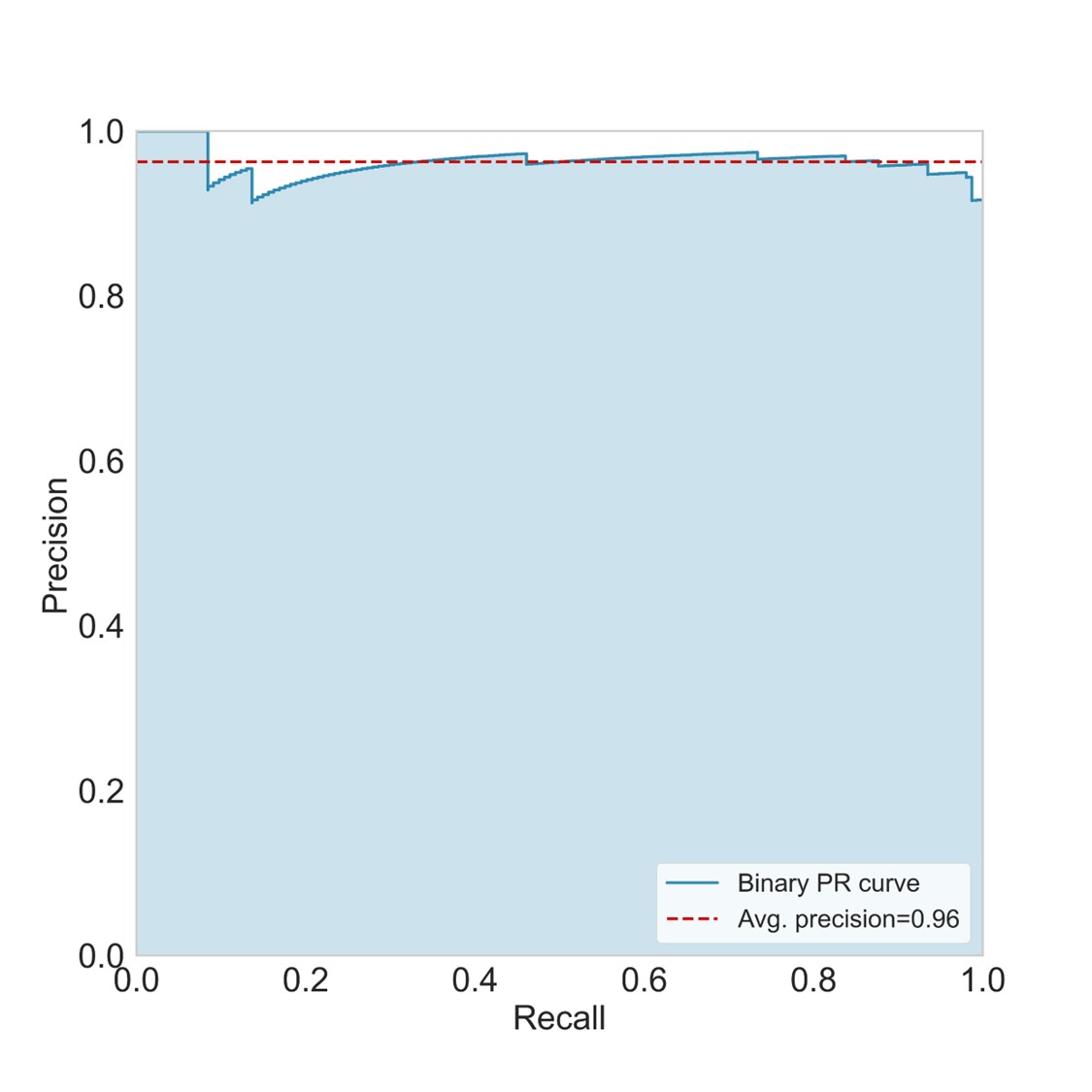

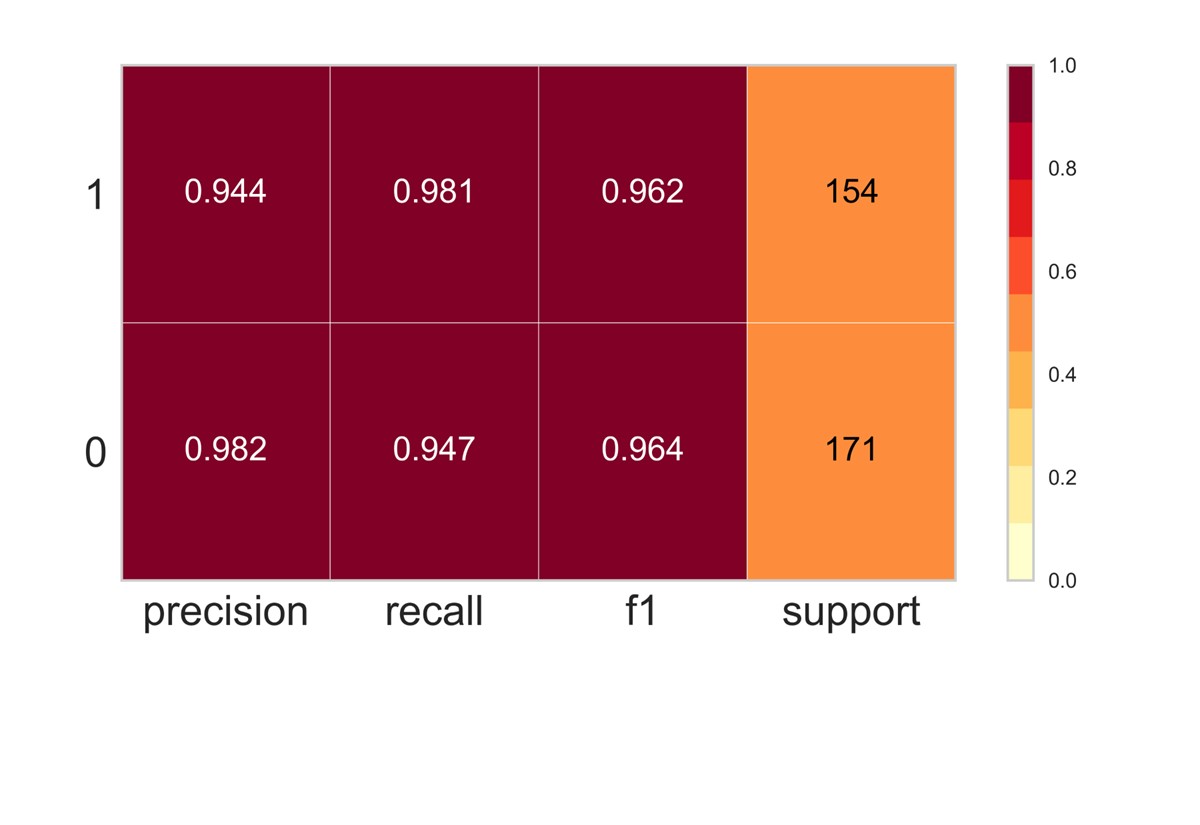

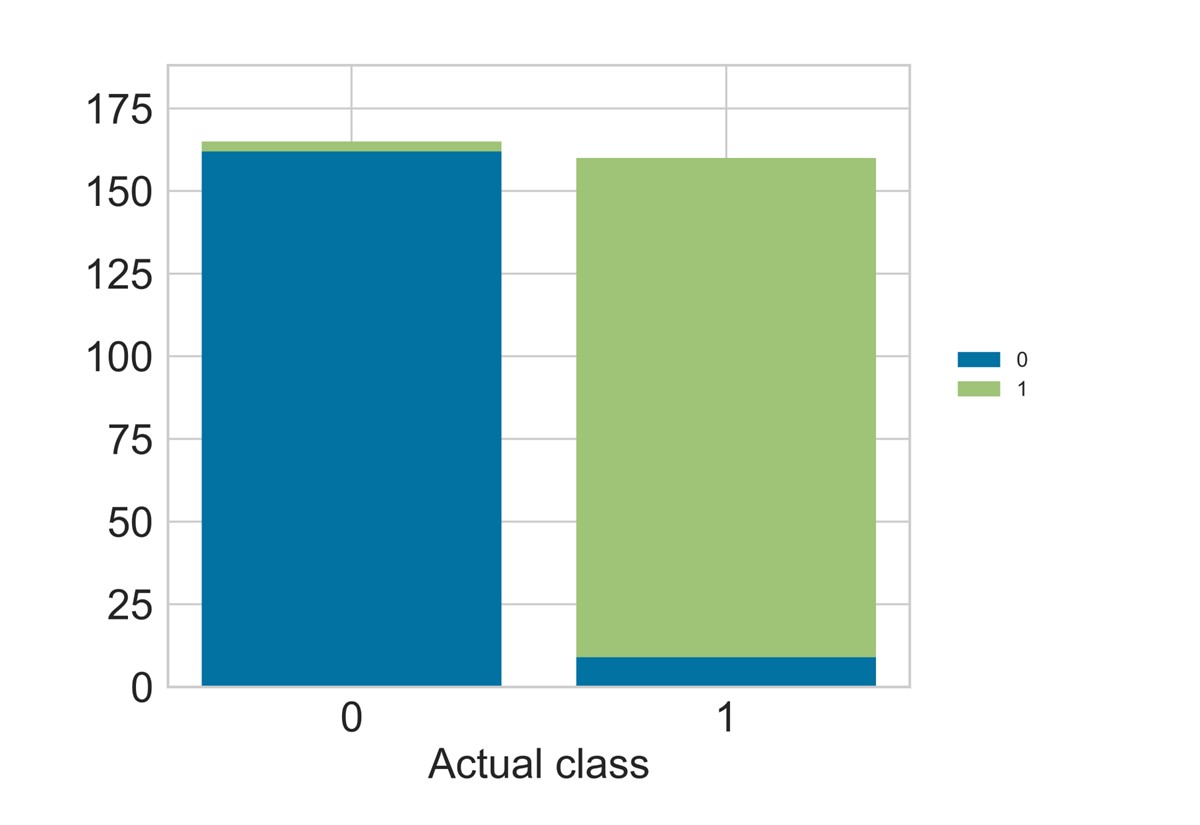


a

b

c

d

Figure S5.

(a) Classification report, (b) Prediction error, (c) Precision recall curve, and (d) Decision boundary plots of the best B-McoL model (Class 0: Correlation <0.8; Class 1: Correlation >= 0.8).


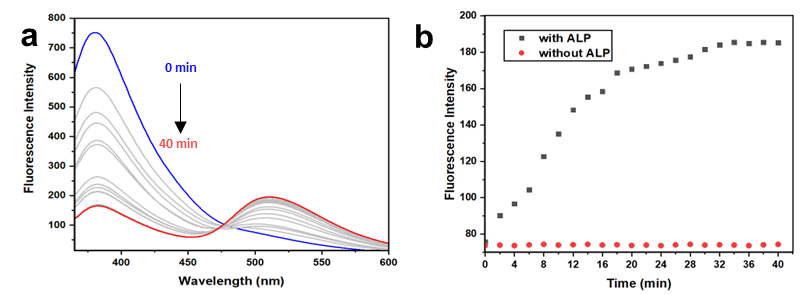


Figure S6.

(a) Fluorescence spectra of different response times of **P1-ALP** to ALP. (b) Fluorescence intensity curves of different response times of **P1-ALP** to ALP (at 510 nm).

Figure S7.

Selectivity and anti-interference of probe **P1-ALP** (1. NaCl; 2. KCl; 3. CaCl_2_; 4. MgCl_2_; 5. FeCl_3_; 6. ZnCl_2_; 7. HSA; 8. FBS; 9. Cys; 10. Gly; 11. Glu; 12. Cys; 13. Hcy; 14. GSH; 15. ppi; C=100 μM).





Figure S8.

Probe **P1-ALP** responds to ALP under different pH conditions.


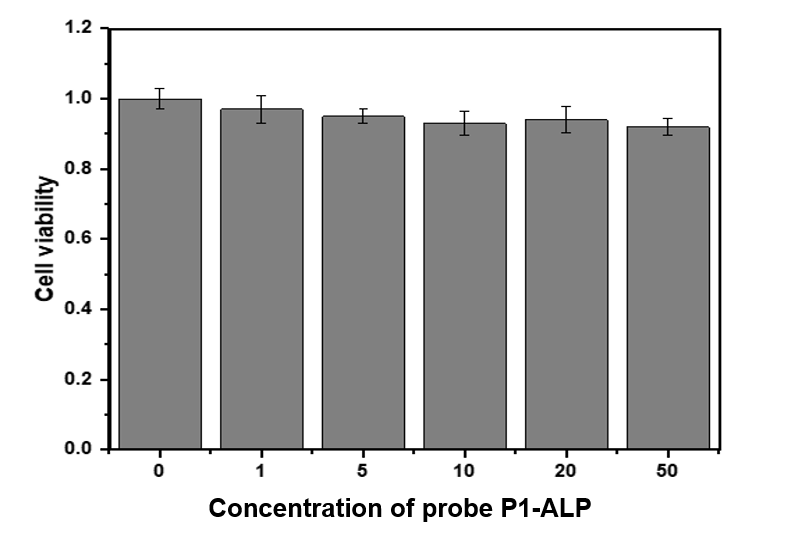


Figure S9.

MTT assay of probe **P1-ALP**.


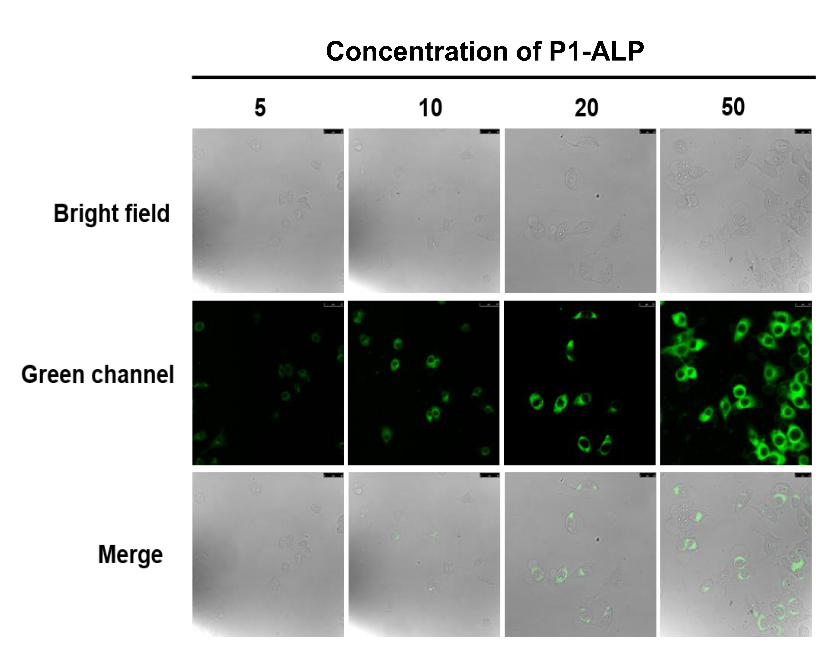


Figure S10.

Fluorescence imaging of probe **P1-ALP** in response to ALP in cell experiment.


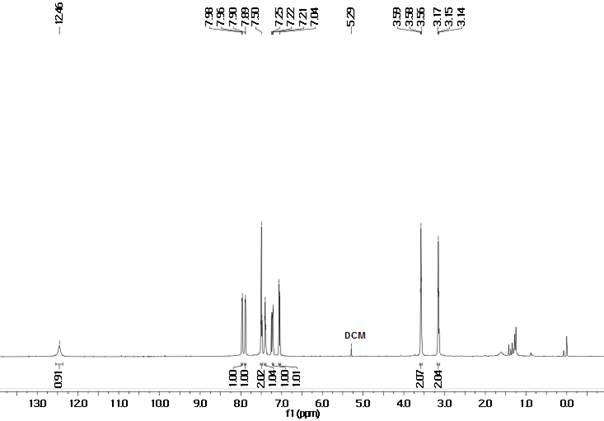


Figure S11.

^1^H-NMR spectrum of compound **1** in CDCl_3_.


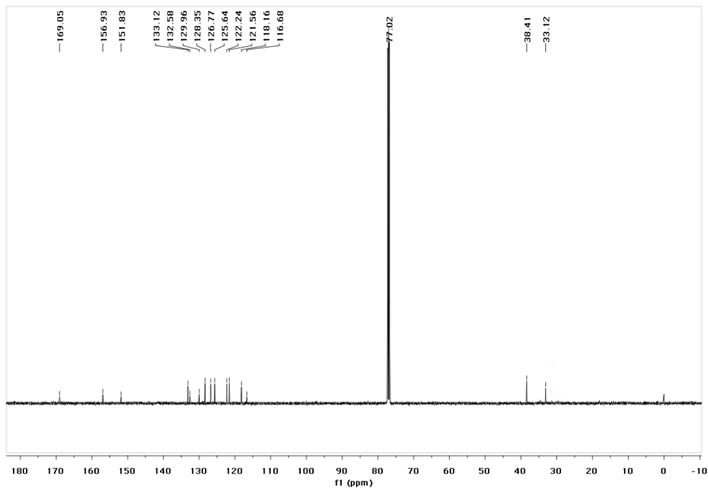


Figure S12.

^13^C-NMR spectrum of compound **1** in CDCl_3_.


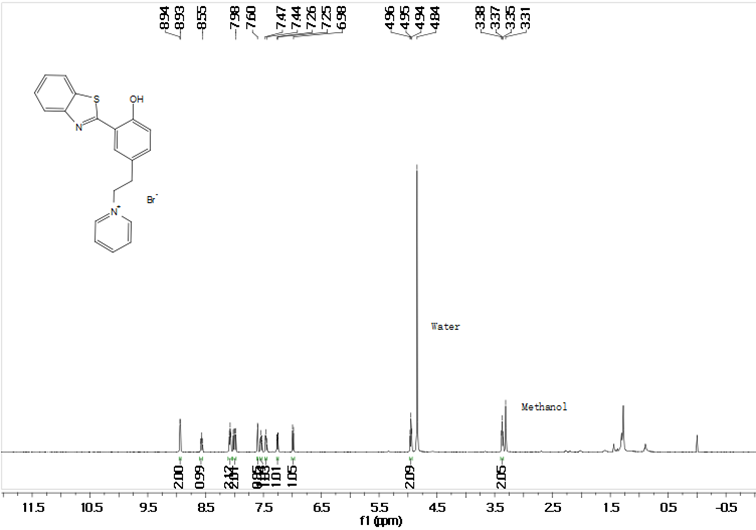


Figure S13.

^13^C-NMR spectrum of **P1** in CDCl_3_.


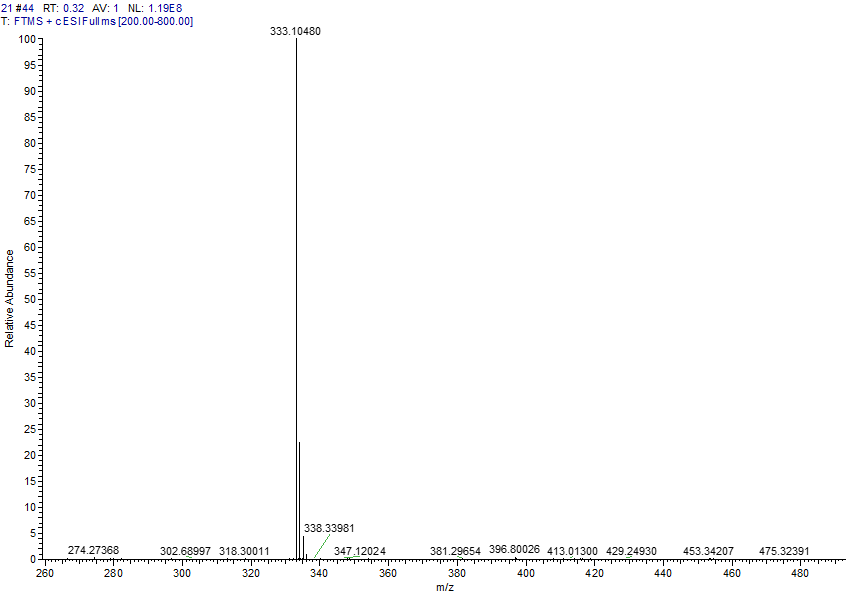


Figure S14.

HR-MS spectrum of compound **P1**.


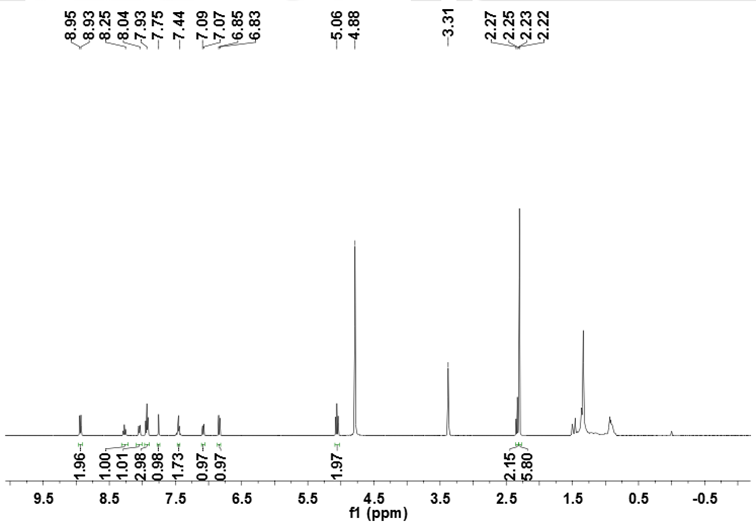


Figure S15.

^1^H-NMR spectrum of **P1** in CD_3_OD.


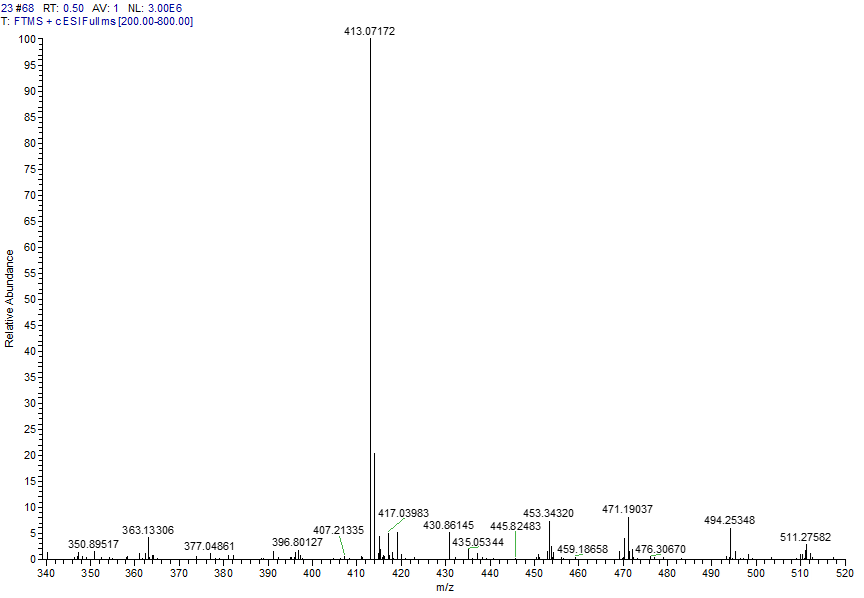


Figure S16.

HR-MS spectrum of probe **P1-ALP**.


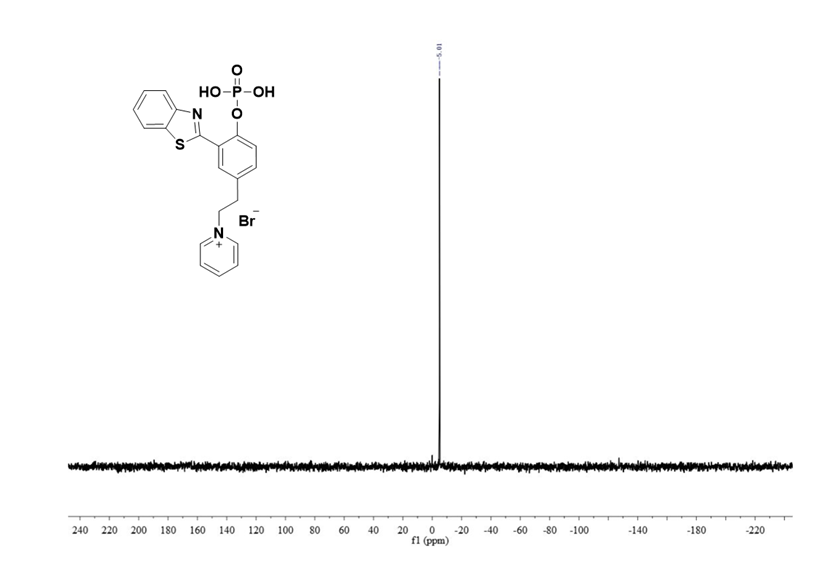


Figure S17.

^31^P-NMR (202 MHz) of probe **P1-ALP**.


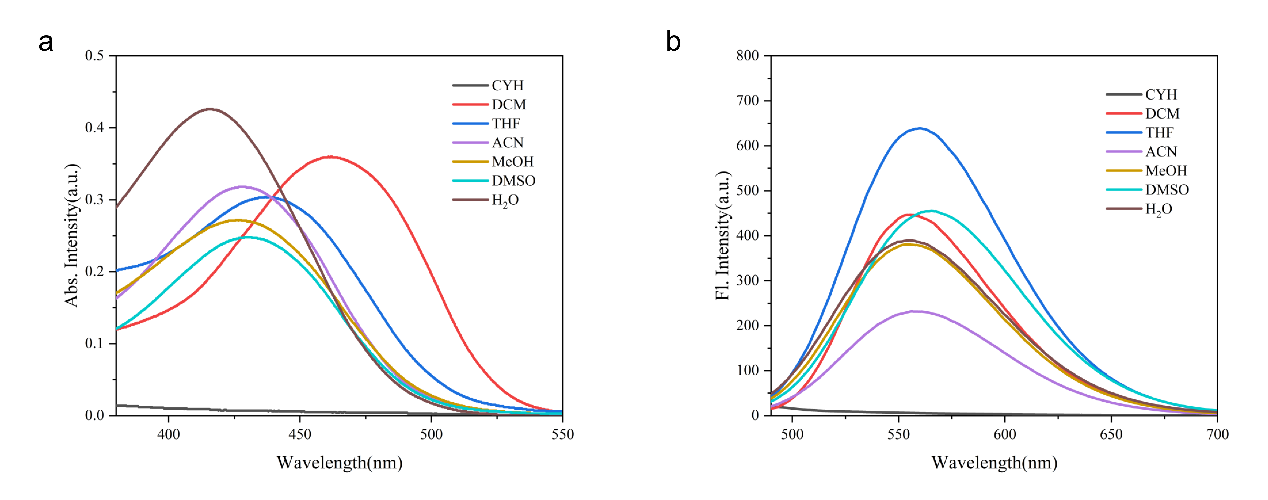


Figure S18.

(a) UV absorption solvent effect spectrum of **P2-VIS**. (b) FL solvent effect spectrum of **P2-VIS**.


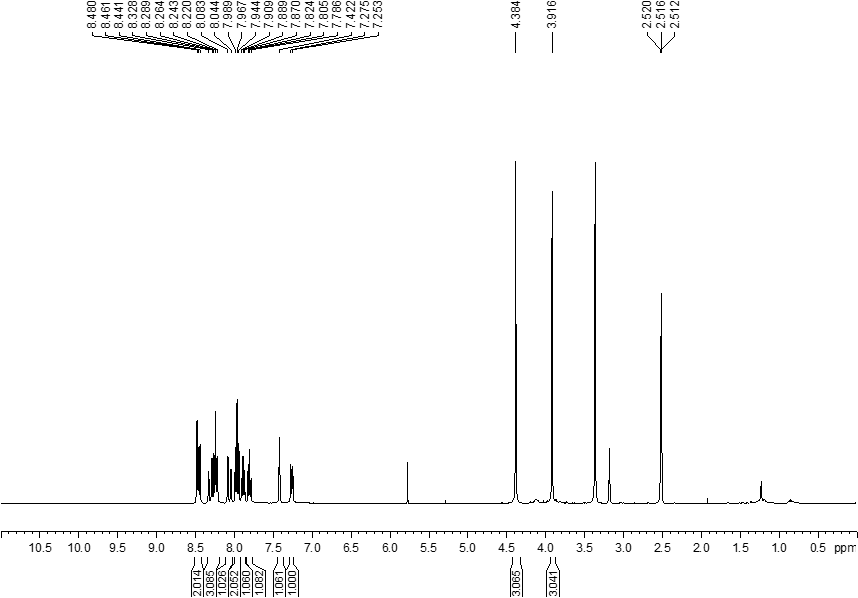


Figure S19.

^1^H-NMR spectrum of probe **P2-VIS** in DMSO-d_6_.


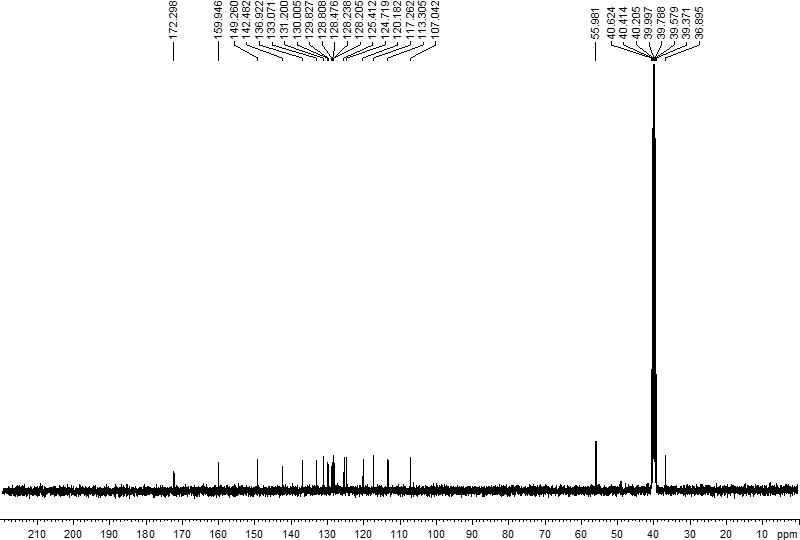


Figure S20.

^13^C-NMR spectrum of probe **P2-VIS** in DMSO-d_6_.


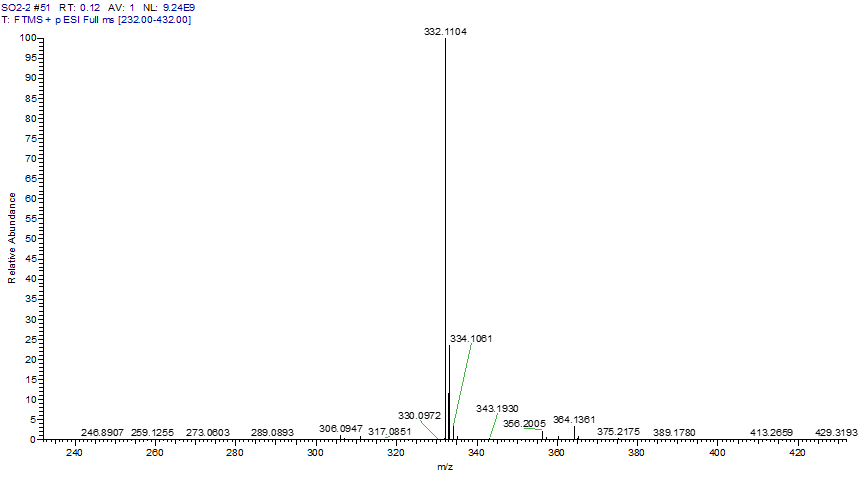


Figure S21.

HR-MS spectrum of probe **P2-VIS**.


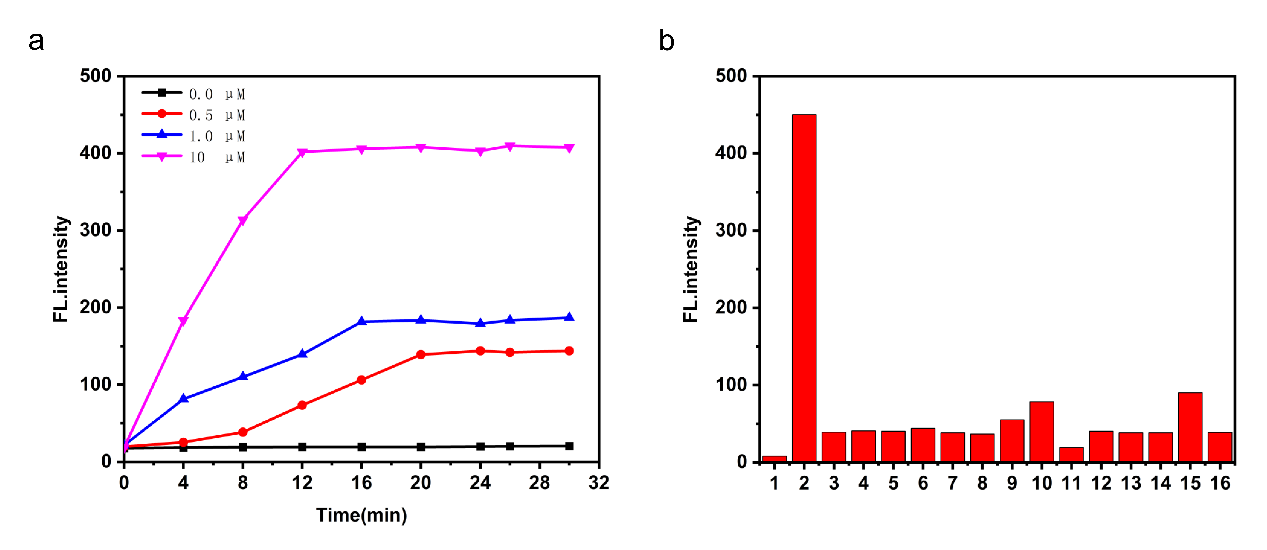


Figure S22.

(a) Response time test of **P3-ROS** to H_2_O_2_ at different concentrations. (b) Response experiments of **P3-ROS** to different compounds. 1. blank; 2.H_2_O_2_; 3.F^-^; 4. Cl^-^; 5. I^-^; 6. AcO^-^; 7. CO_3_^2-^; 8. HSO_4_^-^; 9. PO_4_^3-^; 10. NO_2_^-^; 11.·OH; 12. SO_3_^2-^; 13. ^1^O_2_; 14. O_2_^-^;15. ROO·; 16. NO·.


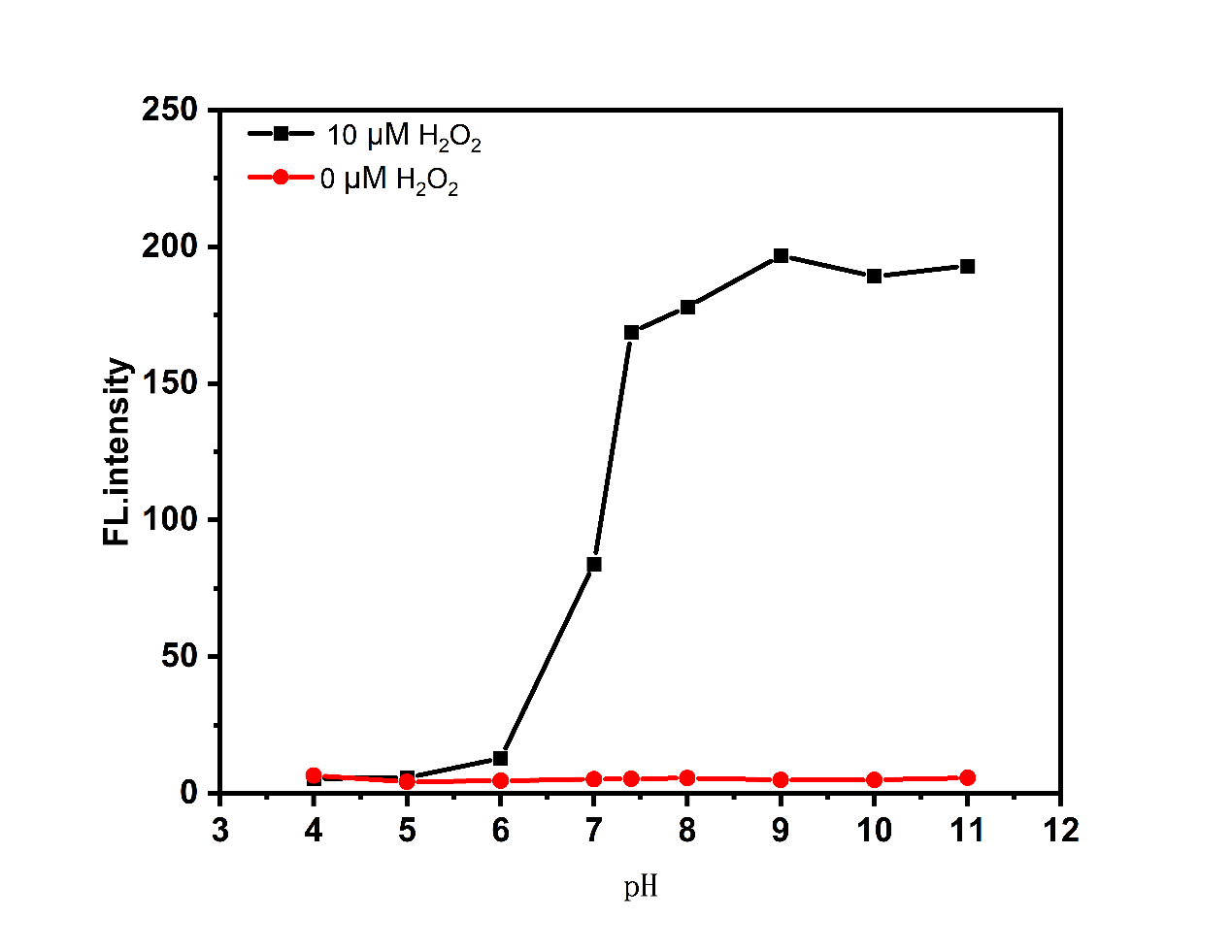


Figure S23.

pH experiments of **P3-ROS** before and after the response to H_2_O_2_.


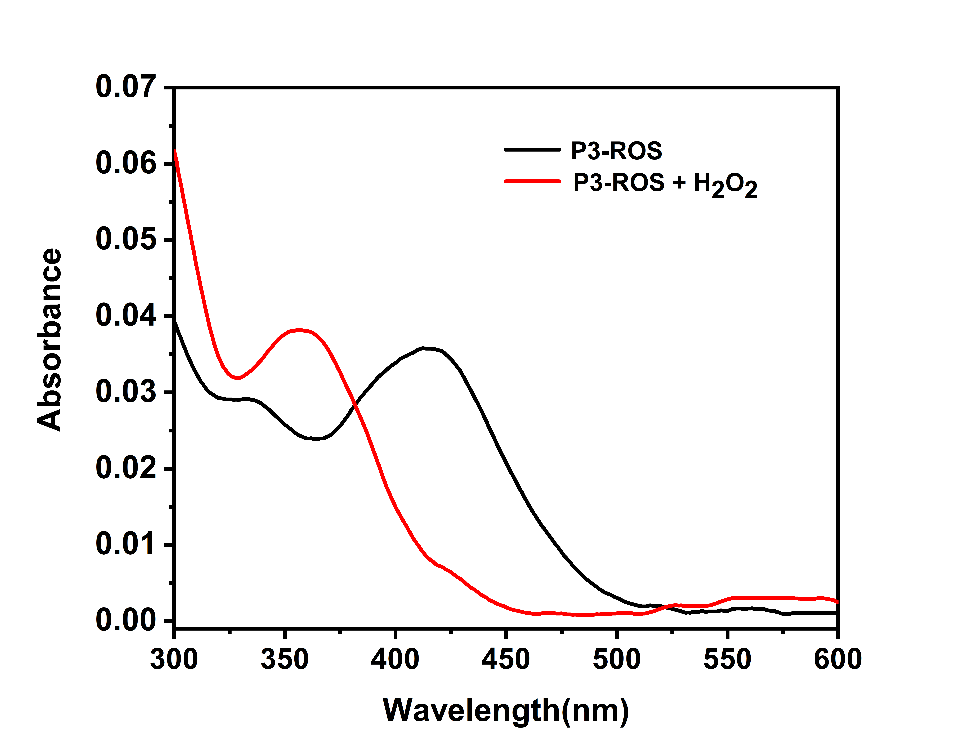


Figure S24.

UV absorption spectra of **P3-ROS** before and after response to H_2_O_2_.


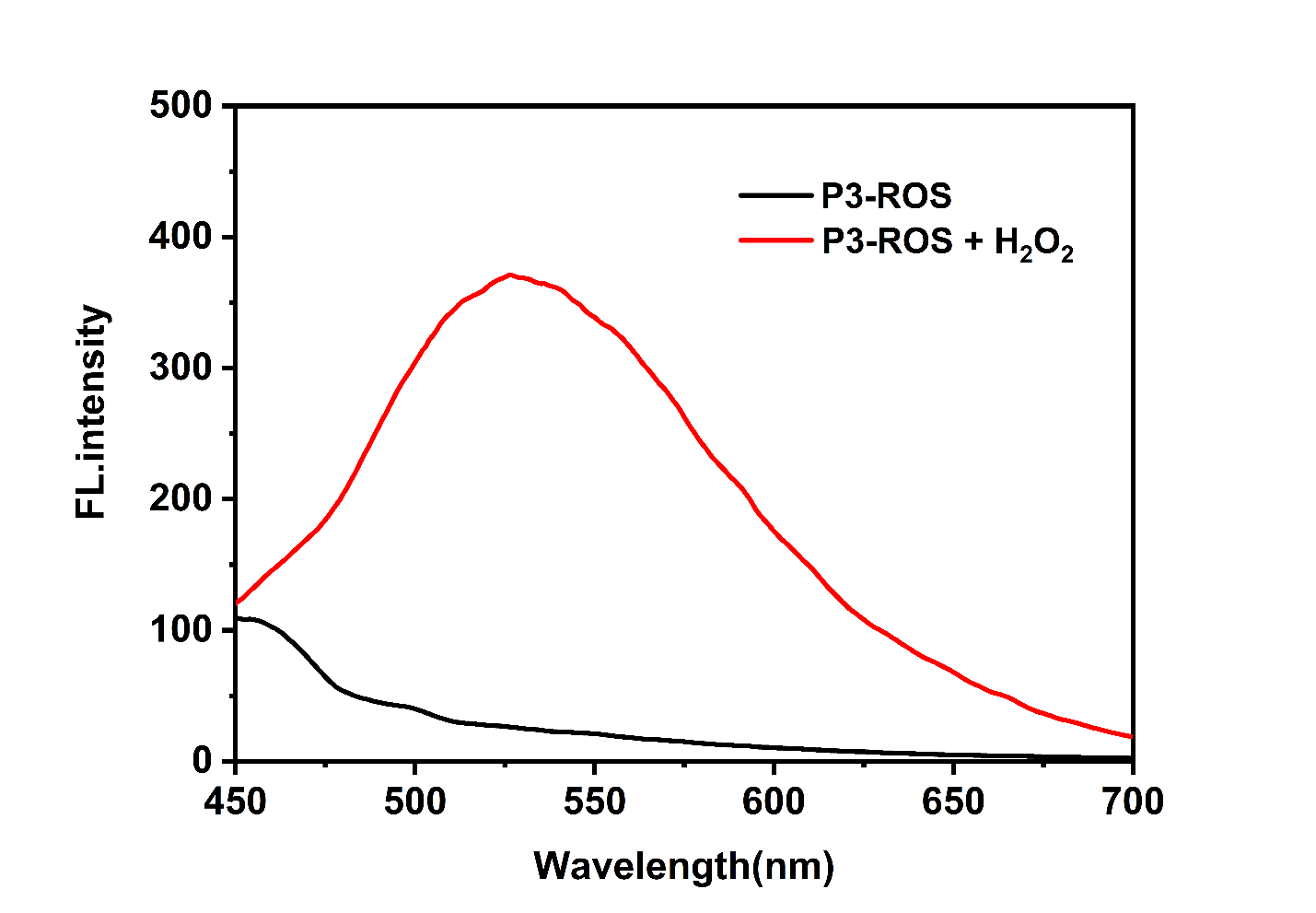


Figure S25.

Fluorescence emission spectra of **P3-ROS** before and after response to H_2_O_2_.


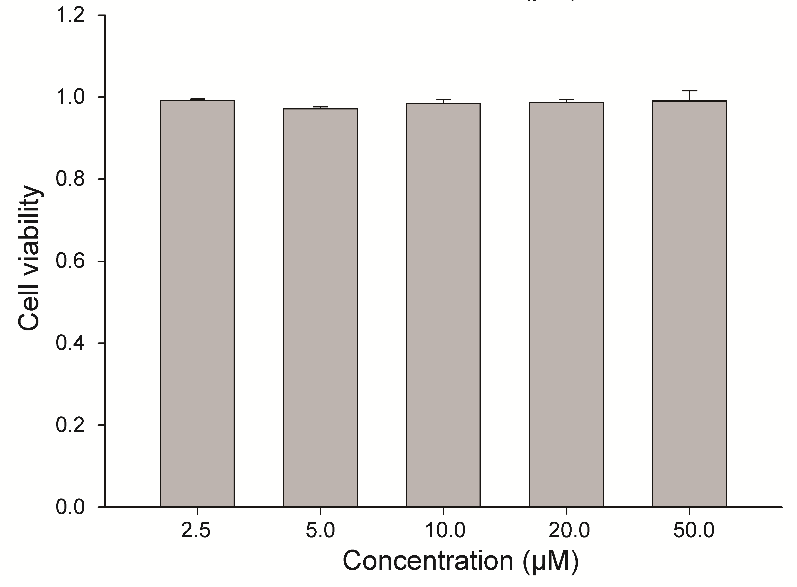


Figure S26.

MTT experiment of probe **P3-ROS**.


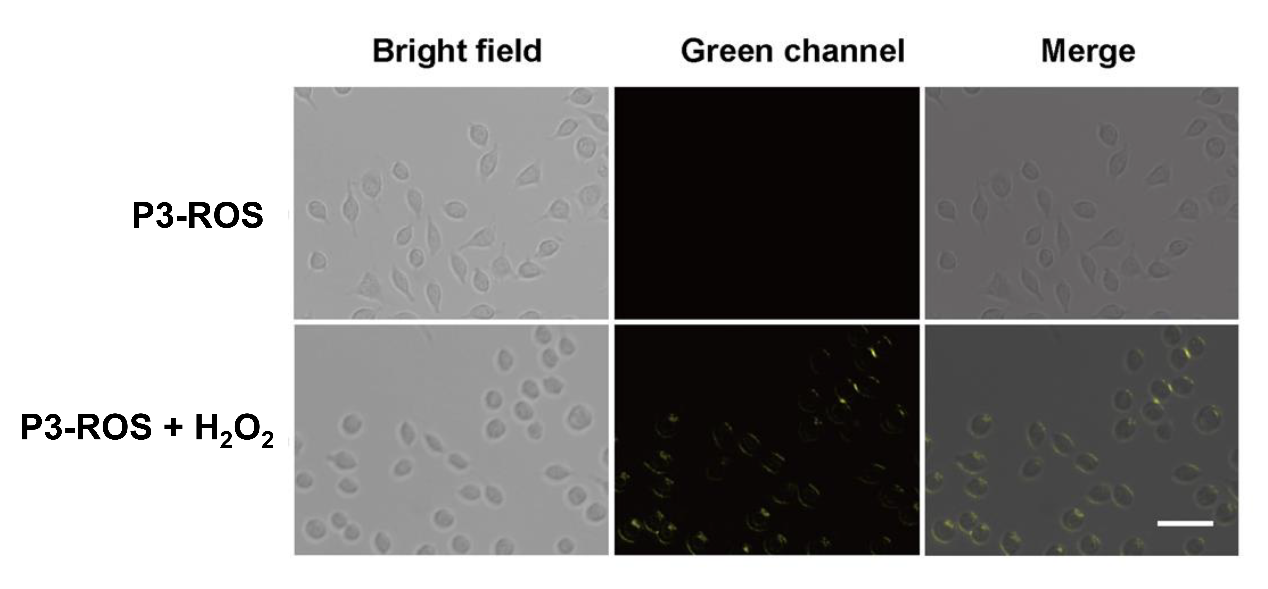


Figure S27.

Fluorescence imaging of probe **P3-ROS** in response to H_2_O_2_ in cell experiment.

Figure S28.

^1^H-NMR spectrum of compound **2** in CDCl_3_.

Figure S29.

^1^H-NMR spectrum of **P3-ROS** in (CD_3_) _2_SO.

Figure S30.

^13^C-NMR spectrum of **P3-ROS** in (CD_3_) _2_SO.


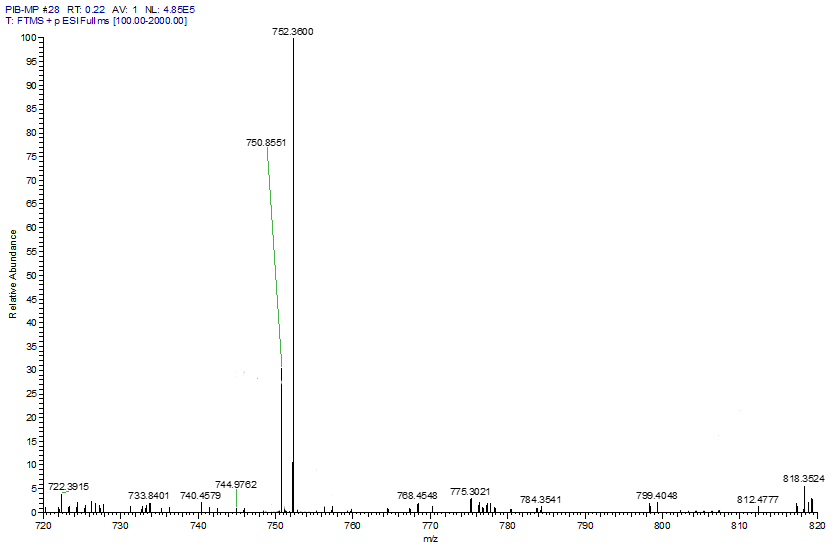


Figure S31.

HR-MS spectrum of probe **P3-ROS**.


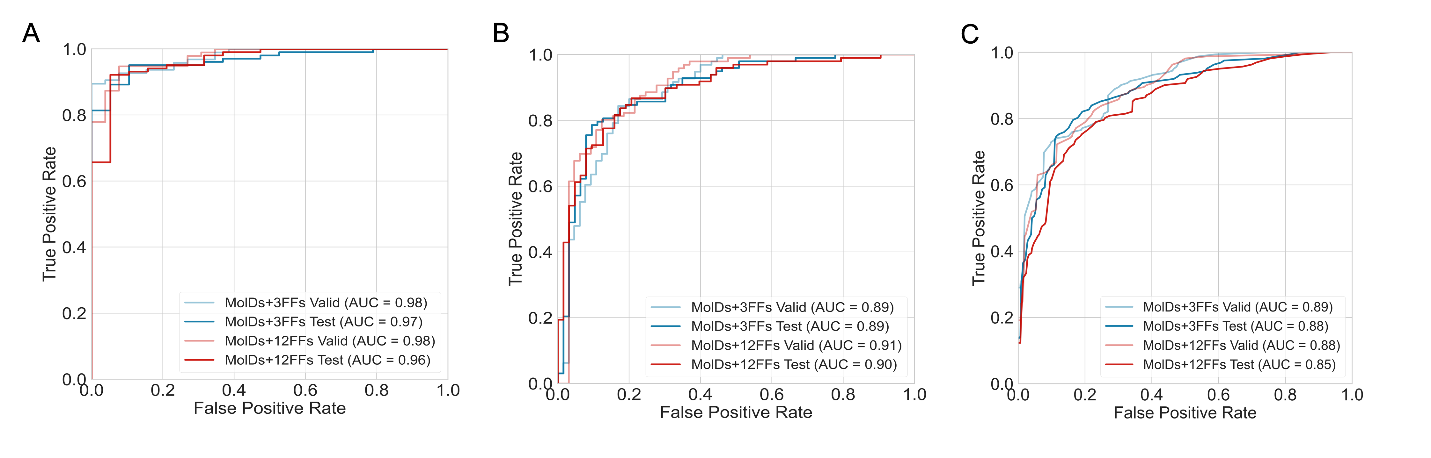


**Figure S32.**

The ROC curves of the models built by MolMapNet. A) B-PvsC, B) B-MvsP, C) M-PvsP.


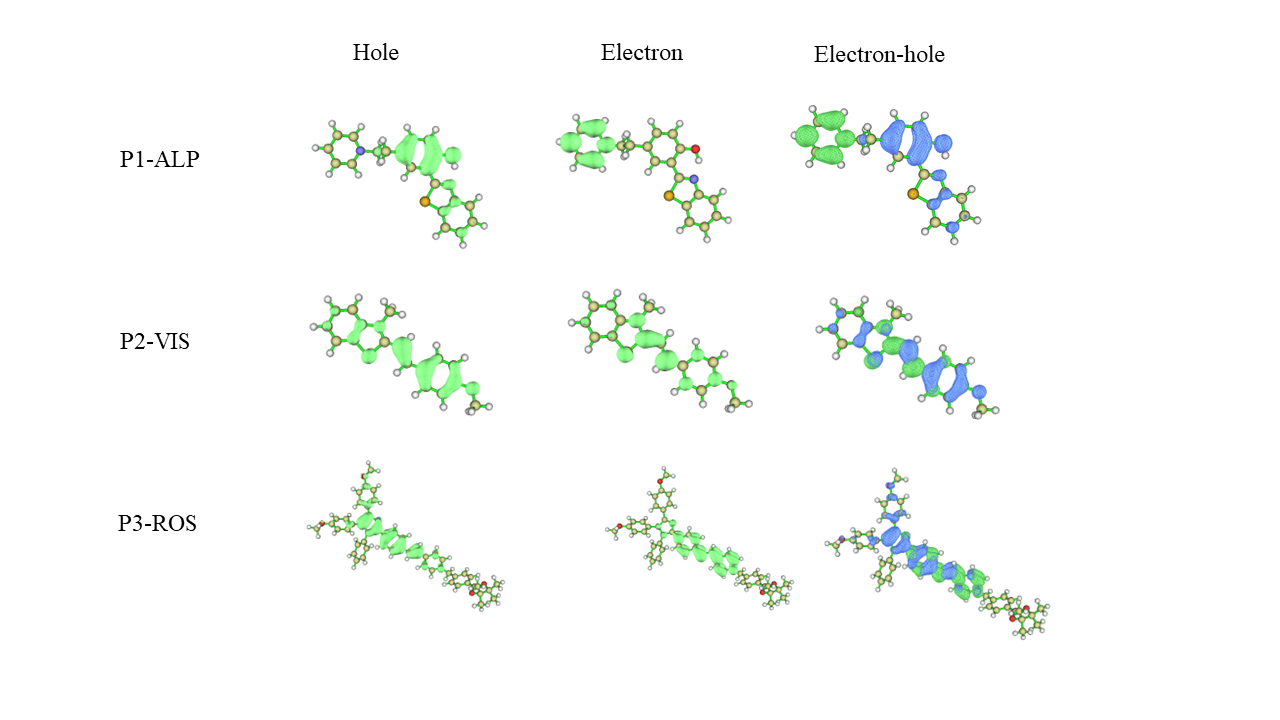


**Figure S33.**

The results of the electron-hole analysis for P1-ALP, P2-VIS and P3-ROS.

1. **Supplementary Tables S1 to S13**

Table S1.

Distribution of the collected data for modeling.

|  | | | |
| --- | --- | --- | --- |
| **Localization** | **Probes *** | **Np-Compounds** | **Sum** |
| Golgi apparatus | 41 (40) | - | 41 |
| Endoplasmic reticulum | 156 (153) | - | 156 |
| Lysosome | 370 (361) | 164 | 534 |
| Cell membrane | 37 (36) | 111 | 148 |
| Mitochondria | 1005 (982) | 236 | 1241 |
| Nucleus | 52 (50) | 103 | 155 |
| Sum | 1661 (1613) | 614 | 2275 |
| * The number in the brackets represents probes after removing duplicates | | | |

Table S2.

The evaluation result of B-PvsC models.

| **Descriptor** | **Algorithm** | **CV** | | | | **Test** | | | |
| --- | --- | --- | --- | --- | --- | --- | --- | --- | --- |
|  |  | **ACC** | **AUC** | **Recall** | **Precision** | **ACC** | **AUC** | **Recall** | **Precision** |
| 2D | DT | 0.889 | 0.821 | 0.930 | 0.933 | 0.898 | 0.862 | 0.927 | 0.941 |
|  | RF | 0.916 | 0.961 | 0.985 | 0.919 | 0.926 | 0.985 | 0.990 | 0.922 |
|  | AdaBoost | 0.904 | 0.941 | 0.957 | 0.927 | 0.898 | 0.907 | 0.958 | 0.915 |
|  | CatBoost | 0.928 | 0.967 | 0.981 | 0.934 | 0.943 | 0.977 | 0.984 | 0.945 |
|  | GBT | 0.920 | 0.963 | 0.972 | 0.932 | 0.926 | 0.953 | 0.974 | 0.935 |
|  | XGBoost | 0.919 | 0.959 | 0.963 | 0.939 | 0.934 | 0.979 | 0.974 | 0.944 |
|  | LightGBM | 0.926 | 0.965 | 0.970 | 0.941 | 0.943 | 0.977 | 0.969 | 0.959 |
|  | ET | 0.908 | 0.948 | 0.967 | 0.923 | 0.902 | 0.948 | 0.969 | 0.911 |
|  | LR | 0.911 | 0.949 | 0.965 | 0.929 | 0.906 | 0.944 | 0.963 | 0.920 |
|  | linearSVM | 0.904 | 0.921 | 0.947 | 0.936 | 0.877 | 0.904 | 0.927 | 0.917 |
| MACCS | DT | 0.863 | 0.779 | 0.914 | 0.917 | 0.877 | 0.806 | 0.932 | 0.913 |
|  | RF | 0.913 | 0.944 | 0.972 | 0.915 | 0.910 | 0.968 | 0.984 | 0.908 |
|  | AdaBoost | 0.899 | 0.936 | 0.960 | 0.921 | 0.885 | 0.949 | 0.958 | 0.901 |
|  | CatBoost | 0.918 | 0.960 | 0.981 | 0.923 | 0.939 | 0.976 | 0.979 | 0.944 |
|  | GBT | 0.907 | 0.950 | 0.970 | 0.920 | 0.934 | 0.973 | 0.990 | 0.931 |
|  | XGBoost | 0.918 | 0.958 | 0.975 | 0.928 | 0.934 | 0.978 | 0.979 | 0.940 |
|  | LightGBM | 0.919 | 0.964 | 0.970 | 0.933 | 0.955 | 0.987 | 0.984 | 0.959 |
|  | ET | 0.898 | 0.926 | 0.967 | 0.913 | 0.889 | 0.943 | 0.963 | 0.902 |
|  | LR | 0.902 | 0.923 | 0.953 | 0.929 | 0.902 | 0.945 | 0.948 | 0.928 |
|  | linearSVM | 0.893 | 0.904 | 0.953 | 0.919 | 0.873 | 0.924 | 0.932 | 0.908 |
| ECFP4 | DT | 0.863 | 0.763 | 0.924 | 0.909 | 0.906 | 0.885 | 0.921 | 0.957 |
|  | RF | 0.918 | 0.960 | 0.981 | 0.923 | 0.951 | 0.992 | 0.995 | 0.945 |
|  | AdaBoost | 0.913 | 0.903 | 0.960 | 0.935 | 0.918 | 0.946 | 0.974 | 0.925 |
|  | CatBoost | 0.917 | 0.966 | 0.980 | 0.923 | 0.955 | 0.990 | 0.990 | 0.955 |
|  | GBT | 0.910 | 0.952 | 0.979 | 0.916 | 0.947 | 0.977 | 0.979 | 0.954 |
|  | XGBoost | 0.931 | 0.962 | 0.968 | 0.948 | 0.947 | 0.985 | 0.974 | 0.959 |
|  | LightGBM | 0.923 | 0.961 | 0.965 | 0.942 | 0.963 | 0.993 | 0.979 | 0.974 |
|  | ET | 0.909 | 0.929 | 0.967 | 0.924 | 0.914 | 0.963 | 0.974 | 0.921 |
|  | LR | 0.933 | 0.974 | 0.974 | 0.946 | 0.955 | 0.981 | 0.990 | 0.957 |
|  | linearSVM | 0.915 | 0.951 | 0.949 | 0.946 | 0.939 | 0.974 | 0.948 | 0.973 |
| AtomPair | DT | 0.862 | 0.770 | 0.918 | 0.914 | 0.852 | 0.752 | 0.932 | 0.886 |
|  | RF | 0.907 | 0.953 | 0.975 | 0.906 | 0.898 | 0.963 | 0.995 | 0.888 |
|  | AdaBoost | 0.886 | 0.883 | 0.948 | 0.915 | 0.885 | 0.917 | 0.948 | 0.910 |
|  | CatBoost | 0.896 | 0.945 | 0.967 | 0.911 | 0.914 | 0.966 | 0.990 | 0.909 |
|  | GBT | 0.897 | 0.930 | 0.962 | 0.916 | 0.914 | 0.958 | 0.995 | 0.905 |
|  | XGBoost | 0.906 | 0.943 | 0.965 | 0.923 | 0.939 | 0.970 | 0.984 | 0.940 |
|  | LightGBM | 0.907 | 0.944 | 0.967 | 0.922 | 0.959 | 0.978 | 1.000 | 0.950 |
|  | ET | 0.887 | 0.907 | 0.957 | 0.909 | 0.885 | 0.915 | 0.963 | 0.898 |
|  | LR | 0.906 | 0.939 | 0.954 | 0.931 | 0.939 | 0.959 | 0.963 | 0.958 |
|  | linearSVM | 0.890 | 0.915 | 0.936 | 0.930 | 0.910 | 0.954 | 0.937 | 0.947 |
| CDK | DT | 0.860 | 0.802 | 0.896 | 0.929 | 0.889 | 0.838 | 0.932 | 0.927 |
|  | RF | 0.920 | 0.957 | 0.977 | 0.927 | 0.955 | 0.990 | 0.990 | 0.955 |
|  | AdaBoost | 0.927 | 0.950 | 0.967 | 0.945 | 0.922 | 0.945 | 0.948 | 0.953 |
|  | CatBoost | 0.931 | 0.961 | 0.985 | 0.934 | 0.934 | 0.984 | 0.995 | 0.926 |
|  | GBT | 0.935 | 0.960 | 0.976 | 0.946 | 0.922 | 0.968 | 0.964 | 0.940 |
|  | XGBoost | 0.935 | 0.961 | 0.973 | 0.949 | 0.955 | 0.971 | 0.984 | 0.959 |
|  | LightGBM | 0.934 | 0.962 | 0.977 | 0.944 | 0.951 | 0.977 | 0.969 | 0.969 |
|  | ET | 0.901 | 0.934 | 0.948 | 0.932 | 0.951 | 0.979 | 0.990 | 0.950 |
|  | LR | 0.928 | 0.961 | 0.966 | 0.947 | 0.955 | 0.981 | 0.984 | 0.959 |
|  | linearSVM | 0.914 | 0.949 | 0.952 | 0.942 | 0.939 | 0.977 | 0.963 | 0.958 |
| Pubchem | DT | 0.882 | 0.802 | 0.930 | 0.925 | 0.889 | 0.826 | 0.937 | 0.923 |
|  | RF | 0.928 | 0.963 | 0.973 | 0.932 | 0.922 | 0.980 | 0.974 | 0.930 |
|  | AdaBoost | 0.908 | 0.941 | 0.954 | 0.934 | 0.881 | 0.888 | 0.932 | 0.918 |
|  | CatBoost | 0.932 | 0.969 | 0.981 | 0.938 | 0.943 | 0.981 | 0.979 | 0.949 |
|  | GBT | 0.913 | 0.962 | 0.968 | 0.928 | 0.893 | 0.965 | 0.969 | 0.902 |
|  | XGBoost | 0.918 | 0.963 | 0.958 | 0.942 | 0.963 | 0.988 | 0.990 | 0.964 |
|  | LightGBM | 0.933 | 0.969 | 0.973 | 0.947 | 0.947 | 0.983 | 0.984 | 0.949 |
|  | ET | 0.918 | 0.944 | 0.962 | 0.939 | 0.930 | 0.950 | 0.974 | 0.939 |
|  | LR | 0.916 | 0.961 | 0.958 | 0.939 | 0.926 | 0.970 | 0.953 | 0.953 |
|  | linearSVM | 0.906 | 0.936 | 0.933 | 0.950 | 0.910 | 0.958 | 0.932 | 0.952 |

Table S3.

The evaluation result of B-MvsP models.

| **Descriptor** | **Algorithm** | **CV** | | | | **Test** | | | |
| --- | --- | --- | --- | --- | --- | --- | --- | --- | --- |
|  |  | **ACC** | **AUC** | **Recall** | **Precision** | **ACC** | **AUC** | **Recall** | **Precision** |
| 2D | DT | 0.778 | 0.764 | 0.827 | 0.816 | 0.765 | 0.762 | 0.788 | 0.797 |
|  | RF | 0.834 | 0.910 | 0.886 | 0.862 | 0.846 | 0.902 | 0.891 | 0.845 |
|  | AdaBoost | 0.819 | 0.876 | 0.866 | 0.846 | 0.815 | 0.889 | 0.853 | 0.826 |
|  | CatBoost | 0.842 | 0.916 | 0.887 | 0.862 | 0.846 | 0.904 | 0.870 | 0.860 |
|  | GBT | 0.846 | 0.910 | 0.881 | 0.871 | 0.824 | 0.895 | 0.870 | 0.829 |
|  | XGBoost | 0.848 | 0.911 | 0.891 | 0.867 | 0.812 | 0.895 | 0.853 | 0.822 |
|  | LightGBM | 0.838 | 0.910 | 0.885 | 0.858 | 0.824 | 0.897 | 0.864 | 0.832 |
|  | ET | 0.832 | 0.881 | 0.853 | 0.873 | 0.821 | 0.876 | 0.826 | 0.854 |
|  | LR | 0.812 | 0.879 | 0.856 | 0.838 | 0.815 | 0.873 | 0.873 | 0.831 |
|  | linearSVM | 0.831 | 0.878 | 0.875 | 0.855 | 0.790 | 0.846 | 0.842 | 0.799 |
| MACCS | DT | 0.796 | 0.784 | 0.841 | 0.831 | 0.759 | 0.758 | 0.804 | 0.779 |
|  | RF | 0.832 | 0.901 | 0.901 | 0.839 | 0.806 | 0.887 | 0.902 | 0.787 |
|  | AdaBoost | 0.809 | 0.872 | 0.867 | 0.831 | 0.818 | 0.875 | 0.870 | 0.821 |
|  | CatBoost | 0.845 | 0.914 | 0.901 | 0.855 | 0.818 | 0.904 | 0.897 | 0.805 |
|  | GBT | 0.831 | 0.904 | 0.890 | 0.844 | 0.815 | 0.894 | 0.891 | 0.804 |
|  | XGBoost | 0.835 | 0.897 | 0.881 | 0.856 | 0.821 | 0.906 | 0.870 | 0.825 |
|  | LightGBM | 0.858 | 0.916 | 0.908 | 0.865 | 0.843 | 0.902 | 0.869 | 0.874 |
|  | ET | 0.805 | 0.866 | 0.858 | 0.831 | 0.790 | 0.863 | 0.853 | 0.793 |
|  | LR | 0.814 | 0.873 | 0.870 | 0.836 | 0.815 | 0.870 | 0.870 | 0.816 |
|  | linearSVM | 0.792 | 0.844 | 0.847 | 0.822 | 0.802 | 0.861 | 0.848 | 0.813 |
| ECFP4 | DT | 0.761 | 0.739 | 0.830 | 0.792 | 0.716 | 0.708 | 0.777 | 0.737 |
|  | RF | 0.825 | 0.897 | 0.917 | 0.811 | 0.809 | 0.872 | 0.913 | 0.785 |
|  | AdaBoost | 0.808 | 0.859 | 0.877 | 0.824 | 0.790 | 0.880 | 0.864 | 0.787 |
|  | CatBoost | 0.832 | 0.897 | 0.914 | 0.832 | 0.818 | 0.897 | 0.897 | 0.805 |
|  | GBT | 0.815 | 0.877 | 0.892 | 0.824 | 0.809 | 0.885 | 0.891 | 0.796 |
|  | XGBoost | 0.835 | 0.898 | 0.897 | 0.845 | 0.821 | 0.899 | 0.870 | 0.825 |
|  | LightGBM | 0.827 | 0.895 | 0.894 | 0.838 | 0.821 | 0.900 | 0.880 | 0.818 |
|  | ET | 0.793 | 0.852 | 0.855 | 0.818 | 0.775 | 0.866 | 0.848 | 0.776 |
|  | LR | 0.809 | 0.867 | 0.865 | 0.833 | 0.818 | 0.892 | 0.853 | 0.831 |
|  | linearSVM | 0.771 | 0.820 | 0.811 | 0.818 | 0.765 | 0.822 | 0.826 | 0.776 |
| AtomPair | DT | 0.707 | 0.694 | 0.753 | 0.769 | 0.676 | 0.673 | 0.728 | 0.709 |
|  | RF | 0.788 | 0.851 | 0.899 | 0.794 | 0.753 | 0.841 | 0.859 | 0.745 |
|  | AdaBoost | 0.744 | 0.795 | 0.823 | 0.775 | 0.750 | 0.813 | 0.832 | 0.754 |
|  | CatBoost | 0.795 | 0.863 | 0.890 | 0.801 | 0.809 | 0.872 | 0.902 | 0.790 |
|  | GBT | 0.775 | 0.849 | 0.870 | 0.788 | 0.787 | 0.853 | 0.880 | 0.775 |
|  | XGBoost | 0.792 | 0.855 | 0.867 | 0.810 | 0.781 | 0.856 | 0.848 | 0.784 |
|  | LightGBM | 0.798 | 0.854 | 0.875 | 0.812 | 0.790 | 0.861 | 0.870 | 0.784 |
|  | ET | 0.735 | 0.786 | 0.812 | 0.771 | 0.698 | 0.767 | 0.728 | 0.736 |
|  | LR | 0.730 | 0.791 | 0.791 | 0.775 | 0.744 | 0.775 | 0.799 | 0.762 |
|  | linearSVM | 0.715 | 0.768 | 0.762 | 0.773 | 0.670 | 0.747 | 0.701 | 0.713 |
| CDK | DT | 0.727 | 0.720 | 0.754 | 0.796 | 0.769 | 0.763 | 0.804 | 0.791 |
|  | RF | 0.804 | 0.868 | 0.886 | 0.812 | 0.790 | 0.852 | 0.848 | 0.796 |
|  | AdaBoost | 0.766 | 0.829 | 0.845 | 0.791 | 0.765 | 0.829 | 0.842 | 0.767 |
|  | CatBoost | 0.812 | 0.880 | 0.895 | 0.818 | 0.802 | 0.875 | 0.897 | 0.786 |
|  | GBT | 0.798 | 0.867 | 0.867 | 0.816 | 0.802 | 0.865 | 0.886 | 0.791 |
|  | XGBoost | 0.798 | 0.876 | 0.863 | 0.820 | 0.781 | 0.869 | 0.853 | 0.781 |
|  | LightGBM | 0.802 | 0.873 | 0.871 | 0.821 | 0.784 | 0.866 | 0.870 | 0.777 |
|  | ET | 0.781 | 0.837 | 0.822 | 0.823 | 0.759 | 0.834 | 0.804 | 0.779 |
|  | LR | 0.771 | 0.837 | 0.828 | 0.805 | 0.781 | 0.845 | 0.837 | 0.790 |
|  | linearSVM | 0.743 | 0.796 | 0.778 | 0.799 | 0.762 | 0.801 | 0.804 | 0.783 |
| Pubchem | DT | 0.761 | 0.746 | 0.812 | 0.802 | 0.756 | 0.749 | 0.804 | 0.775 |
|  | RF | 0.805 | 0.877 | 0.886 | 0.823 | 0.802 | 0.870 | 0.875 | 0.797 |
|  | AdaBoost | 0.785 | 0.837 | 0.856 | 0.807 | 0.796 | 0.870 | 0.853 | 0.801 |
|  | CatBoost | 0.822 | 0.883 | 0.917 | 0.816 | 0.793 | 0.880 | 0.870 | 0.788 |
|  | GBT | 0.806 | 0.869 | 0.897 | 0.810 | 0.799 | 0.865 | 0.880 | 0.790 |
|  | XGBoost | 0.804 | 0.873 | 0.867 | 0.824 | 0.781 | 0.871 | 0.837 | 0.790 |
|  | LightGBM | 0.818 | 0.874 | 0.878 | 0.835 | 0.778 | 0.880 | 0.848 | 0.780 |
|  | ET | 0.795 | 0.842 | 0.858 | 0.818 | 0.796 | 0.848 | 0.853 | 0.801 |
|  | LR | 0.792 | 0.852 | 0.861 | 0.813 | 0.809 | 0.879 | 0.842 | 0.824 |
|  | linearSVM | 0.758 | 0.798 | 0.805 | 0.804 | 0.756 | 0.800 | 0.815 | 0.769 |

Table S4.

The evaluation result of M-PvsP models.

| **Descriptor** | **Algorithm** | **CV** | | | | **Test** | | | |
| --- | --- | --- | --- | --- | --- | --- | --- | --- | --- |
|  |  | **ACC** | **AUC** | **Recall** | **Precision** | **ACC** | **AUC** | **Recall** | **Precision** |
| 2D | DT | 0.683 | 0.734 | 0.408 | 0.687 | 0.683 | 0.756 | 0.413 | 0.708 |
|  | RF | 0.771 | 0.900 | 0.404 | 0.738 | 0.812 | 0.928 | 0.457 | 0.801 |
|  | AdaBoost | 0.671 | 0.682 | 0.360 | 0.651 | 0.702 | 0.726 | 0.451 | 0.708 |
|  | CatBoost | 0.746 | 0.896 | 0.746 | 0.706 | 0.785 | 0.909 | 0.785 | 0.742 |
|  | GBT | 0.766 | 0.897 | 0.446 | 0.739 | 0.815 | 0.927 | 0.483 | 0.796 |
|  | XGBoost | 0.780 | 0.910 | 0.780 | 0.748 | 0.788 | 0.912 | 0.788 | 0.771 |
|  | LightGBM | 0.773 | 0.903 | 0.436 | 0.750 | 0.803 | 0.935 | 0.482 | 0.785 |
|  | ET | 0.780 | 0.898 | 0.426 | 0.754 | 0.797 | 0.916 | 0.450 | 0.777 |
|  | LR | 0.747 | 0.874 | 0.747 | 0.717 | 0.775 | 0.882 | 0.775 | 0.747 |
|  | linearSVM | 0.702 | 0.845 | 0.702 | 0.702 | 0.711 | 0.833 | 0.711 | 0.706 |
| MACCS | DT | 0.697 | 0.756 | 0.423 | 0.704 | 0.726 | 0.796 | 0.546 | 0.754 |
|  | RF | 0.776 | 0.900 | 0.416 | 0.760 | 0.769 | 0.910 | 0.430 | 0.752 |
|  | AdaBoost | 0.573 | 0.661 | 0.319 | 0.627 | 0.665 | 0.733 | 0.343 | 0.673 |
|  | CatBoost | 0.766 | 0.894 | 0.766 | 0.736 | 0.766 | 0.896 | 0.766 | 0.721 |
|  | GBT | 0.768 | 0.896 | 0.447 | 0.741 | 0.806 | 0.921 | 0.518 | 0.798 |
|  | XGBoost | 0.774 | 0.902 | 0.774 | 0.752 | 0.772 | 0.897 | 0.772 | 0.747 |
|  | LightGBM | 0.783 | 0.904 | 0.783 | 0.757 | 0.809 | 0.932 | 0.809 | 0.794 |
|  | ET | 0.782 | 0.899 | 0.455 | 0.763 | 0.772 | 0.906 | 0.465 | 0.764 |
|  | LR | 0.733 | 0.869 | 0.398 | 0.707 | 0.769 | 0.896 | 0.426 | 0.745 |
|  | linearSVM | 0.696 | 0.853 | 0.696 | 0.708 | 0.698 | 0.877 | 0.698 | 0.708 |
| ECFP4 | DT | 0.683 | 0.734 | 0.408 | 0.687 | 0.683 | 0.756 | 0.413 | 0.708 |
|  | RF | 0.771 | 0.900 | 0.404 | 0.738 | 0.812 | 0.928 | 0.457 | 0.801 |
|  | AdaBoost | 0.671 | 0.682 | 0.360 | 0.651 | 0.702 | 0.726 | 0.451 | 0.708 |
|  | CatBoost | 0.756 | 0.871 | 0.756 | 0.731 | 0.769 | 0.884 | 0.769 | 0.771 |
|  | GBT | 0.766 | 0.897 | 0.446 | 0.739 | 0.815 | 0.927 | 0.483 | 0.796 |
|  | XGBoost | 0.779 | 0.896 | 0.779 | 0.758 | 0.772 | 0.890 | 0.772 | 0.768 |
|  | LightGBM | 0.773 | 0.903 | 0.436 | 0.750 | 0.803 | 0.935 | 0.482 | 0.785 |
|  | ET | 0.780 | 0.898 | 0.426 | 0.754 | 0.797 | 0.916 | 0.450 | 0.777 |
|  | LR | 0.717 | 0.855 | 0.717 | 0.733 | 0.732 | 0.851 | 0.732 | 0.738 |
|  | linearSVM | 0.726 | 0.850 | 0.726 | 0.721 | 0.717 | 0.837 | 0.717 | 0.703 |
| AtomPair | DT | 0.592 | 0.670 | 0.308 | 0.607 | 0.612 | 0.685 | 0.416 | 0.638 |
|  | RF | 0.727 | 0.842 | 0.300 | 0.693 | 0.720 | 0.864 | 0.346 | 0.714 |
|  | AdaBoost | 0.568 | 0.605 | 0.300 | 0.585 | 0.643 | 0.636 | 0.326 | 0.619 |
|  | CatBoost | 0.717 | 0.819 | 0.717 | 0.685 | 0.708 | 0.840 | 0.708 | 0.660 |
|  | GBT | 0.722 | 0.842 | 0.356 | 0.678 | 0.708 | 0.864 | 0.431 | 0.694 |
|  | XGBoost | 0.747 | 0.851 | 0.747 | 0.718 | 0.729 | 0.861 | 0.729 | 0.709 |
|  | LightGBM | 0.739 | 0.849 | 0.376 | 0.703 | 0.757 | 0.881 | 0.492 | 0.755 |
|  | ET | 0.739 | 0.846 | 0.319 | 0.710 | 0.735 | 0.868 | 0.390 | 0.730 |
|  | LR | 0.708 | 0.825 | 0.378 | 0.678 | 0.692 | 0.822 | 0.465 | 0.676 |
|  | linearSVM | 0.699 | 0.811 | 0.699 | 0.677 | 0.689 | 0.817 | 0.689 | 0.696 |
| CDK | DT | 0.606 | 0.681 | 0.341 | 0.627 | 0.655 | 0.723 | 0.389 | 0.664 |
|  | RF | 0.749 | 0.862 | 0.365 | 0.731 | 0.775 | 0.887 | 0.439 | 0.774 |
|  | AdaBoost | 0.557 | 0.619 | 0.290 | 0.623 | 0.649 | 0.698 | 0.316 | 0.637 |
|  | CatBoost | 0.746 | 0.855 | 0.746 | 0.714 | 0.732 | 0.862 | 0.732 | 0.704 |
|  | GBT | 0.736 | 0.868 | 0.380 | 0.705 | 0.782 | 0.904 | 0.458 | 0.772 |
|  | XGBoost | 0.762 | 0.871 | 0.762 | 0.730 | 0.754 | 0.870 | 0.754 | 0.728 |
|  | LightGBM | 0.756 | 0.871 | 0.415 | 0.735 | 0.779 | 0.901 | 0.499 | 0.783 |
|  | ET | 0.756 | 0.859 | 0.384 | 0.732 | 0.760 | 0.891 | 0.444 | 0.754 |
|  | LR | 0.731 | 0.842 | 0.424 | 0.708 | 0.769 | 0.886 | 0.511 | 0.759 |
|  | linearSVM | 0.702 | 0.845 | 0.702 | 0.702 | 0.711 | 0.833 | 0.711 | 0.706 |
| Pubchem | DT | 0.657 | 0.712 | 0.416 | 0.663 | 0.662 | 0.722 | 0.427 | 0.677 |
|  | RF | 0.766 | 0.861 | 0.389 | 0.750 | 0.763 | 0.904 | 0.451 | 0.772 |
|  | AdaBoost | 0.618 | 0.614 | 0.313 | 0.622 | 0.582 | 0.667 | 0.306 | 0.648 |
|  | CatBoost | 0.757 | 0.864 | 0.757 | 0.738 | 0.751 | 0.861 | 0.751 | 0.728 |
|  | GBT | 0.740 | 0.861 | 0.414 | 0.722 | 0.794 | 0.909 | 0.524 | 0.797 |
|  | XGBoost | 0.765 | 0.881 | 0.765 | 0.742 | 0.772 | 0.877 | 0.772 | 0.767 |
|  | LightGBM | 0.765 | 0.868 | 0.418 | 0.737 | 0.779 | 0.896 | 0.507 | 0.769 |
|  | ET | 0.756 | 0.857 | 0.409 | 0.736 | 0.775 | 0.886 | 0.465 | 0.771 |
|  | LR | 0.731 | 0.842 | 0.424 | 0.708 | 0.769 | 0.886 | 0.511 | 0.759 |
|  | linearSVM | 0.675 | 0.830 | 0.675 | 0.688 | 0.705 | 0.827 | 0.705 | 0.712 |

Table S5.

The evaluation result of B-McoL models.

| **Descriptor** | **Algorithm** | **CV** | | | | **Test** | | | |
| --- | --- | --- | --- | --- | --- | --- | --- | --- | --- |
|  |  | **ACC** | **AUC** | **Recall** | **Precision** | **ACC** | **AUC** | **Recall** | **Precision** |
| 2D | DT | 0.839 | 0.839 | 0.823 | 0.858 | 0.849 | 0.846 | 0.800 | 0.863 |
|  | RF | 0.949 | 0.979 | 0.968 | 0.934 | 0.957 | 0.990 | 0.957 | 0.957 |
|  | AdaBoost | 0.890 | 0.950 | 0.885 | 0.898 | 0.917 | 0.966 | 0.920 | 0.902 |
|  | CatBoost | 0.939 | 0.974 | 0.951 | 0.931 | 0.957 | 0.978 | 0.981 | 0.932 |
|  | GBT | 0.938 | 0.973 | 0.961 | 0.922 | 0.963 | 0.987 | 0.973 | 0.948 |
|  | XGBoost | 0.941 | 0.978 | 0.951 | 0.934 | 0.951 | 0.978 | 0.961 | 0.937 |
|  | LightGBM | 0.941 | 0.980 | 0.961 | 0.927 | 0.963 | 0.982 | 0.981 | 0.944 |
|  | ET | 0.889 | 0.957 | 0.843 | 0.933 | 0.942 | 0.991 | 0.913 | 0.958 |
|  | LR | 0.892 | 0.931 | 0.891 | 0.897 | 0.905 | 0.957 | 0.900 | 0.894 |
|  | linearSVM | 0.911 | 0.950 | 0.938 | 0.894 | 0.929 | 0.967 | 0.953 | 0.899 |
| MACCS | DT | 0.860 | 0.864 | 0.803 | 0.912 | 0.880 | 0.881 | 0.813 | 0.917 |
|  | RF | 0.918 | 0.967 | 0.918 | 0.925 | 0.938 | 0.981 | 0.900 | 0.964 |
|  | AdaBoost | 0.816 | 0.884 | 0.778 | 0.851 | 0.846 | 0.891 | 0.773 | 0.879 |
|  | CatBoost | 0.915 | 0.961 | 0.903 | 0.929 | 0.951 | 0.983 | 0.927 | 0.965 |
|  | GBT | 0.883 | 0.937 | 0.858 | 0.908 | 0.880 | 0.943 | 0.820 | 0.911 |
|  | XGBoost | 0.913 | 0.959 | 0.903 | 0.925 | 0.923 | 0.980 | 0.873 | 0.956 |
|  | LightGBM | 0.907 | 0.960 | 0.897 | 0.918 | 0.926 | 0.977 | 0.880 | 0.957 |
|  | ET | 0.896 | 0.956 | 0.865 | 0.926 | 0.926 | 0.970 | 0.880 | 0.957 |
|  | LR | 0.861 | 0.910 | 0.831 | 0.891 | 0.871 | 0.903 | 0.793 | 0.915 |
|  | linearSVM | 0.851 | 0.901 | 0.817 | 0.883 | 0.871 | 0.892 | 0.793 | 0.915 |
| ECFP4 | DT | 0.858 | 0.859 | 0.802 | 0.911 | 0.911 | 0.907 | 0.860 | 0.942 |
|  | RF | 0.917 | 0.970 | 0.917 | 0.918 | 0.942 | 0.977 | 0.933 | 0.940 |
|  | AdaBoost | 0.871 | 0.932 | 0.834 | 0.905 | 0.905 | 0.942 | 0.873 | 0.916 |
|  | CatBoost | 0.910 | 0.966 | 0.911 | 0.913 | 0.945 | 0.976 | 0.947 | 0.934 |
|  | GBT | 0.892 | 0.956 | 0.882 | 0.905 | 0.923 | 0.970 | 0.893 | 0.937 |
|  | XGBoost | 0.898 | 0.965 | 0.894 | 0.905 | 0.942 | 0.979 | 0.933 | 0.940 |
|  | LightGBM | 0.909 | 0.963 | 0.911 | 0.911 | 0.917 | 0.973 | 0.900 | 0.918 |
|  | ET | 0.893 | 0.965 | 0.864 | 0.922 | 0.898 | 0.969 | 0.853 | 0.921 |
|  | LR | 0.897 | 0.955 | 0.890 | 0.908 | 0.923 | 0.965 | 0.913 | 0.919 |
|  | linearSVM | 0.875 | 0.929 | 0.846 | 0.905 | 0.892 | 0.930 | 0.840 | 0.920 |
| AtomPair | DT | 0.837 | 0.838 | 0.766 | 0.899 | 0.898 | 0.893 | 0.820 | 0.953 |
|  | RF | 0.907 | 0.967 | 0.873 | 0.935 | 0.942 | 0.975 | 0.949 | 0.931 |
|  | AdaBoost | 0.884 | 0.940 | 0.844 | 0.921 | 0.886 | 0.940 | 0.840 | 0.906 |
|  | CatBoost | 0.937 | 0.979 | 0.930 | 0.945 | 0.957 | 0.980 | 0.968 | 0.943 |
|  | GBT | 0.929 | 0.975 | 0.920 | 0.941 | 0.966 | 0.992 | 0.953 | 0.973 |
|  | XGBoost | 0.948 | 0.982 | 0.948 | 0.950 | 0.954 | 0.982 | 0.975 | 0.933 |
|  | LightGBM | 0.950 | 0.978 | 0.961 | 0.943 | 0.957 | 0.994 | 0.960 | 0.947 |
|  | ET | 0.913 | 0.968 | 0.885 | 0.941 | 0.932 | 0.986 | 0.887 | 0.964 |
|  | LR | 0.924 | 0.973 | 0.909 | 0.940 | 0.951 | 0.989 | 0.927 | 0.965 |
|  | linearSVM | 0.909 | 0.961 | 0.882 | 0.936 | 0.945 | 0.971 | 0.927 | 0.952 |
| CDK | DT | 0.860 | 0.861 | 0.811 | 0.906 | 0.889 | 0.886 | 0.840 | 0.913 |
|  | RF | 0.931 | 0.975 | 0.941 | 0.933 | 0.951 | 0.980 | 0.953 | 0.941 |
|  | AdaBoost | 0.871 | 0.939 | 0.840 | 0.901 | 0.902 | 0.950 | 0.873 | 0.910 |
|  | CatBoost | 0.936 | 0.974 | 0.946 | 0.930 | 0.945 | 0.981 | 0.933 | 0.946 |
|  | GBT | 0.918 | 0.966 | 0.917 | 0.921 | 0.938 | 0.977 | 0.927 | 0.939 |
|  | XGBoost | 0.932 | 0.974 | 0.935 | 0.933 | 0.960 | 0.980 | 0.967 | 0.948 |
|  | LightGBM | 0.928 | 0.972 | 0.938 | 0.923 | 0.960 | 0.982 | 0.967 | 0.948 |
|  | ET | 0.905 | 0.970 | 0.882 | 0.929 | 0.917 | 0.972 | 0.887 | 0.930 |
|  | LR | 0.913 | 0.964 | 0.899 | 0.928 | 0.908 | 0.960 | 0.867 | 0.929 |
|  | linearSVM | 0.884 | 0.944 | 0.849 | 0.917 | 0.895 | 0.938 | 0.833 | 0.933 |
| Pubchem | DT | 0.878 | 0.880 | 0.853 | 0.903 | 0.877 | 0.880 | 0.827 | 0.899 |
|  | RF | 0.921 | 0.970 | 0.921 | 0.930 | 0.935 | 0.976 | 0.920 | 0.939 |
|  | AdaBoost | 0.833 | 0.916 | 0.791 | 0.871 | 0.852 | 0.914 | 0.793 | 0.875 |
|  | CatBoost | 0.904 | 0.961 | 0.887 | 0.922 | 0.938 | 0.970 | 0.920 | 0.945 |
|  | GBT | 0.880 | 0.947 | 0.859 | 0.902 | 0.886 | 0.938 | 0.827 | 0.919 |
|  | XGBoost | 0.912 | 0.968 | 0.903 | 0.923 | 0.932 | 0.977 | 0.920 | 0.932 |
|  | LightGBM | 0.908 | 0.969 | 0.897 | 0.922 | 0.923 | 0.973 | 0.900 | 0.931 |
|  | ET | 0.911 | 0.963 | 0.894 | 0.930 | 0.920 | 0.974 | 0.880 | 0.943 |
|  | LR | 0.889 | 0.943 | 0.871 | 0.908 | 0.911 | 0.953 | 0.887 | 0.917 |
|  | linearSVM | 0.863 | 0.894 | 0.826 | 0.897 | 0.895 | 0.925 | 0.853 | 0.914 |

**Table S6**.

Different functionals and their calculation results for the UV absorption.

| **Probe** | **PBE0** | **CAM-B3LYP** | **M06-2X** | **EXP** |
| --- | --- | --- | --- | --- |
| P1-ALP | 3.09 eV 400.24nm | 3.95 eV 313.49 nm | 2.79 eV 443.48 nm | 3.54eV 350 nm |
| P2-VIS | 2.55 eV 485.57 nm | 2.81 eV 441.05 nm | 2.79 eV 443.48 nm | 2.81 eV 440 nm |
| P3-ROS | 3.53 eV 350.33 nm | 4.16 eV 297.75 nm | 4.14 eV 298.90 nm | 3.44 eV360 nm |

Table S7.

Molecular structure, identifier, and predicted labels by our machine learning framework for the designed 21 ALP-probes. For the B-PvsC, “1” represents probe and “0” represents Np-Compounds; For the B-MvsP: “1” represents mitochondria-targeted one and “0” represents other organelle-targeted ones; For the M-PvsP: “0”, “1”, “2”, “3”, “4”, “5” represent Golgi apparatus, Endoplasmic reticulum, Lysosome, Cell membrane, Mitochondria and Nucleus targeted one. For the B-McoL: “1” represents Correlation >= 0.8 and “0” represents Correlation <0.8.


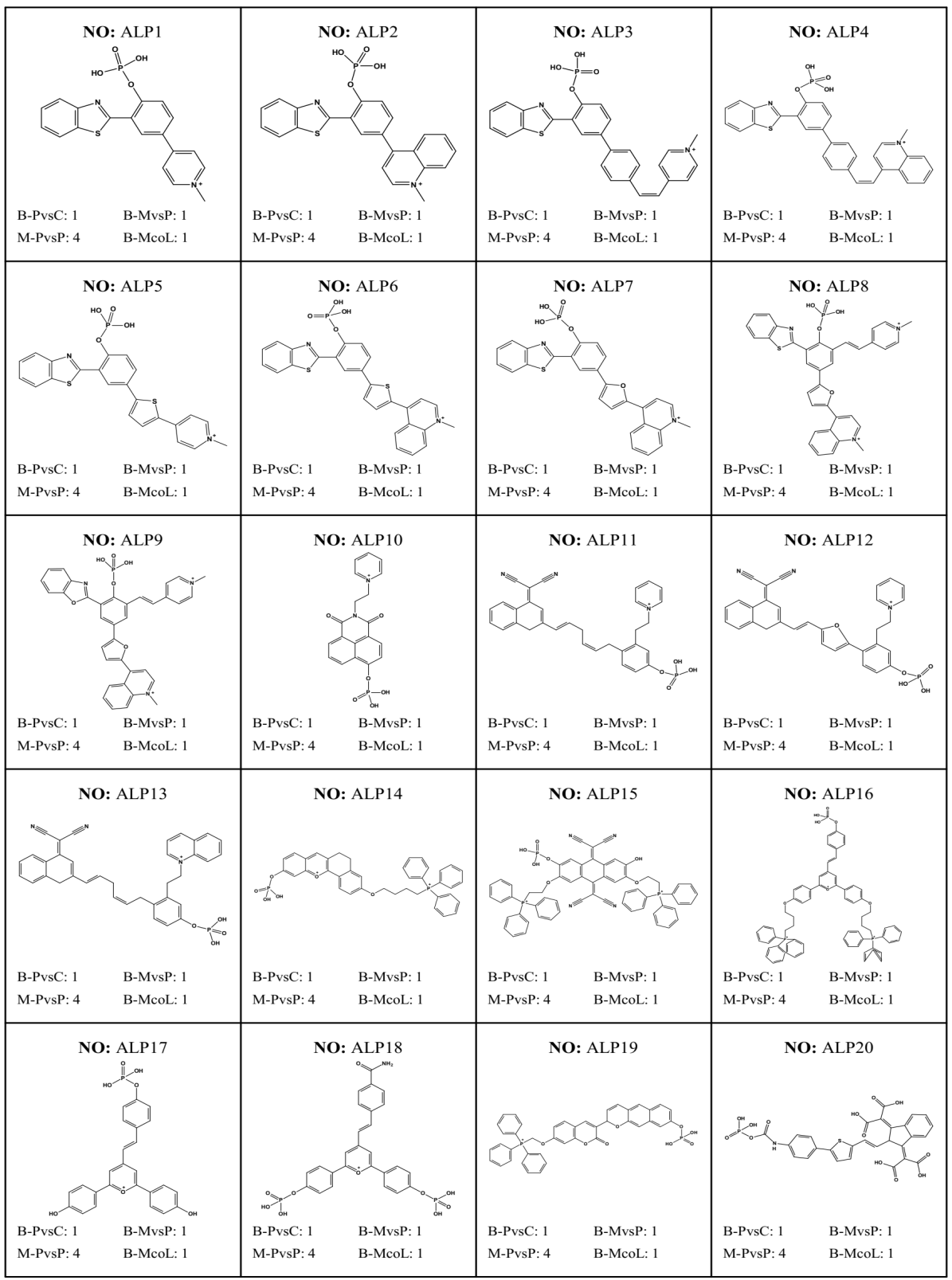


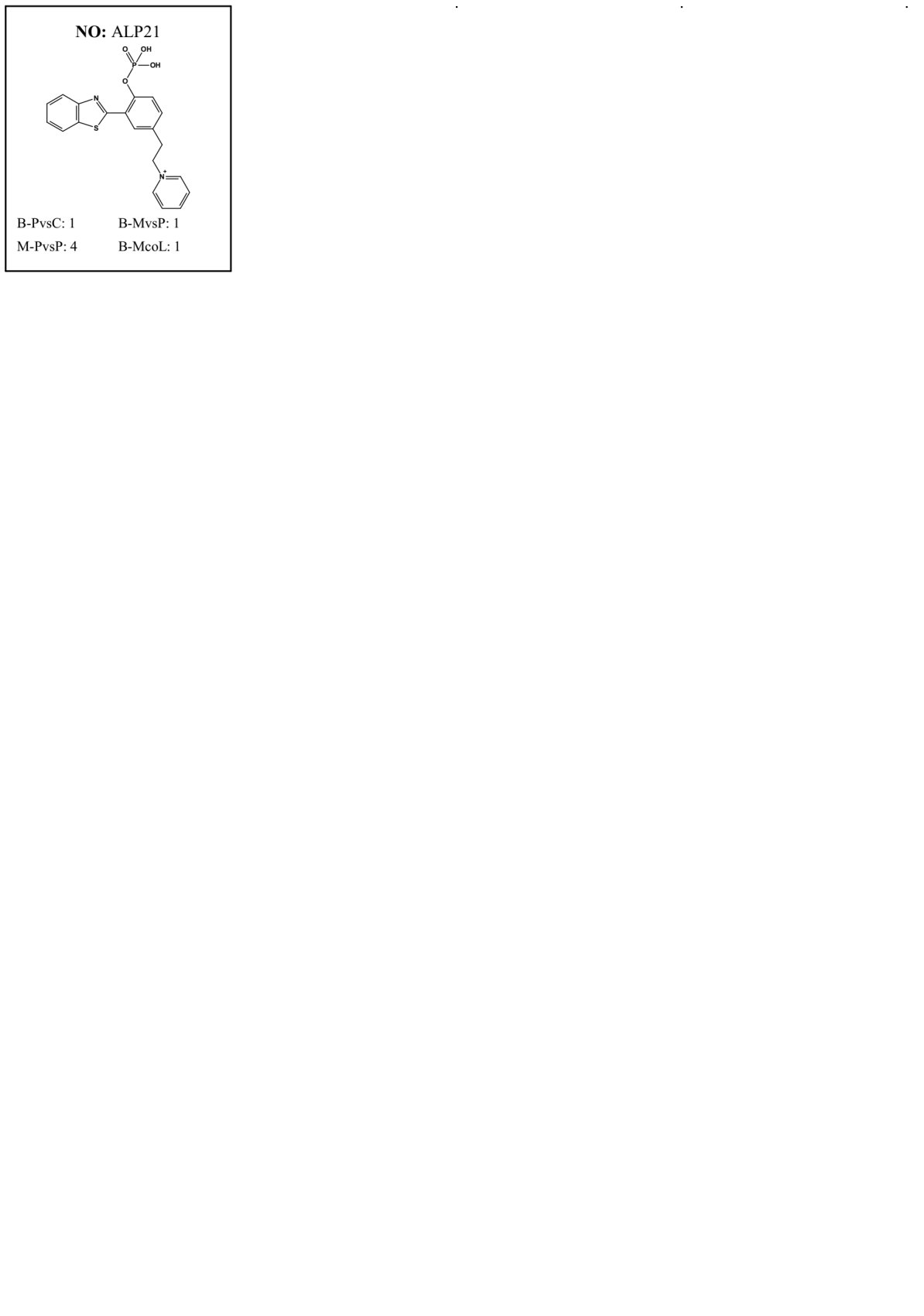


Table S8.

Molecular structure, identifier, and predicted labels by our machine learning framework for the designed 70 ESIPT-probes.


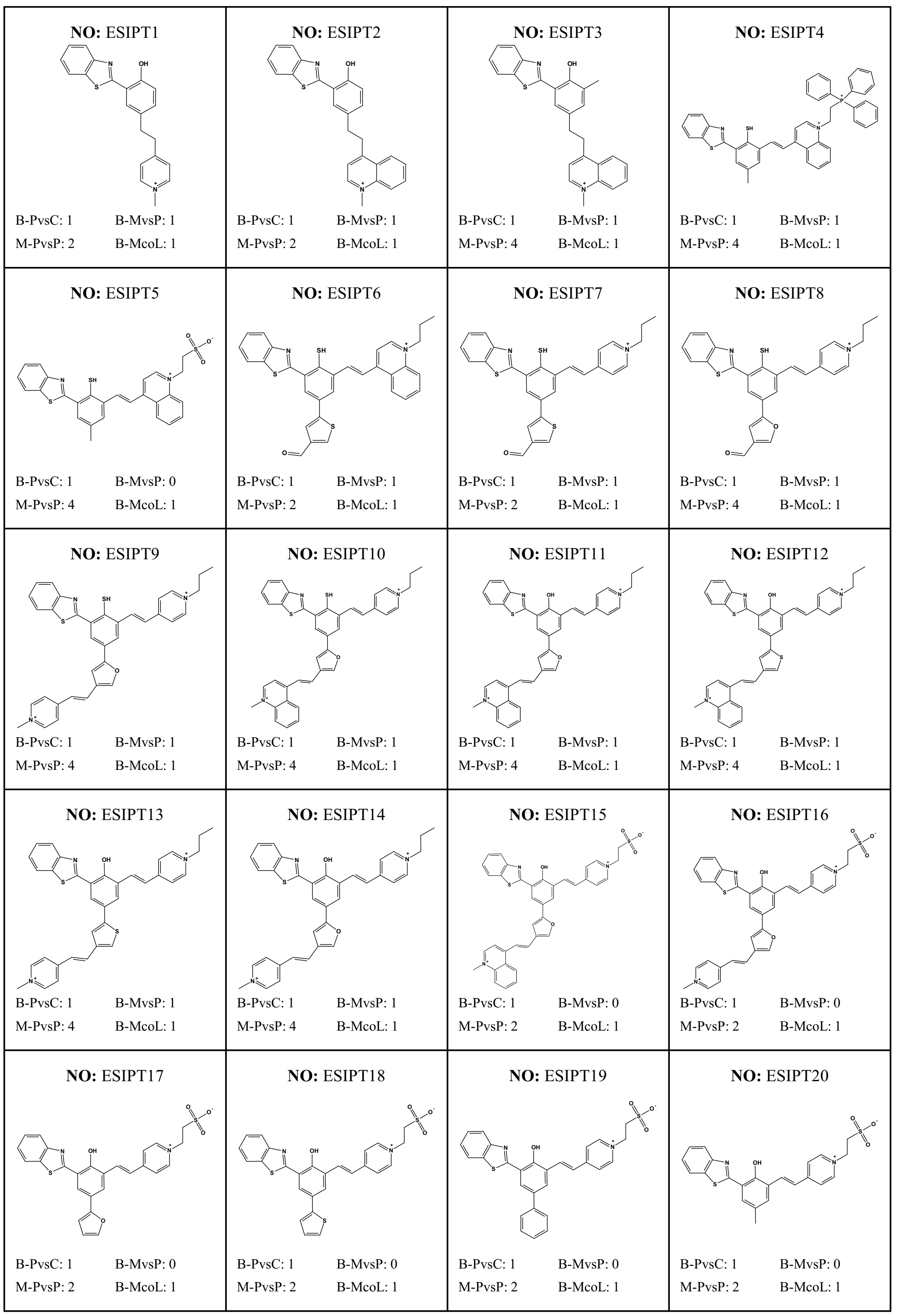


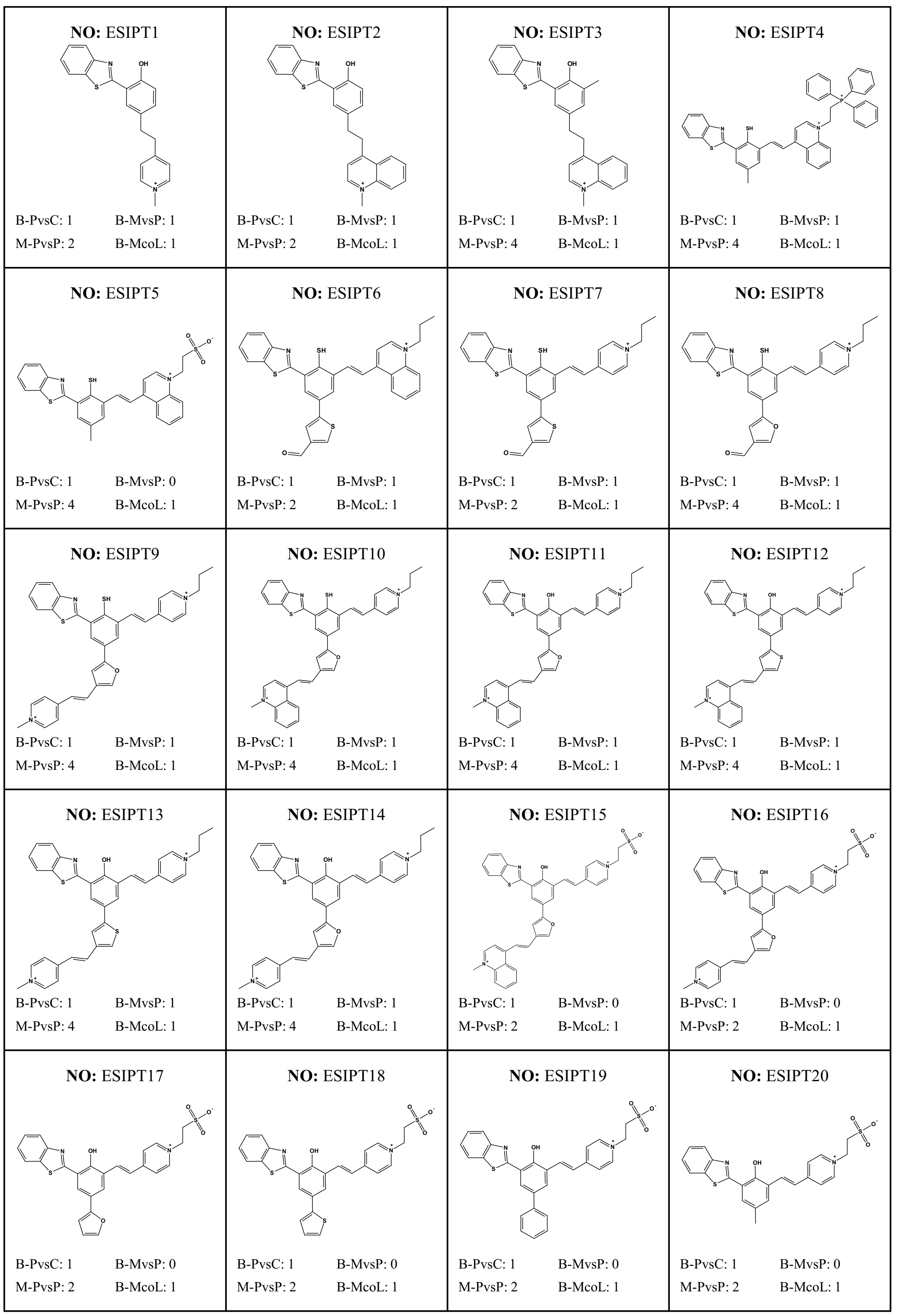


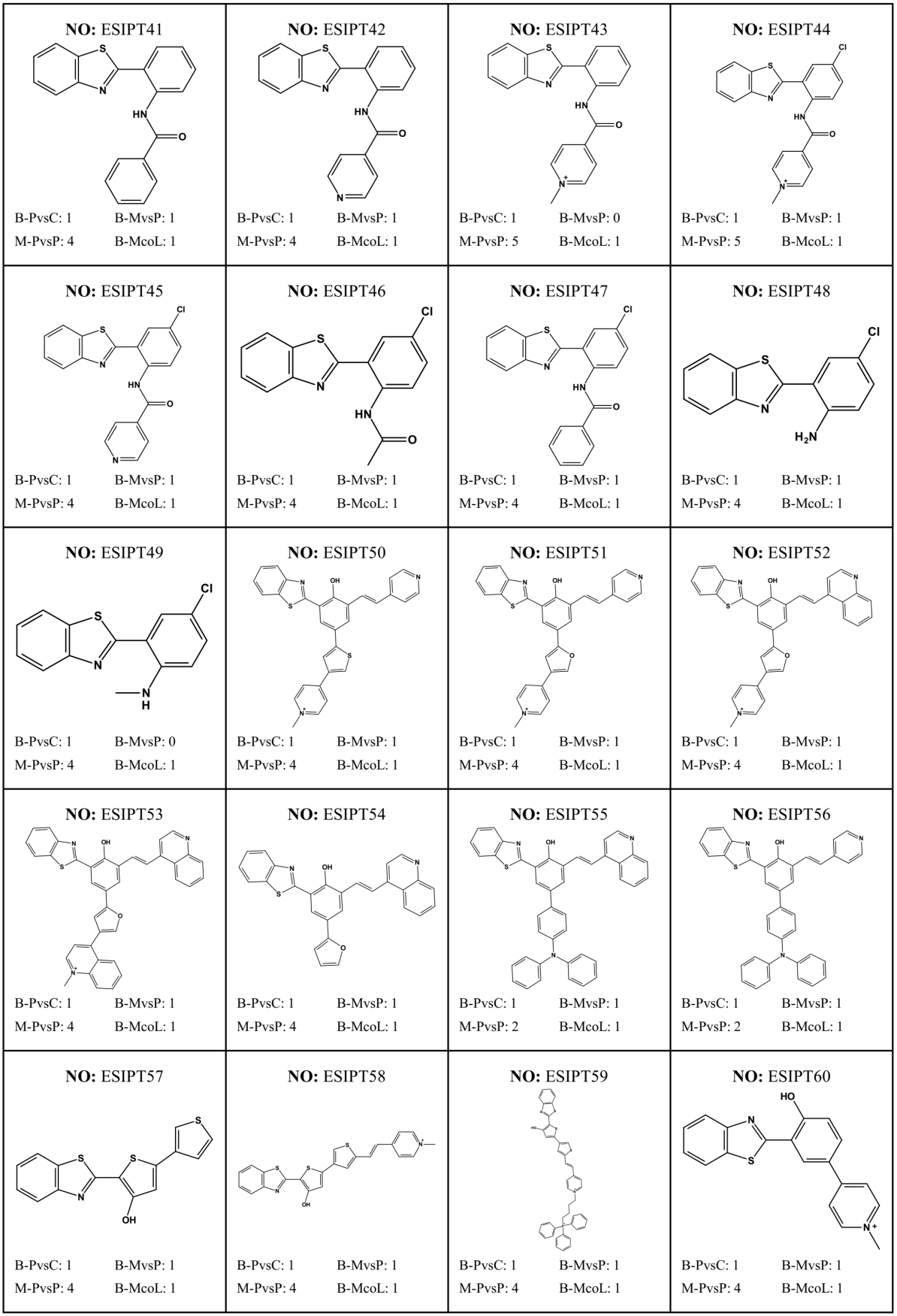


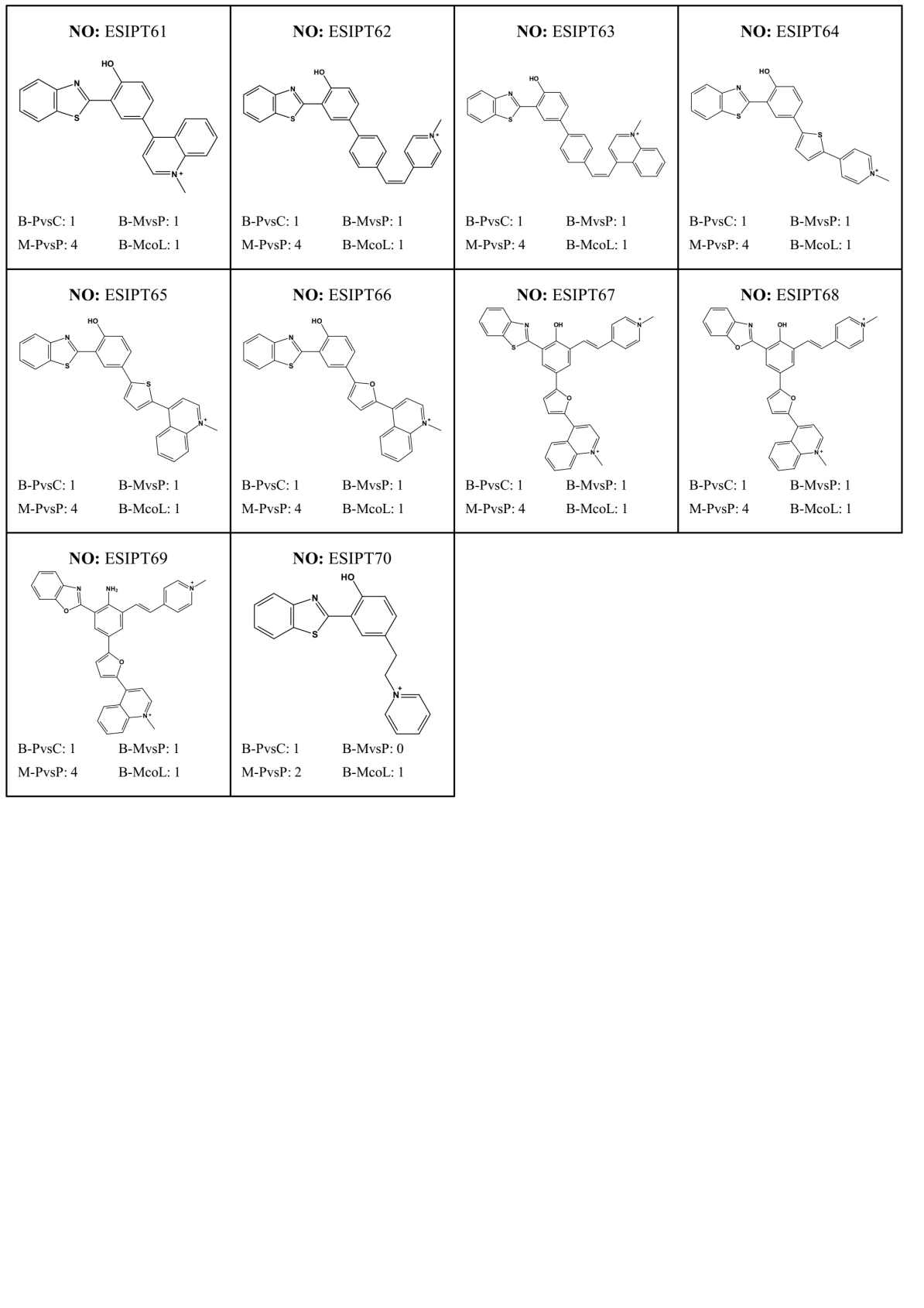


Table S9.

Molecular structure, identifier, and predicted labels by our machine learning framework for the designed 451 ICT-probes.


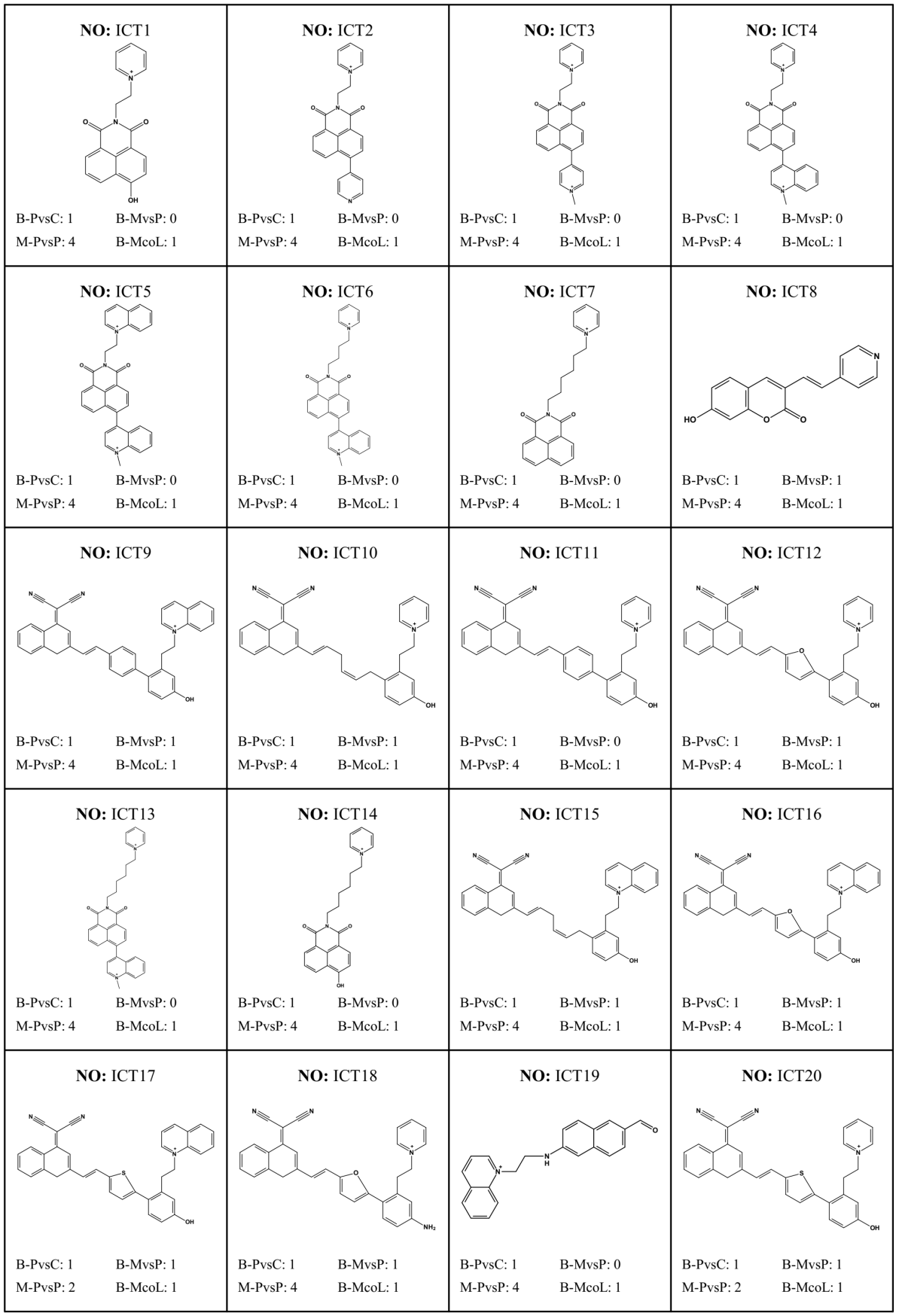


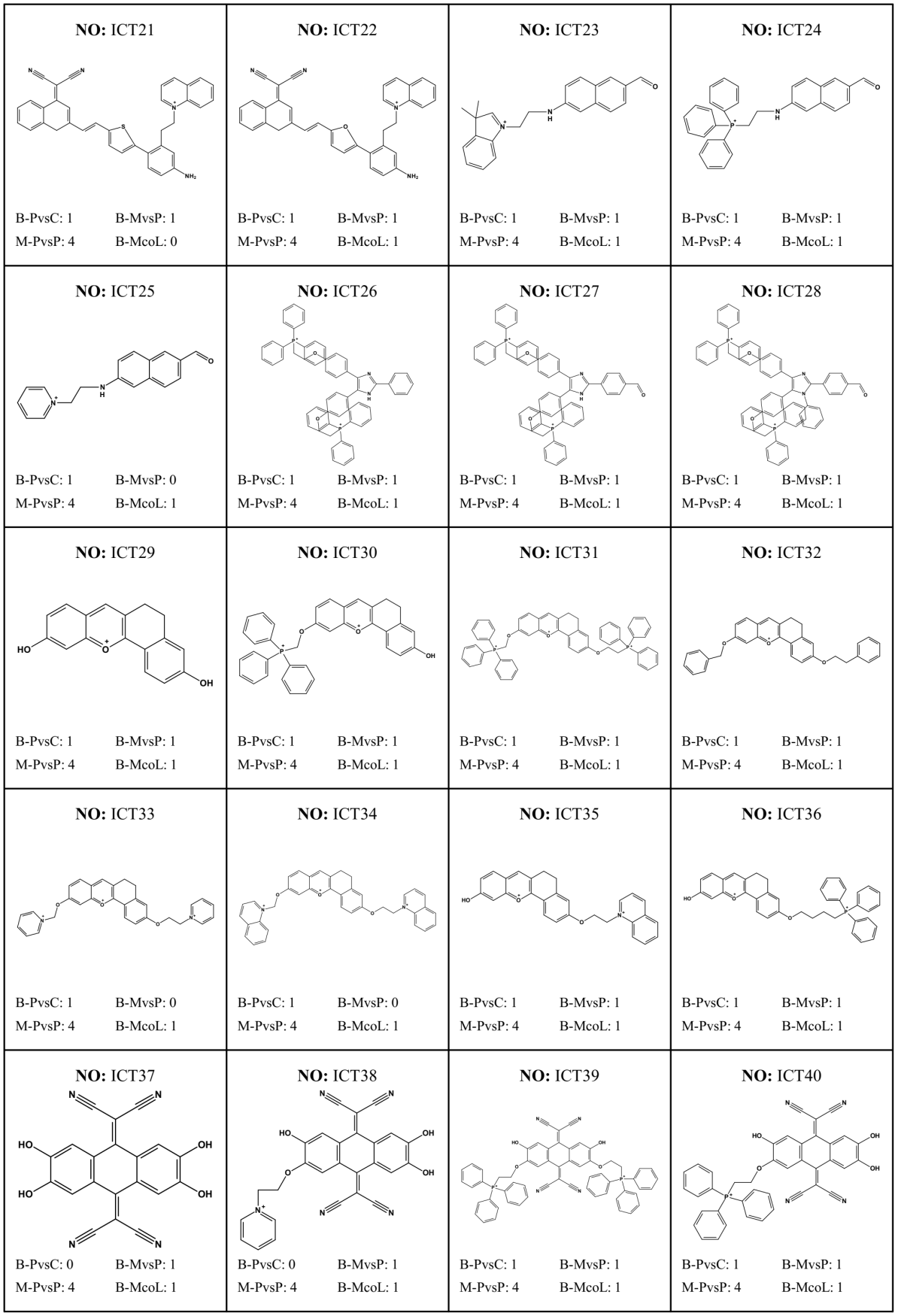


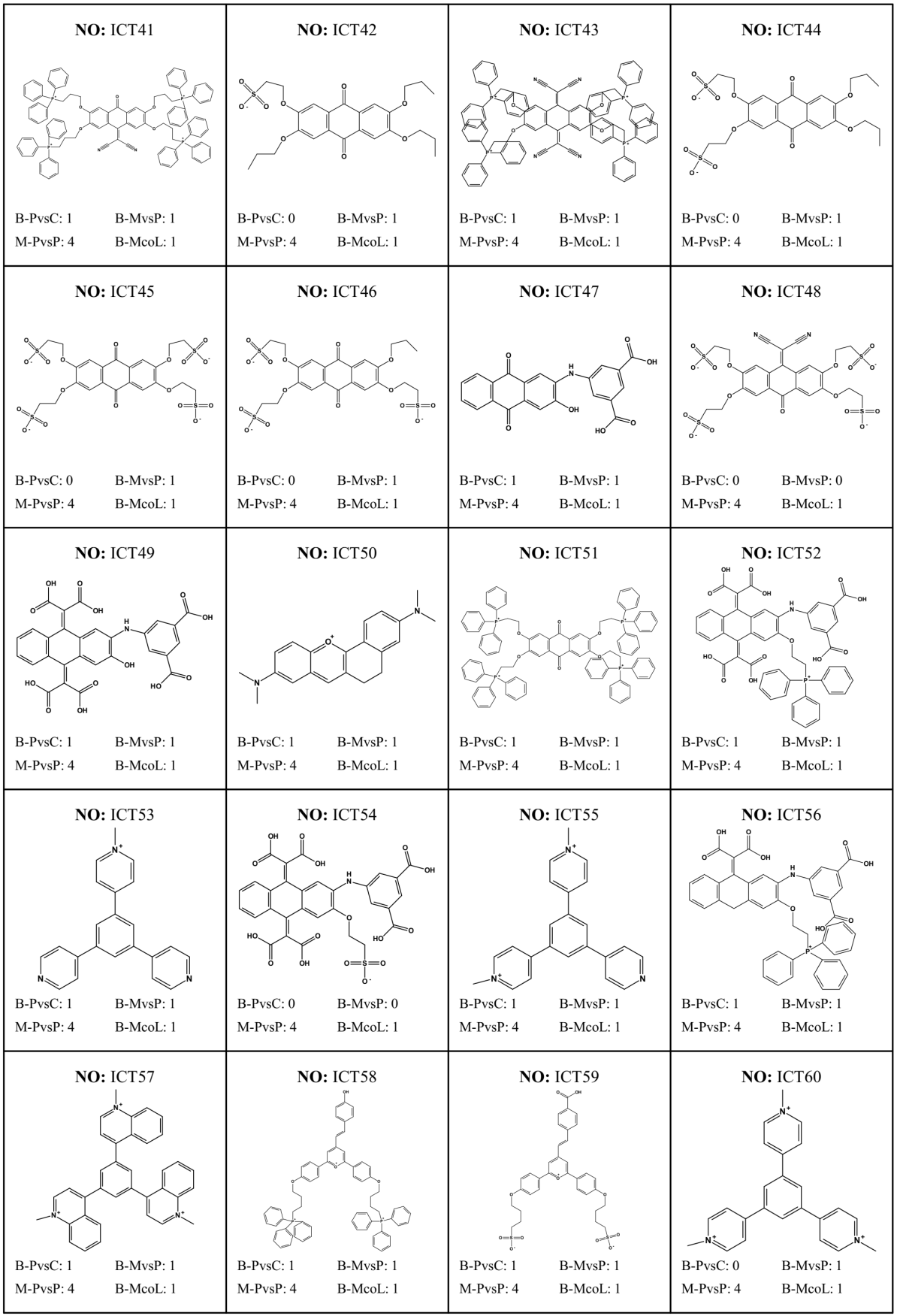


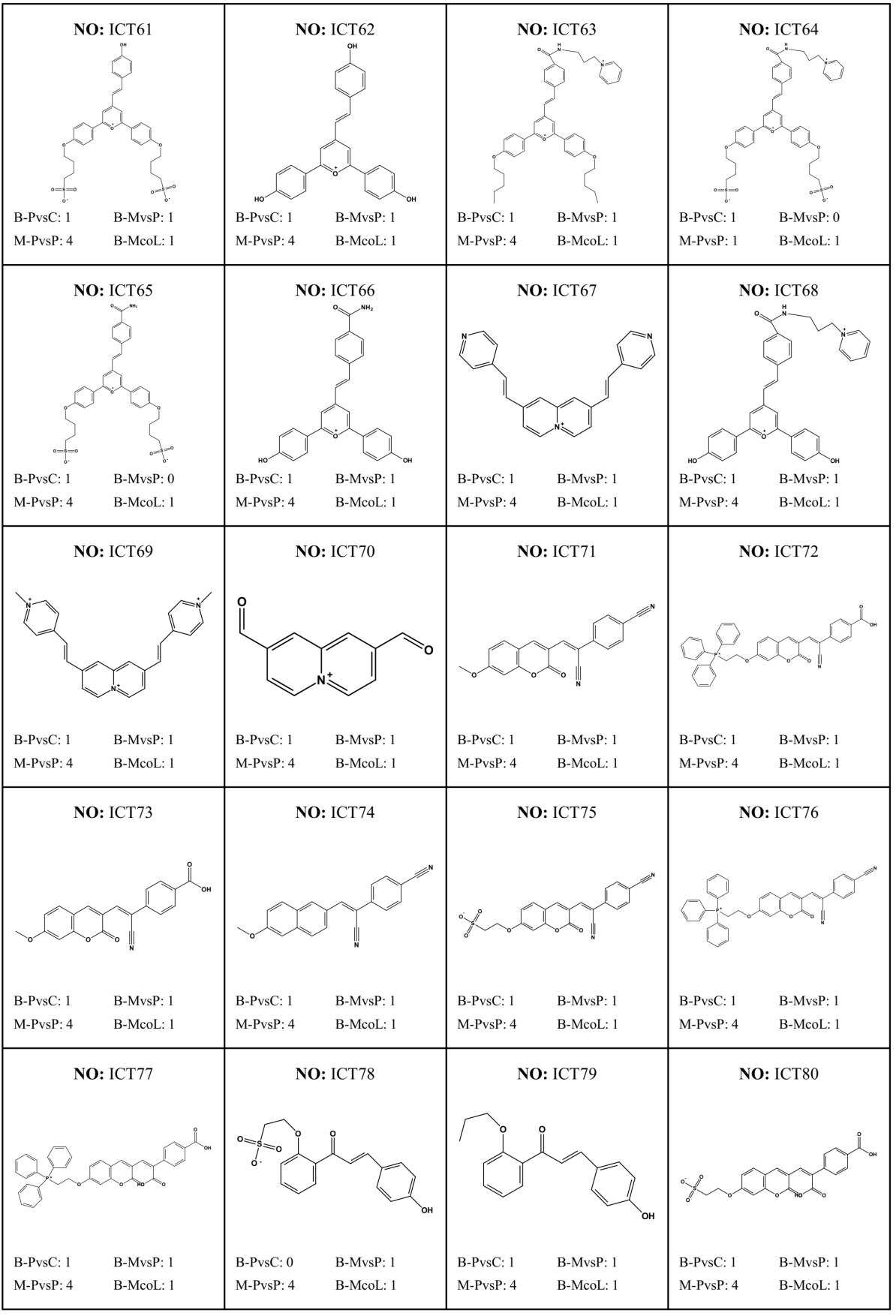


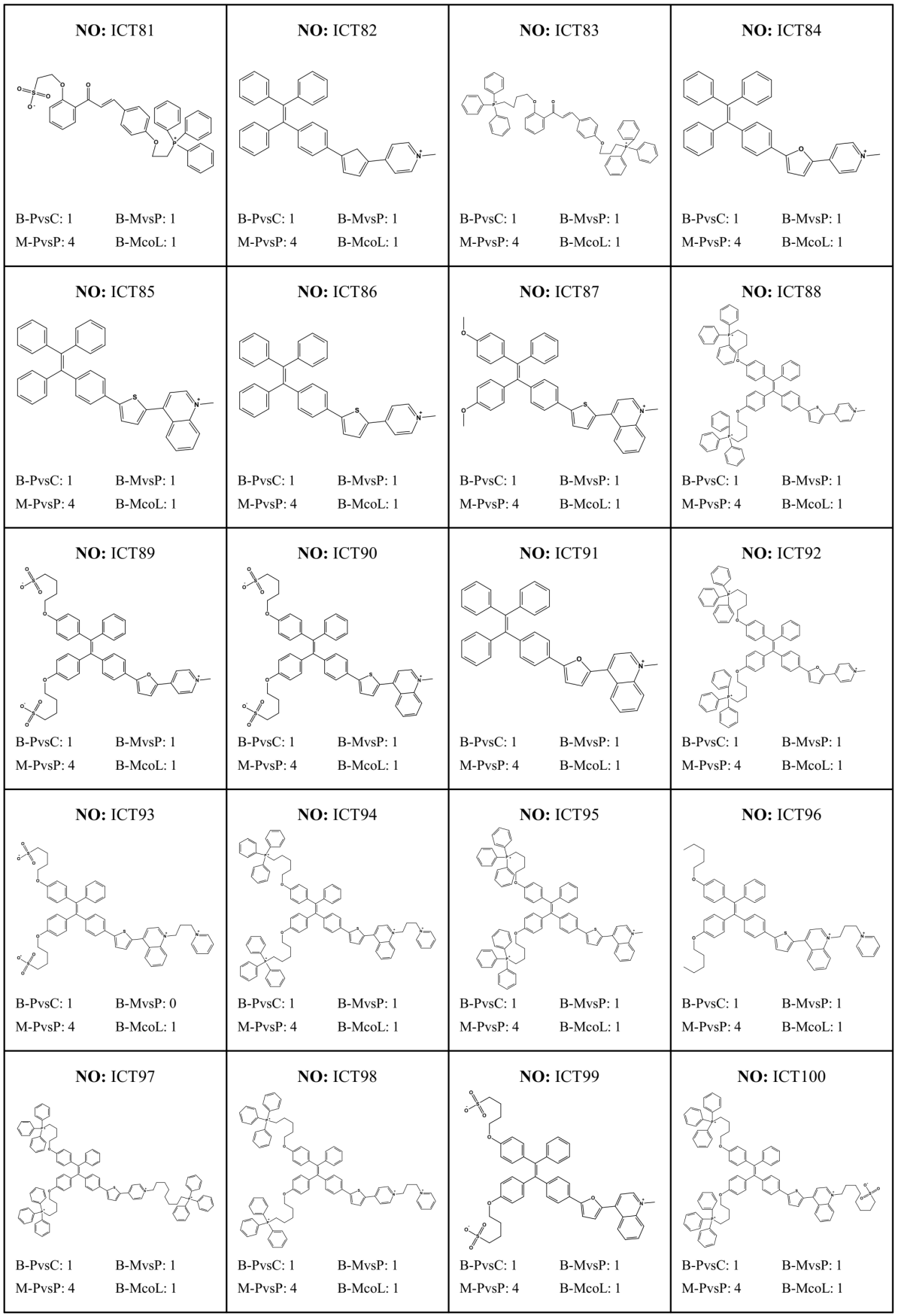


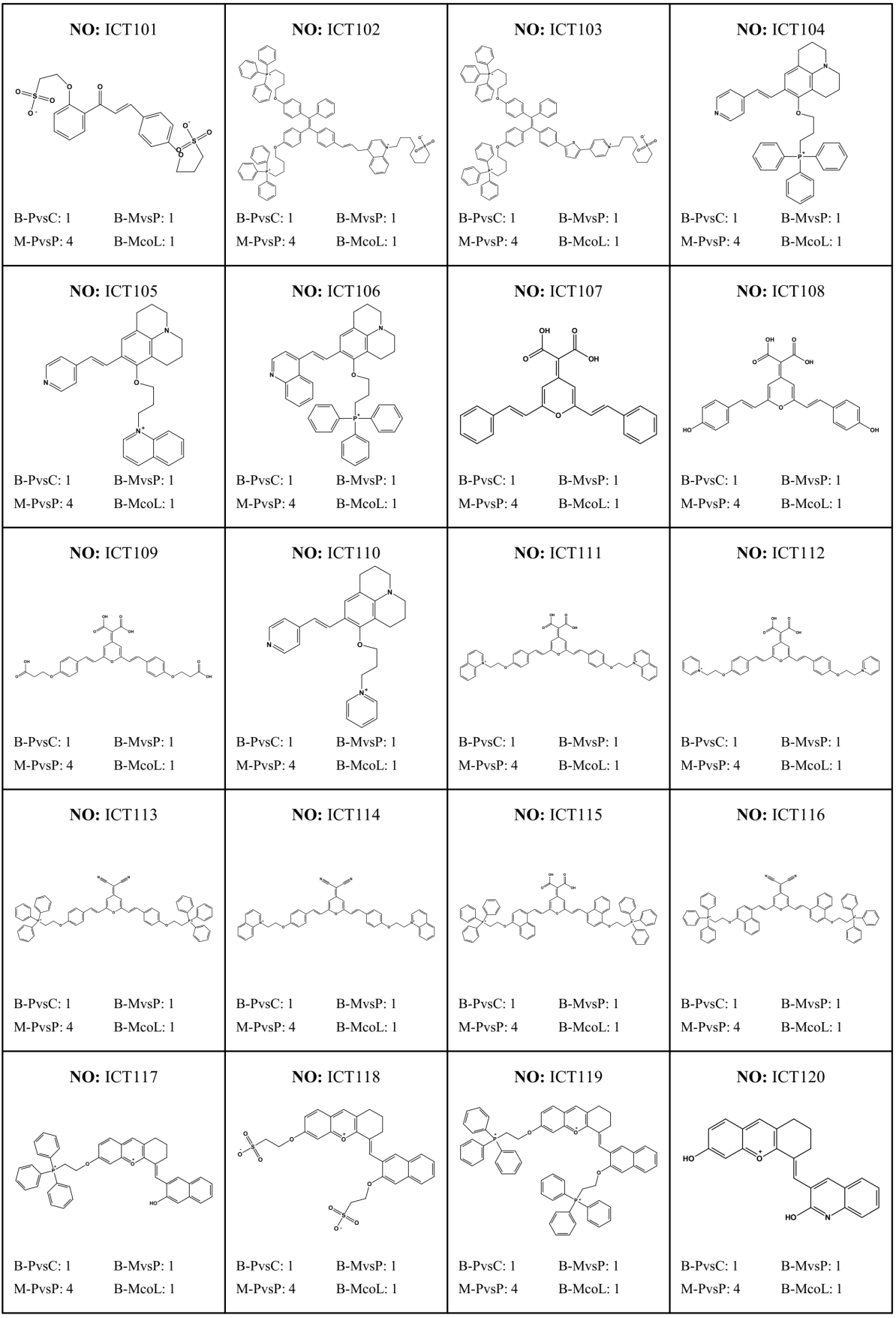


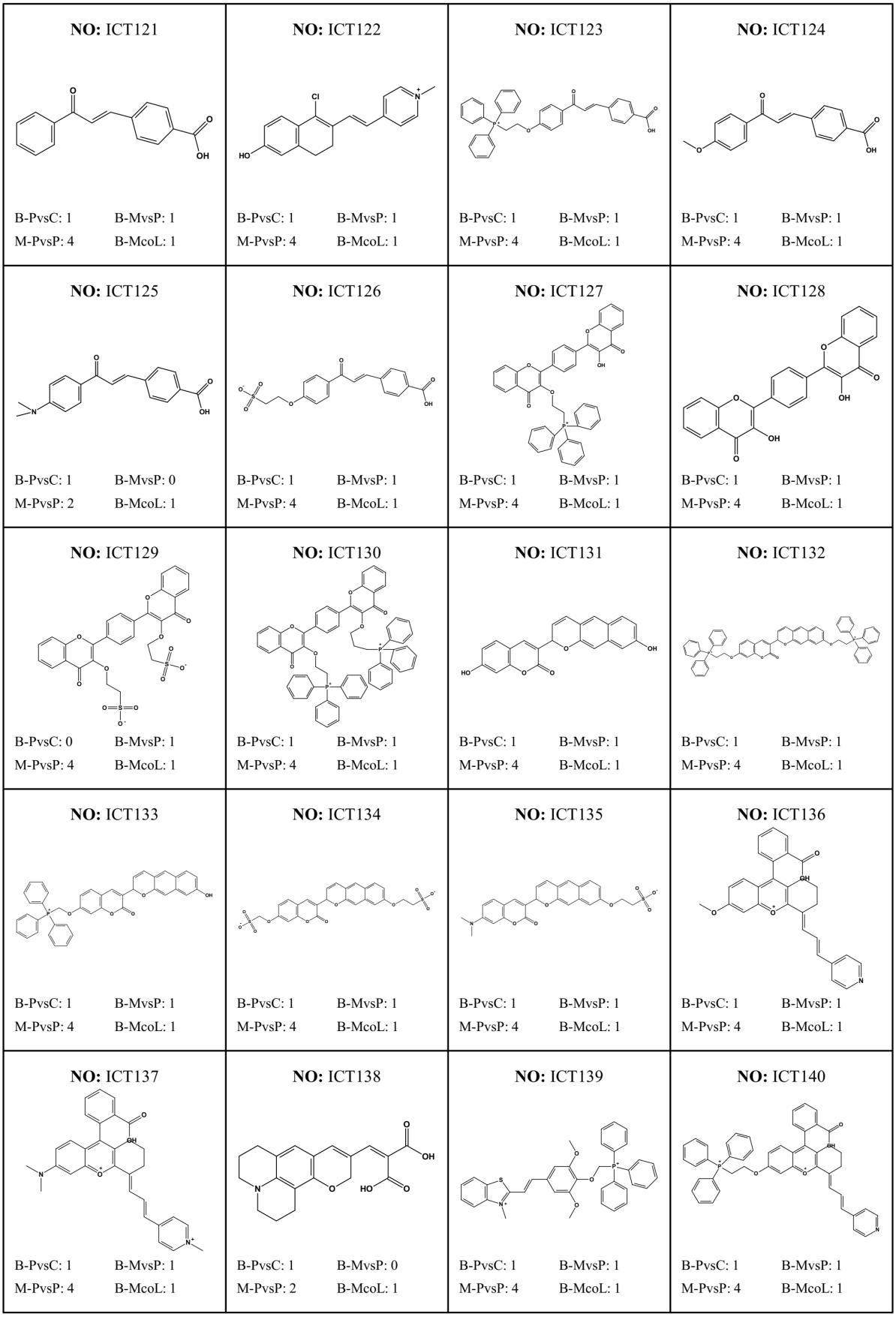


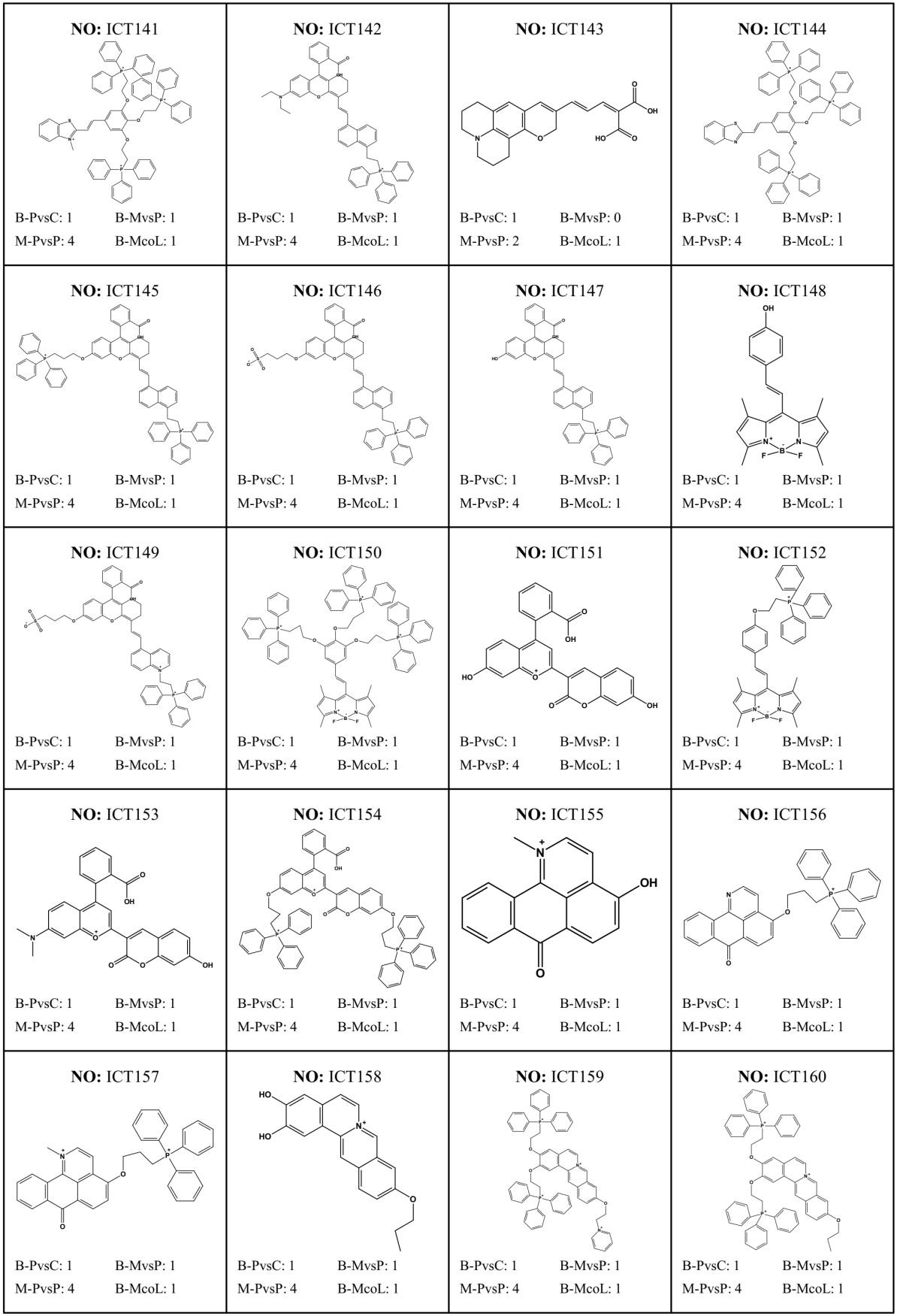


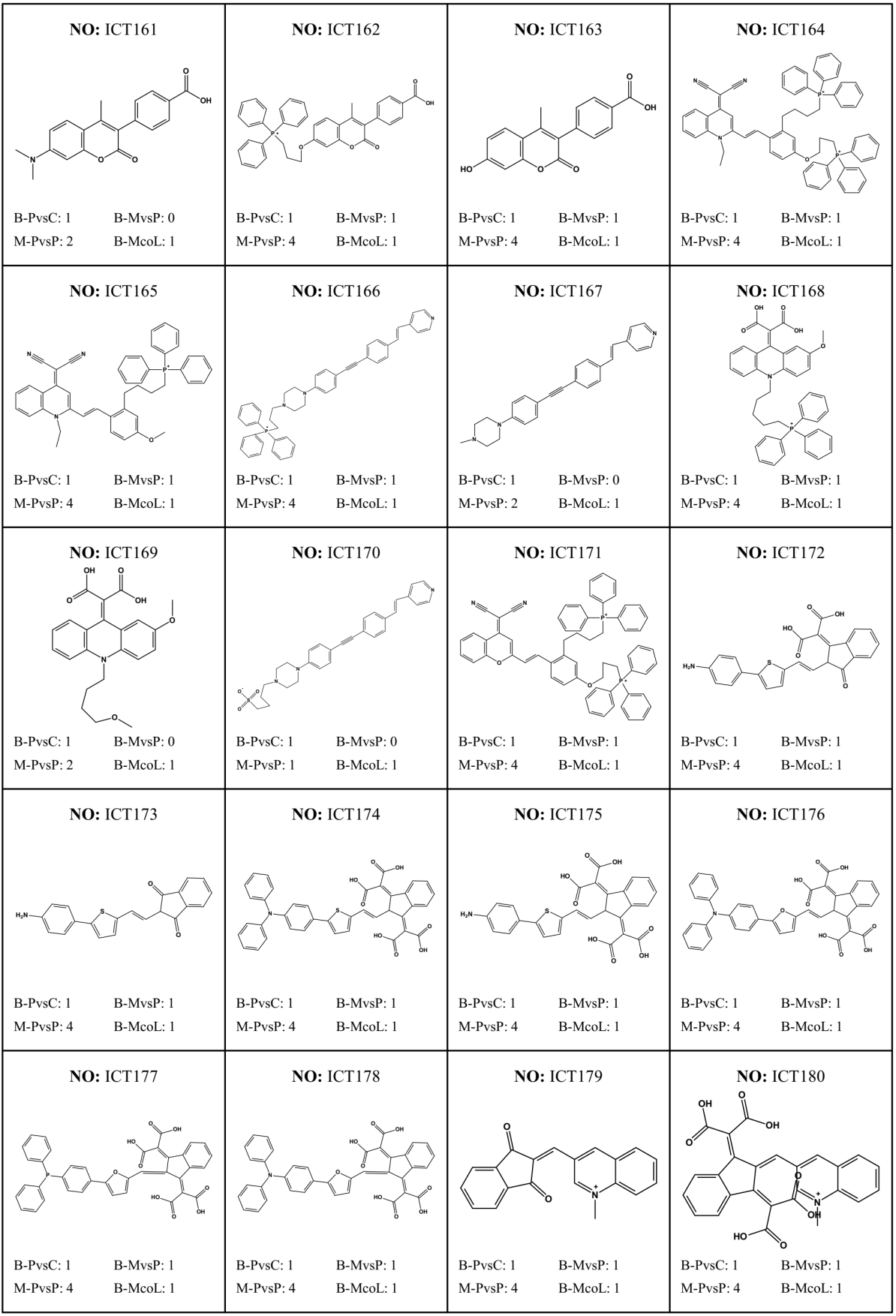


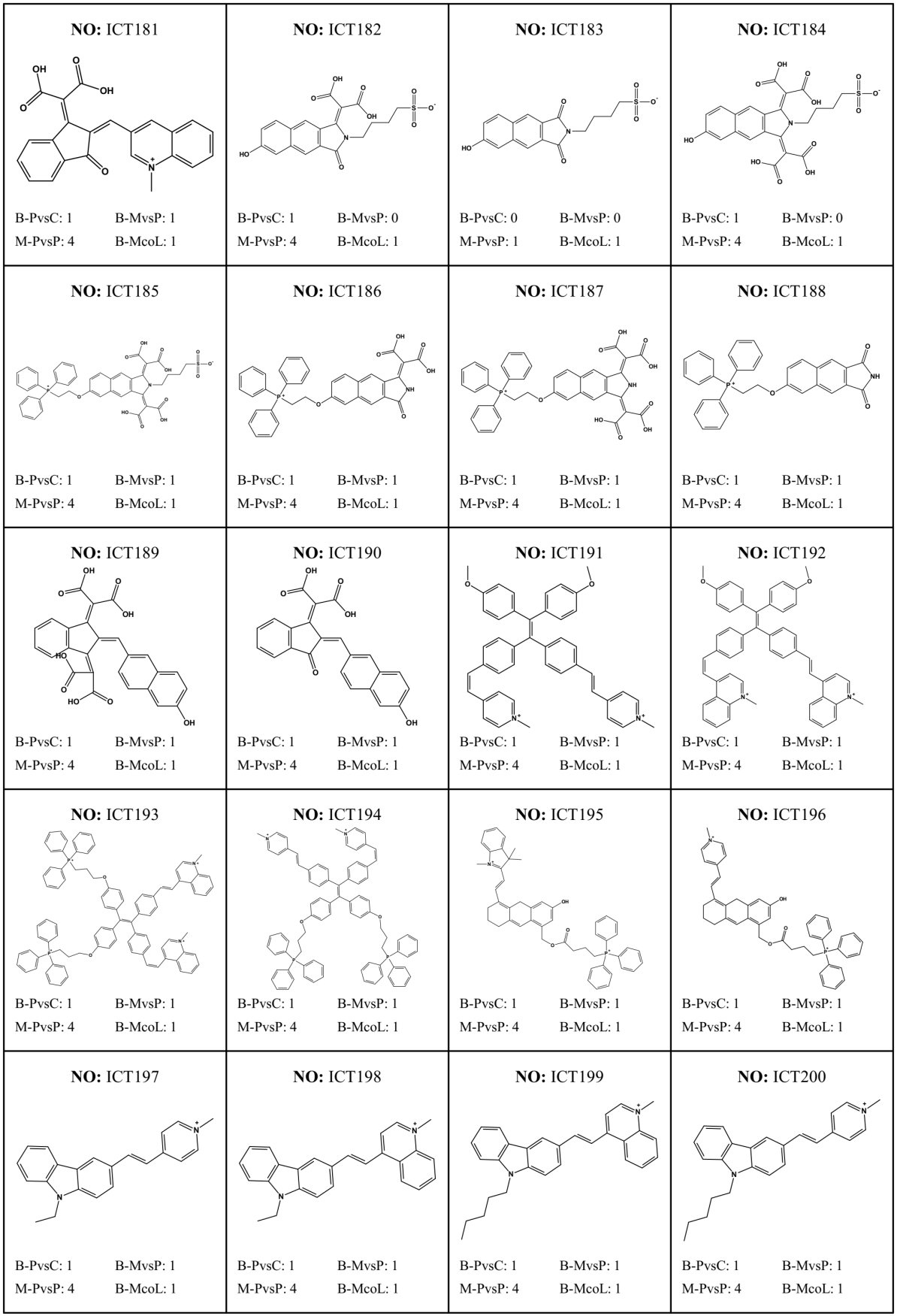


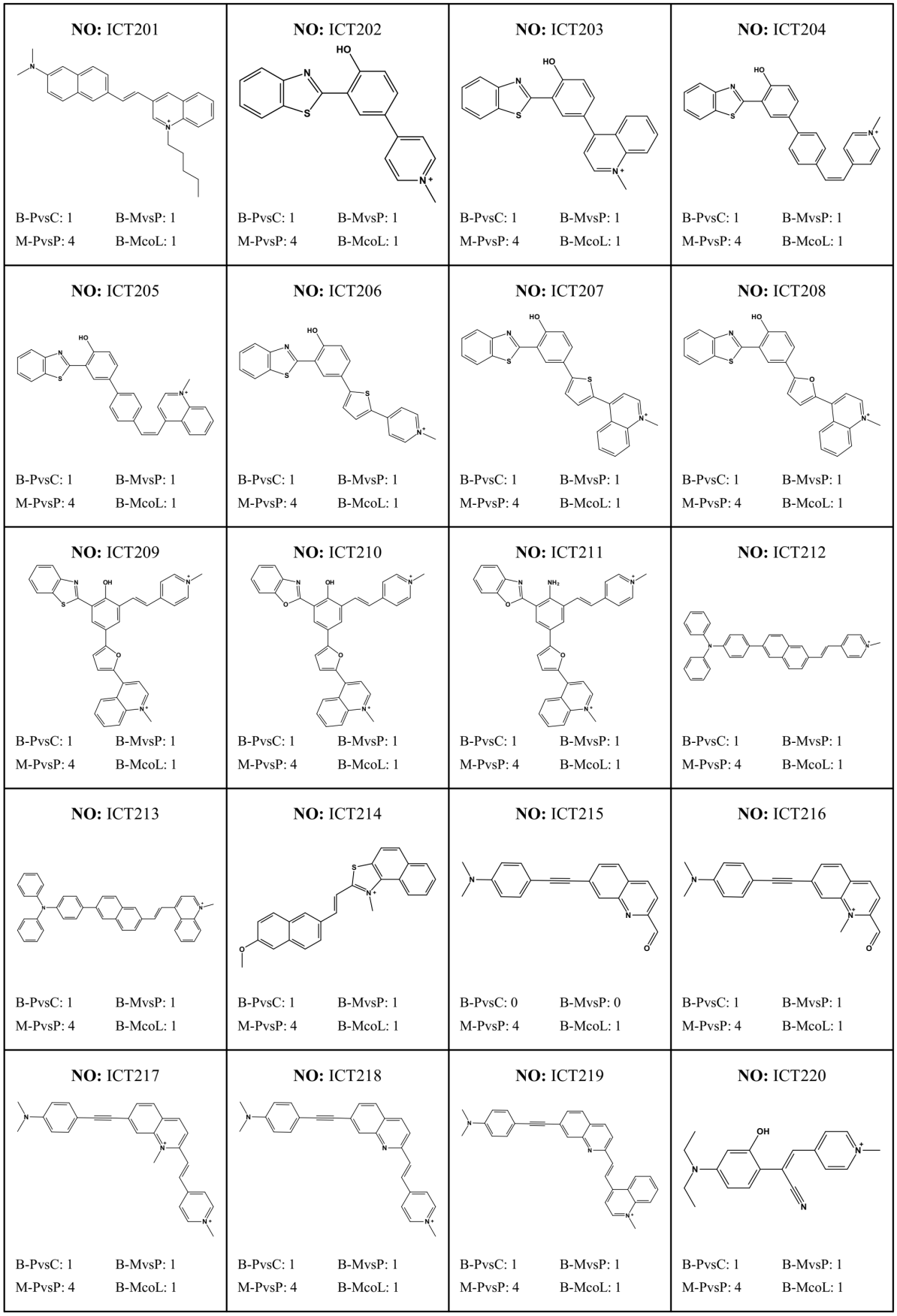


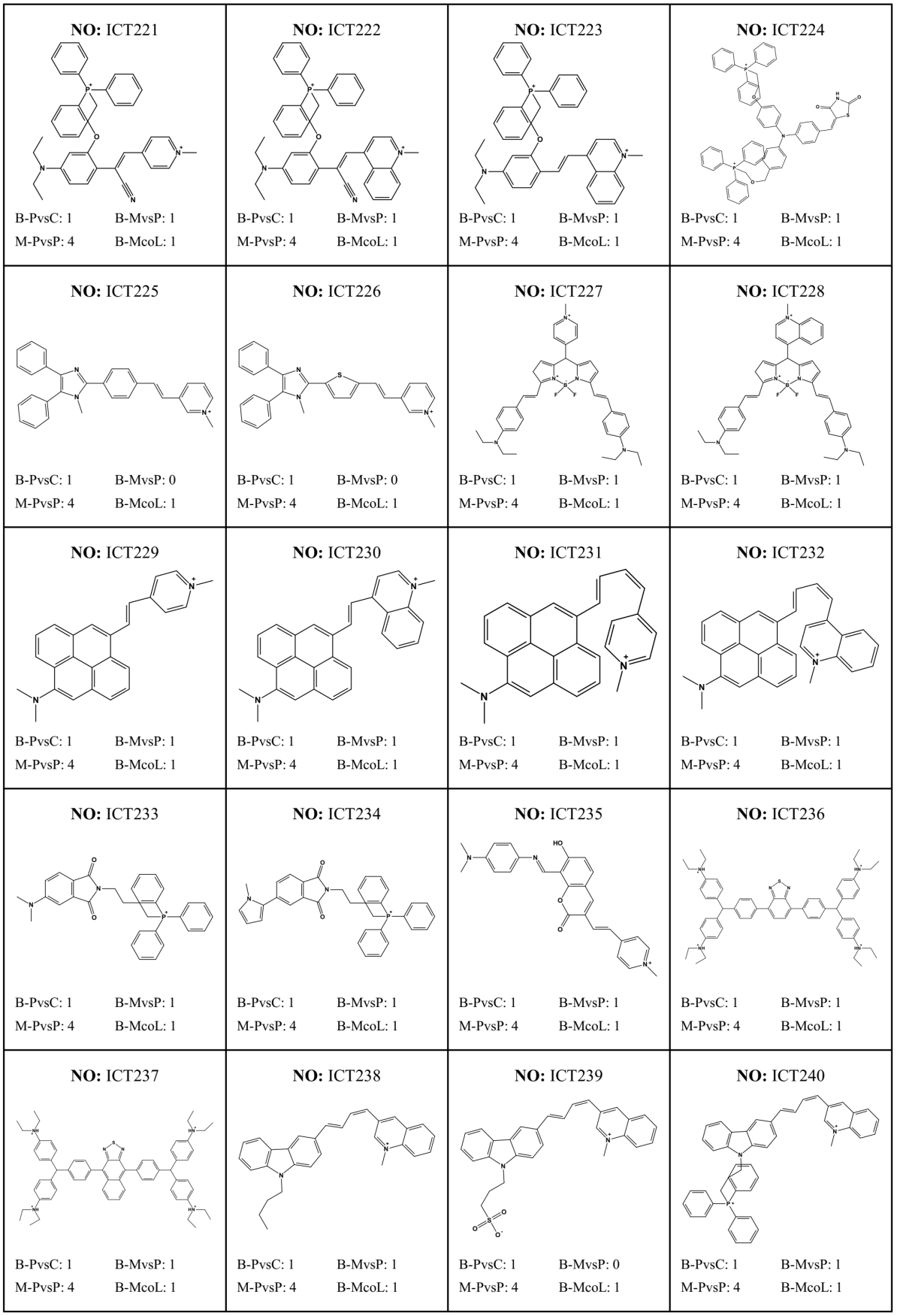


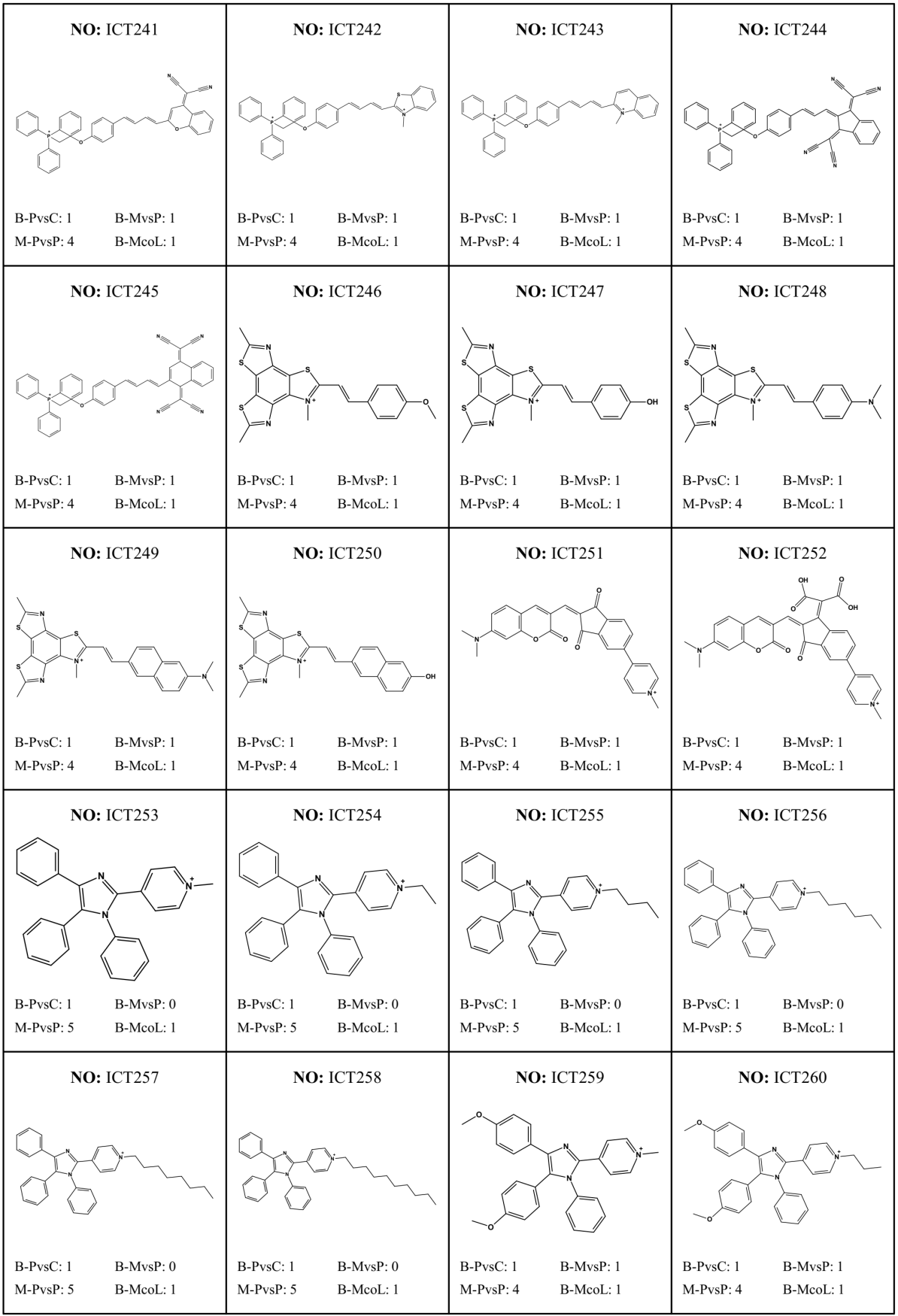


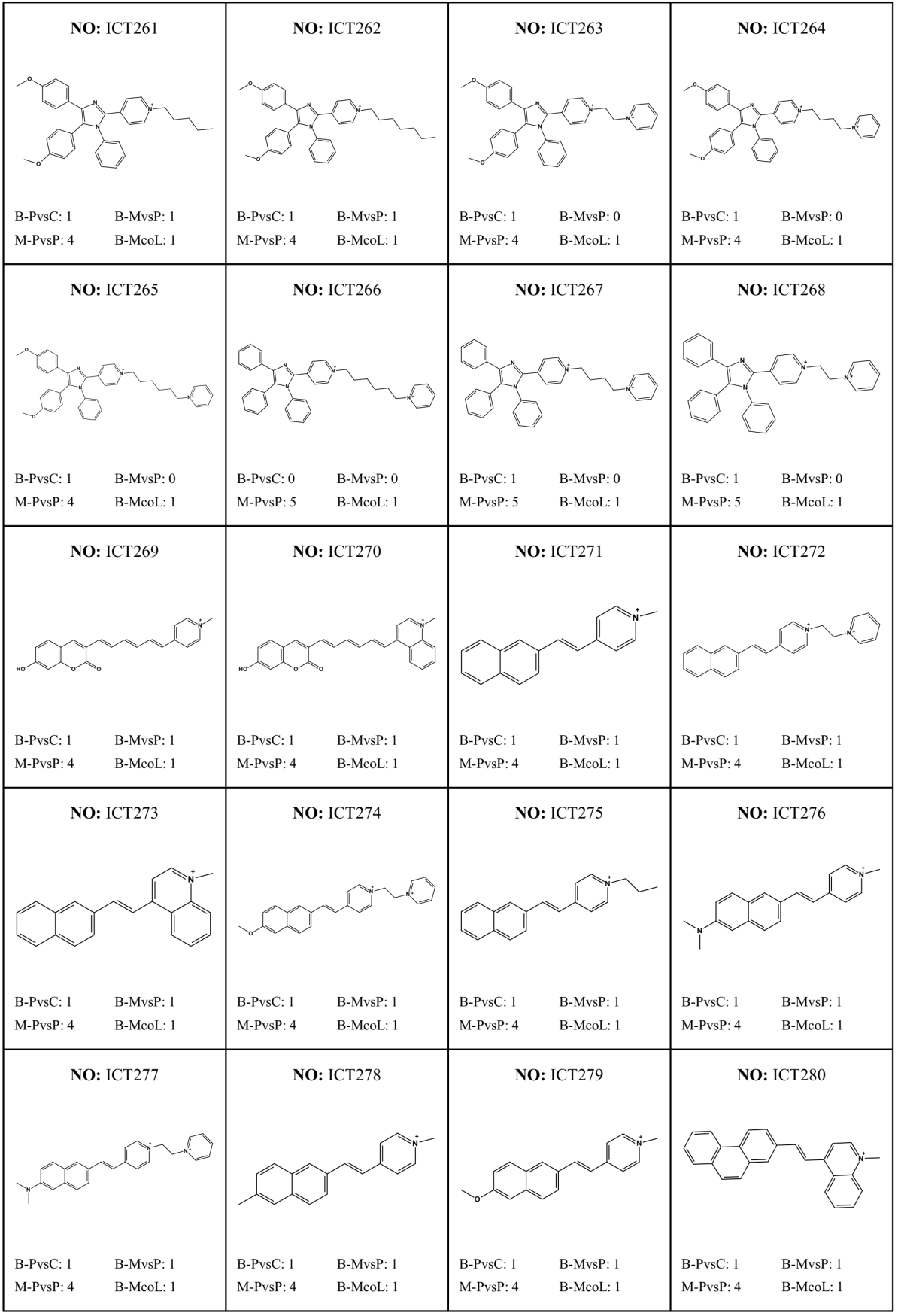


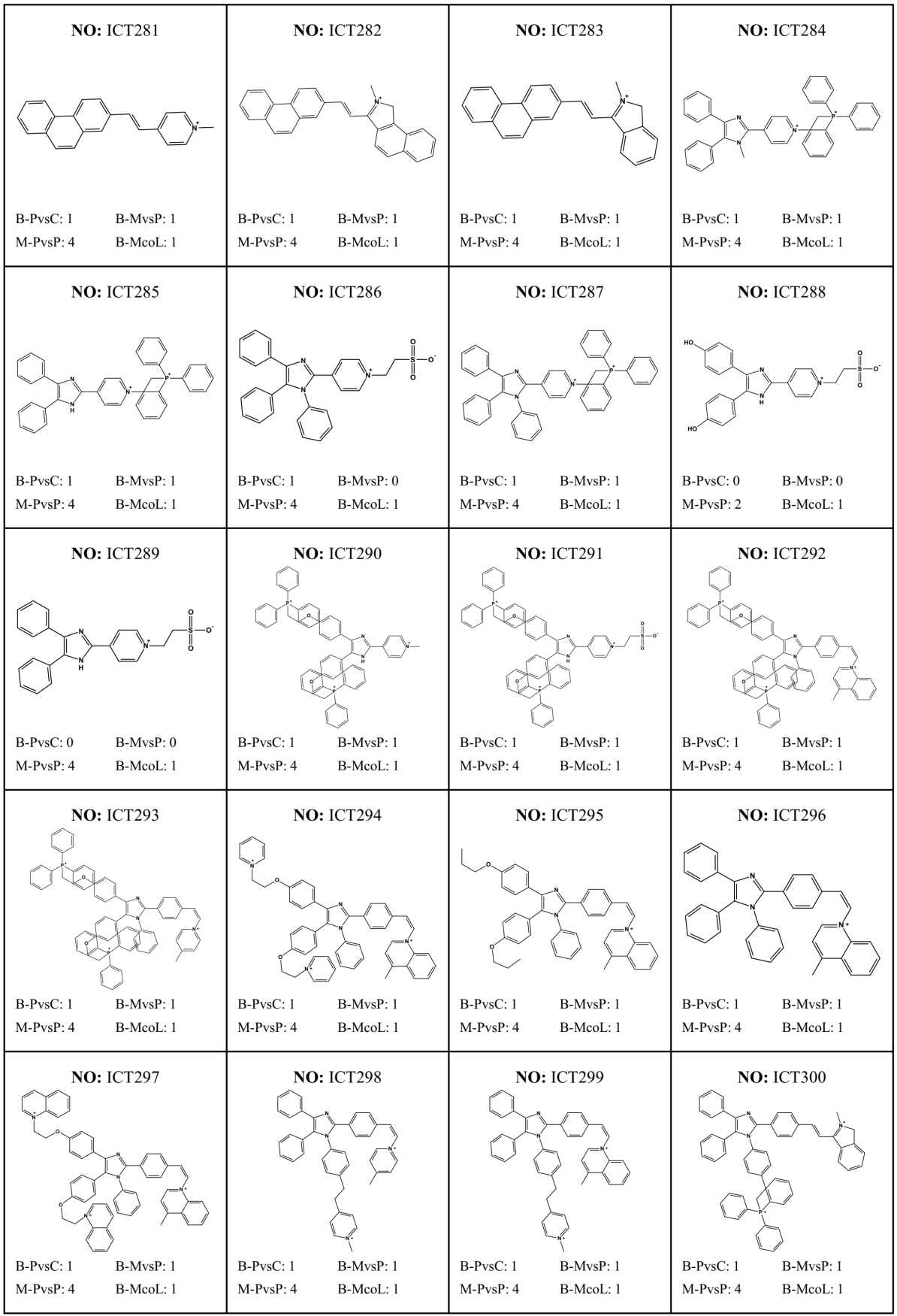


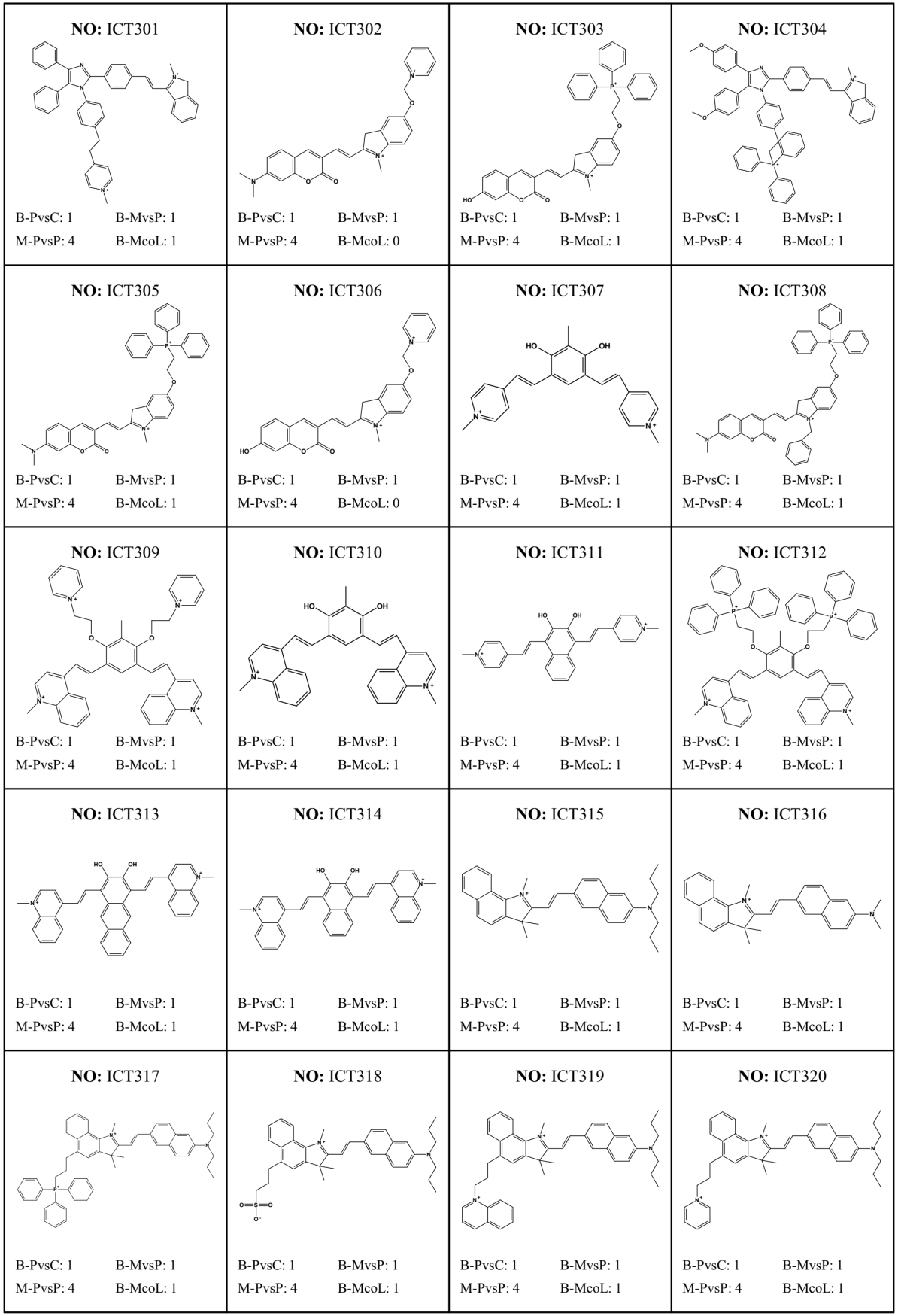


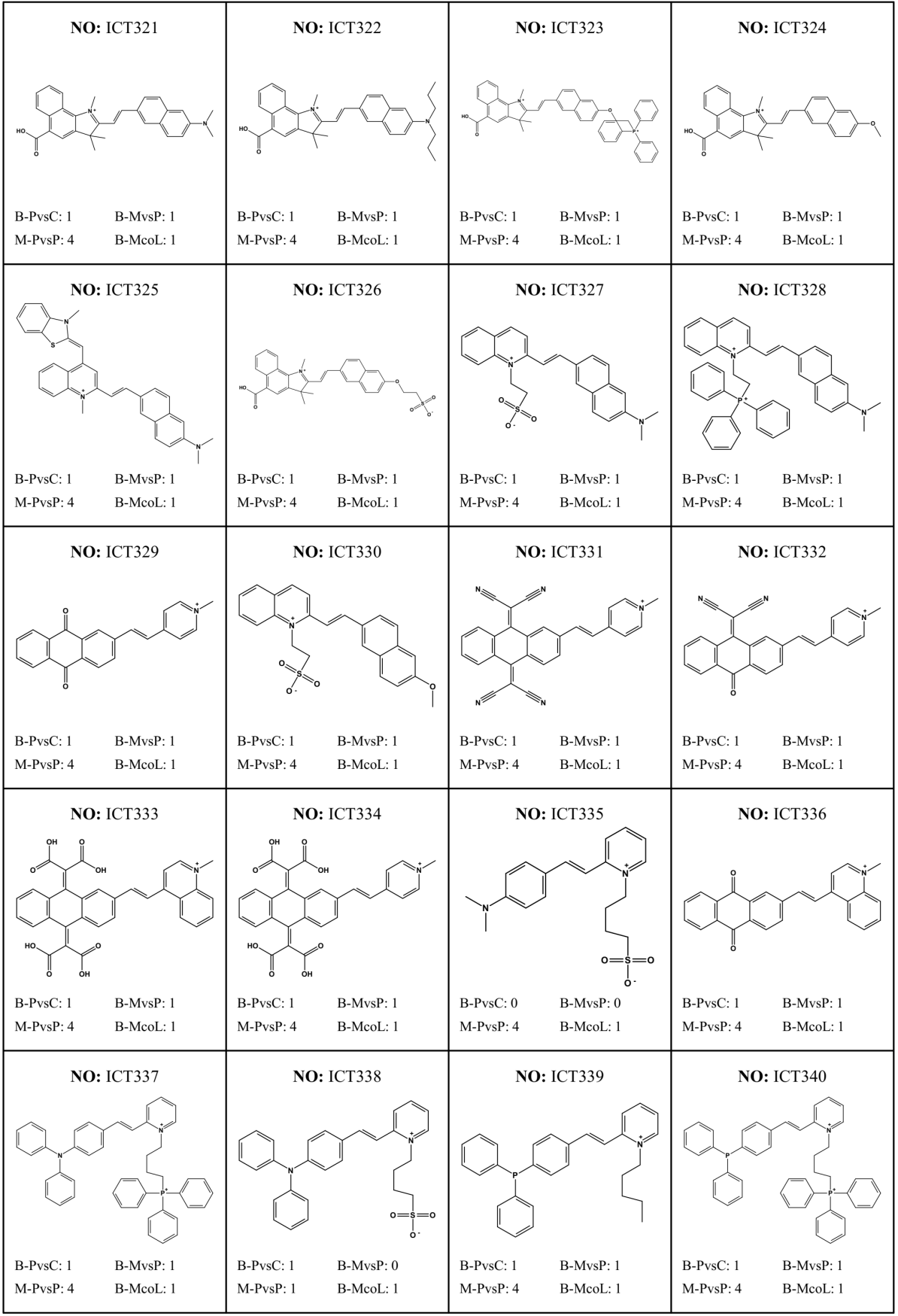


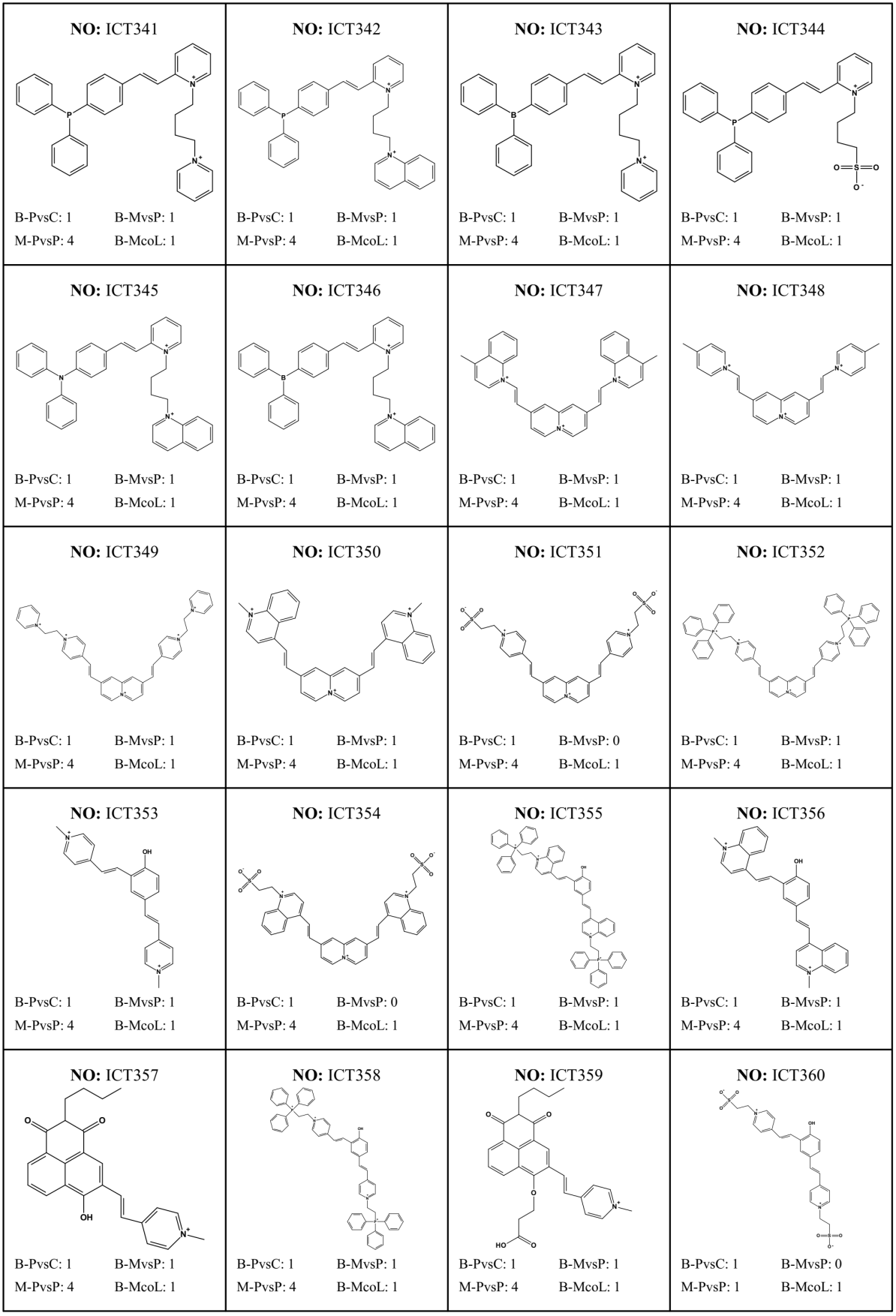


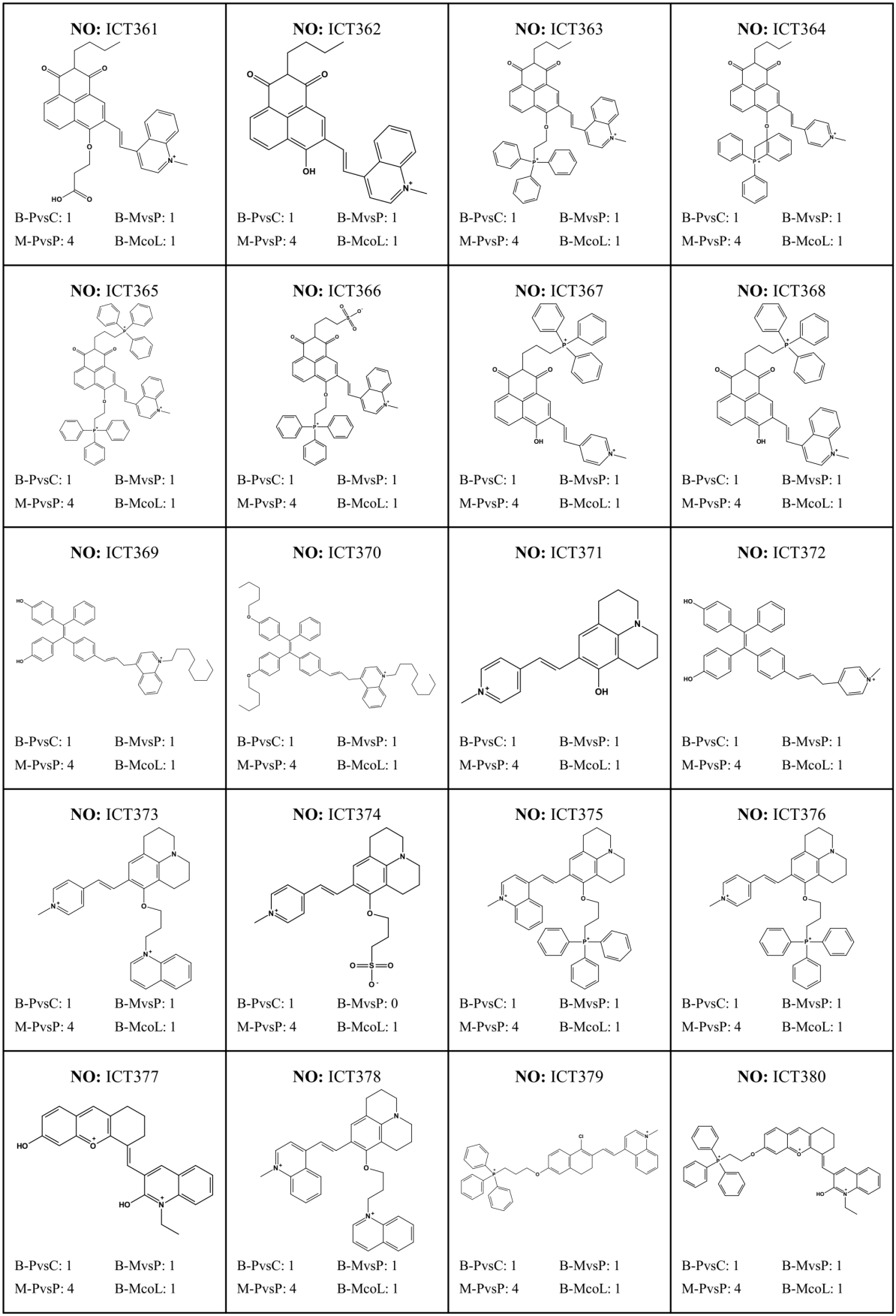


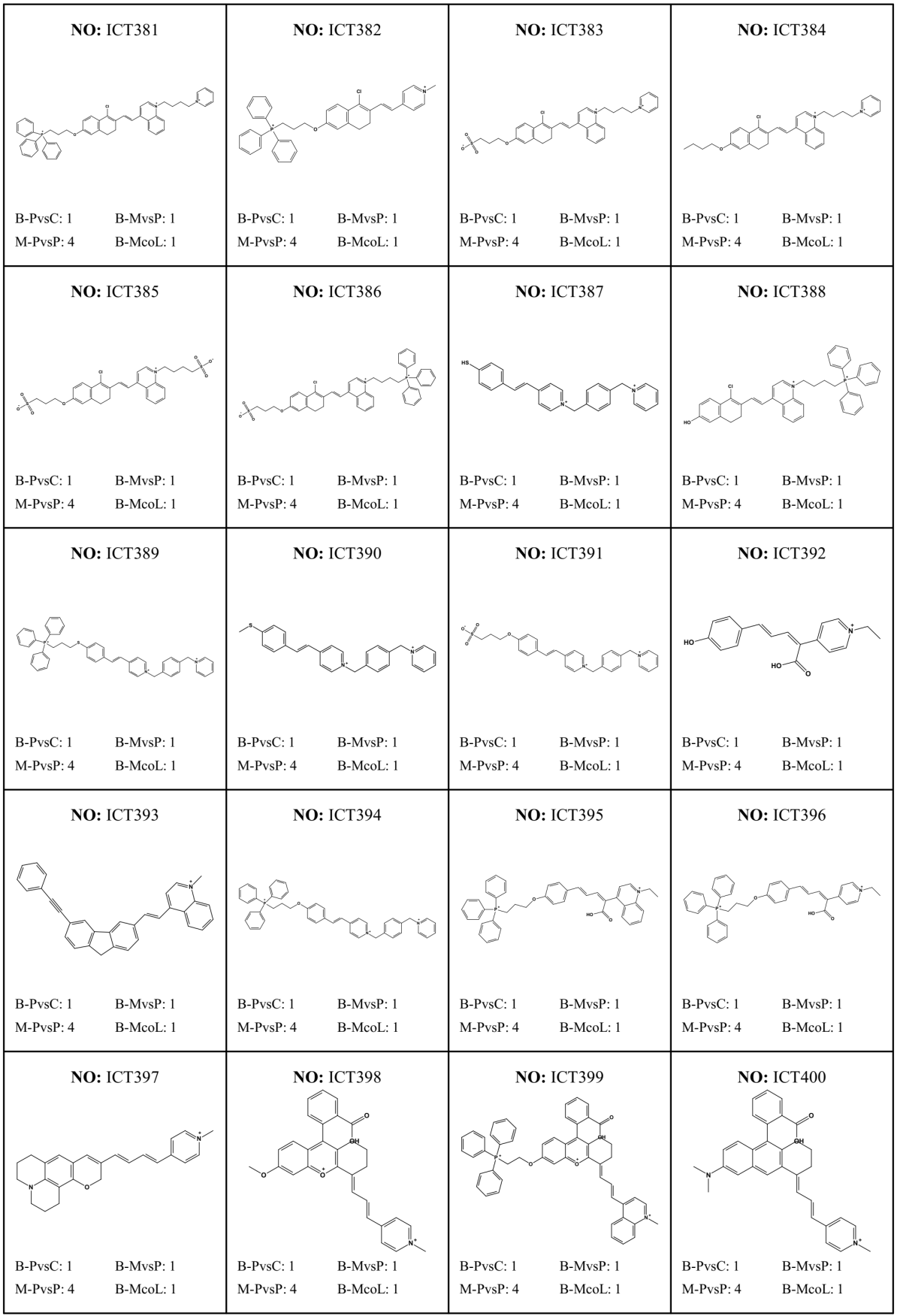


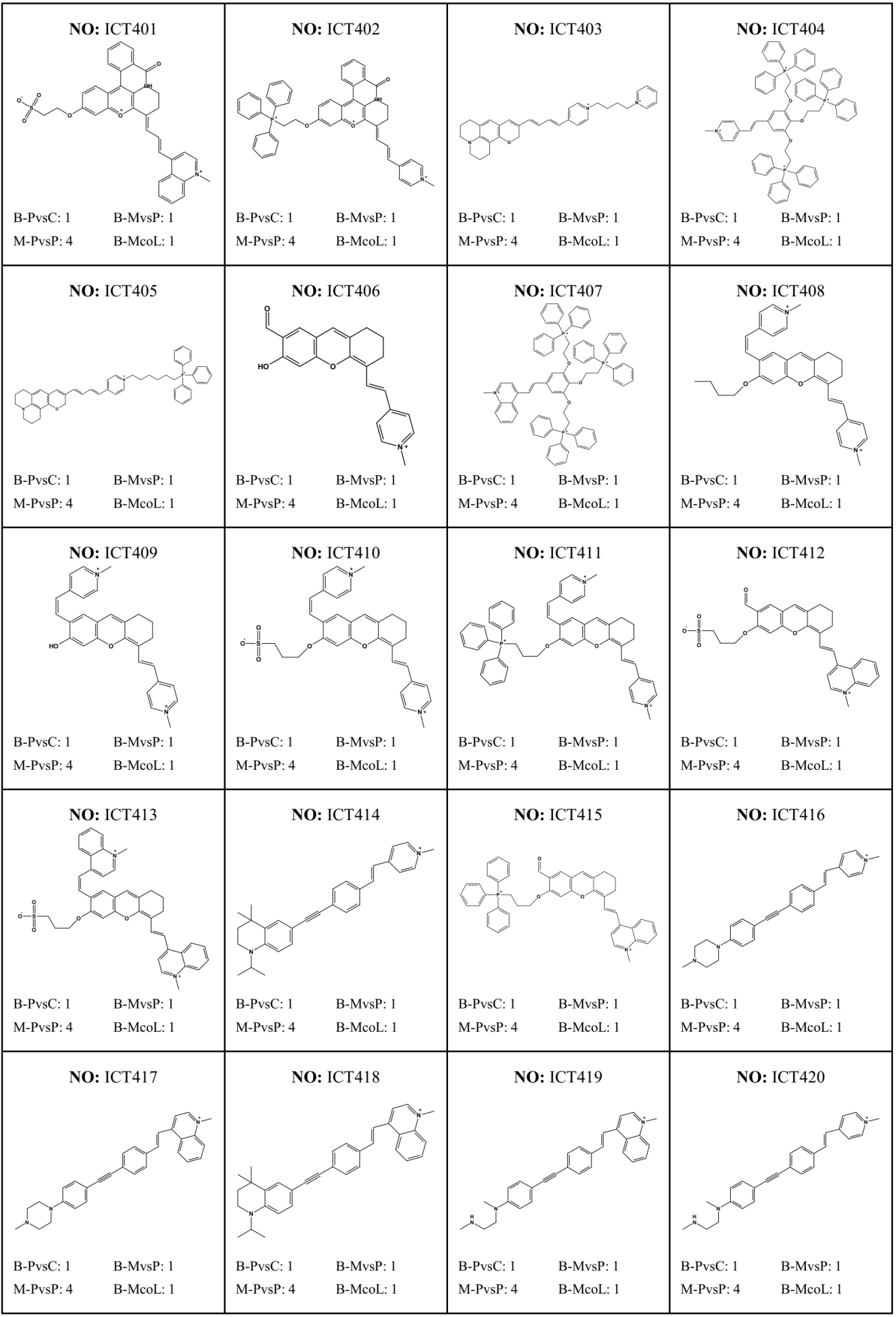


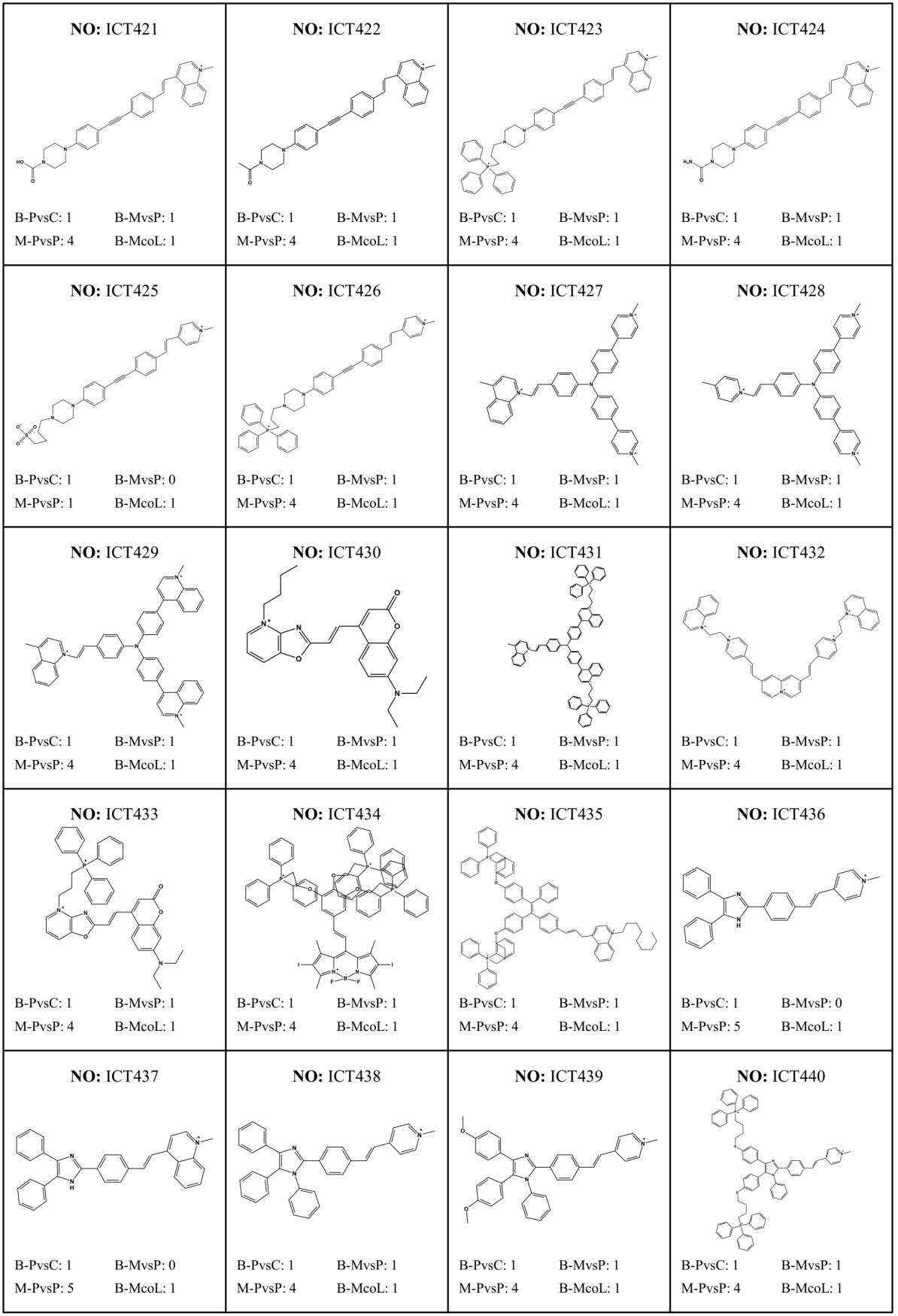


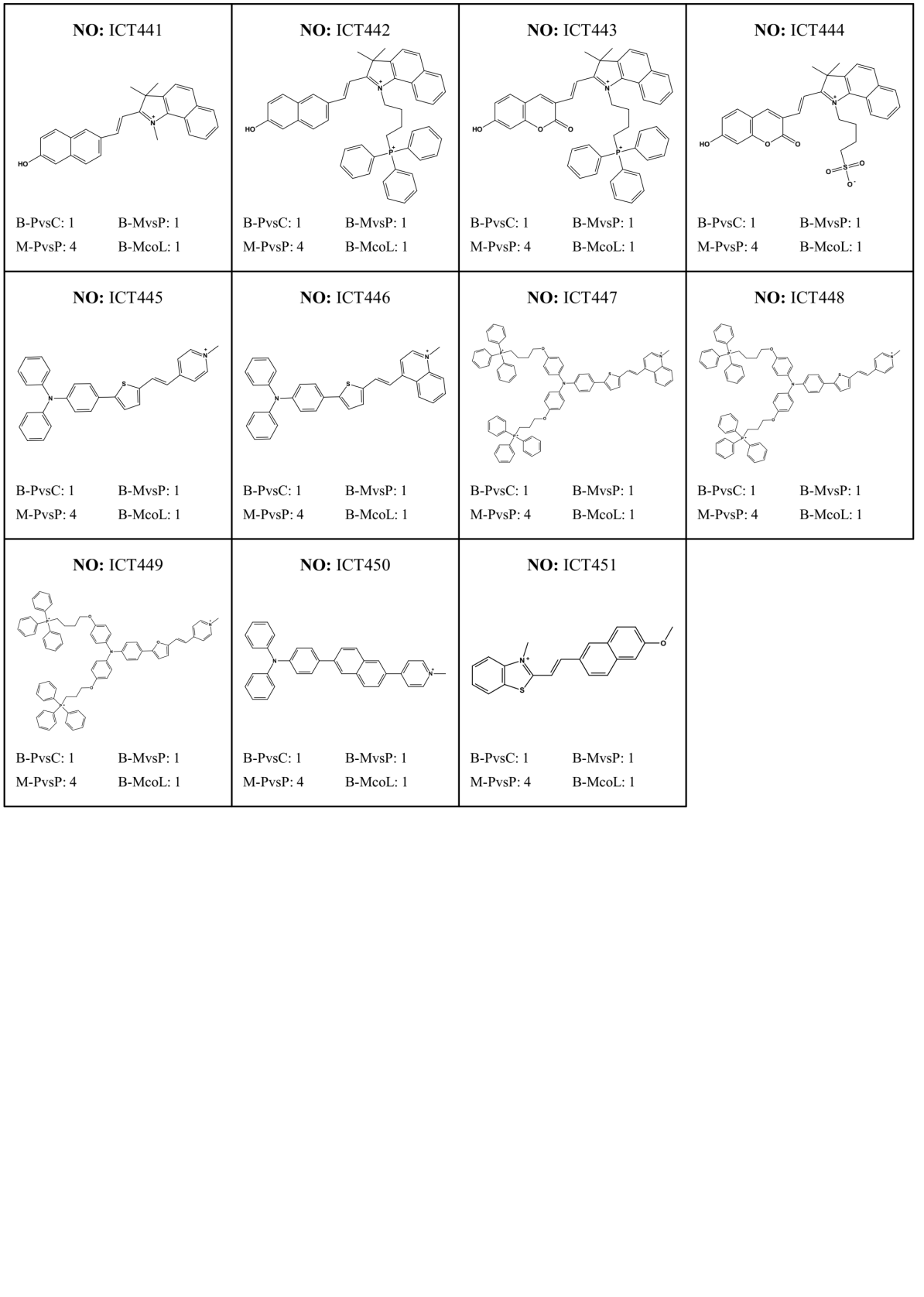


Table S10.

Molecular structure, identifier, and predicted labels by our machine learning framework for the designed 37 ROS-probes.


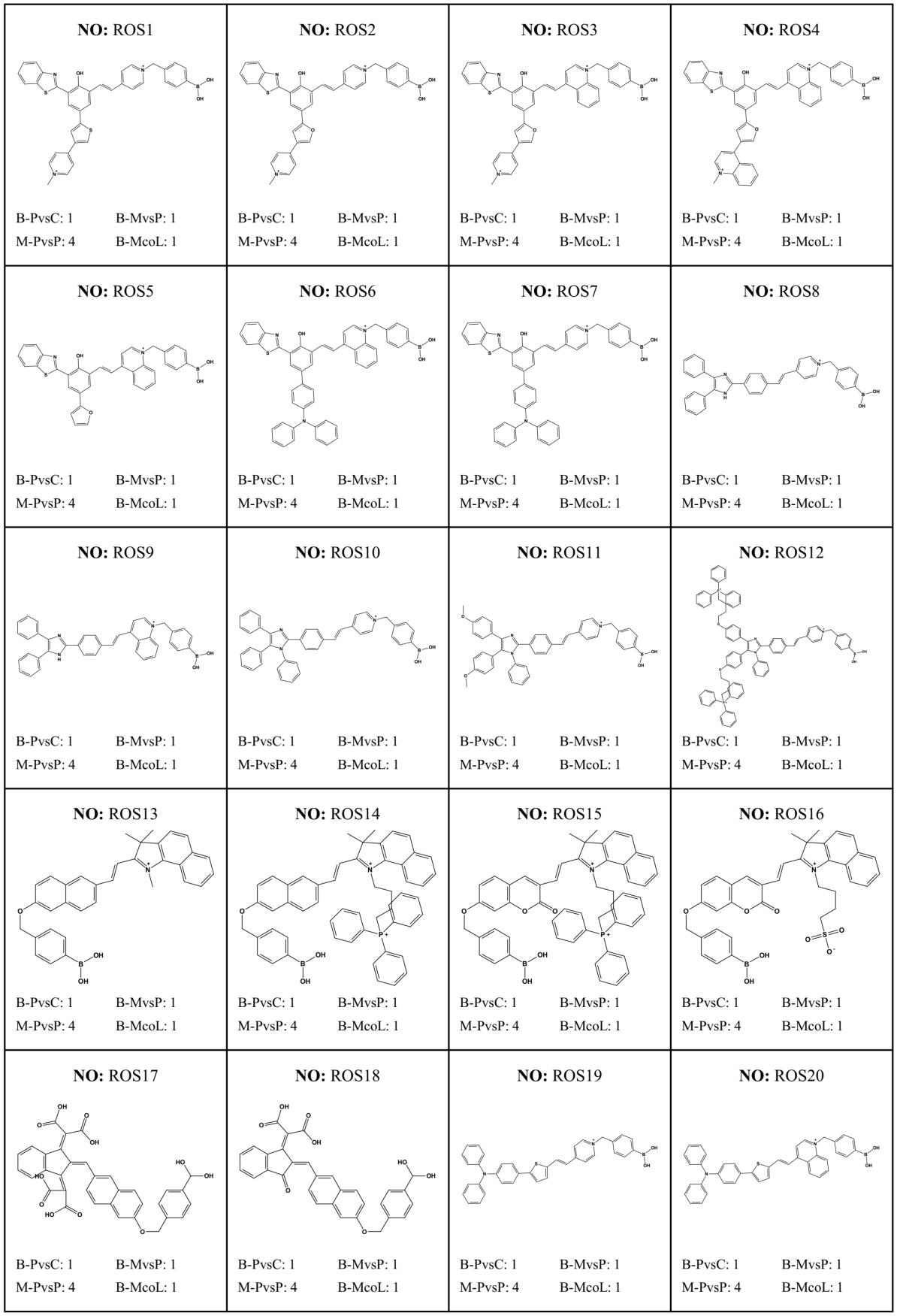


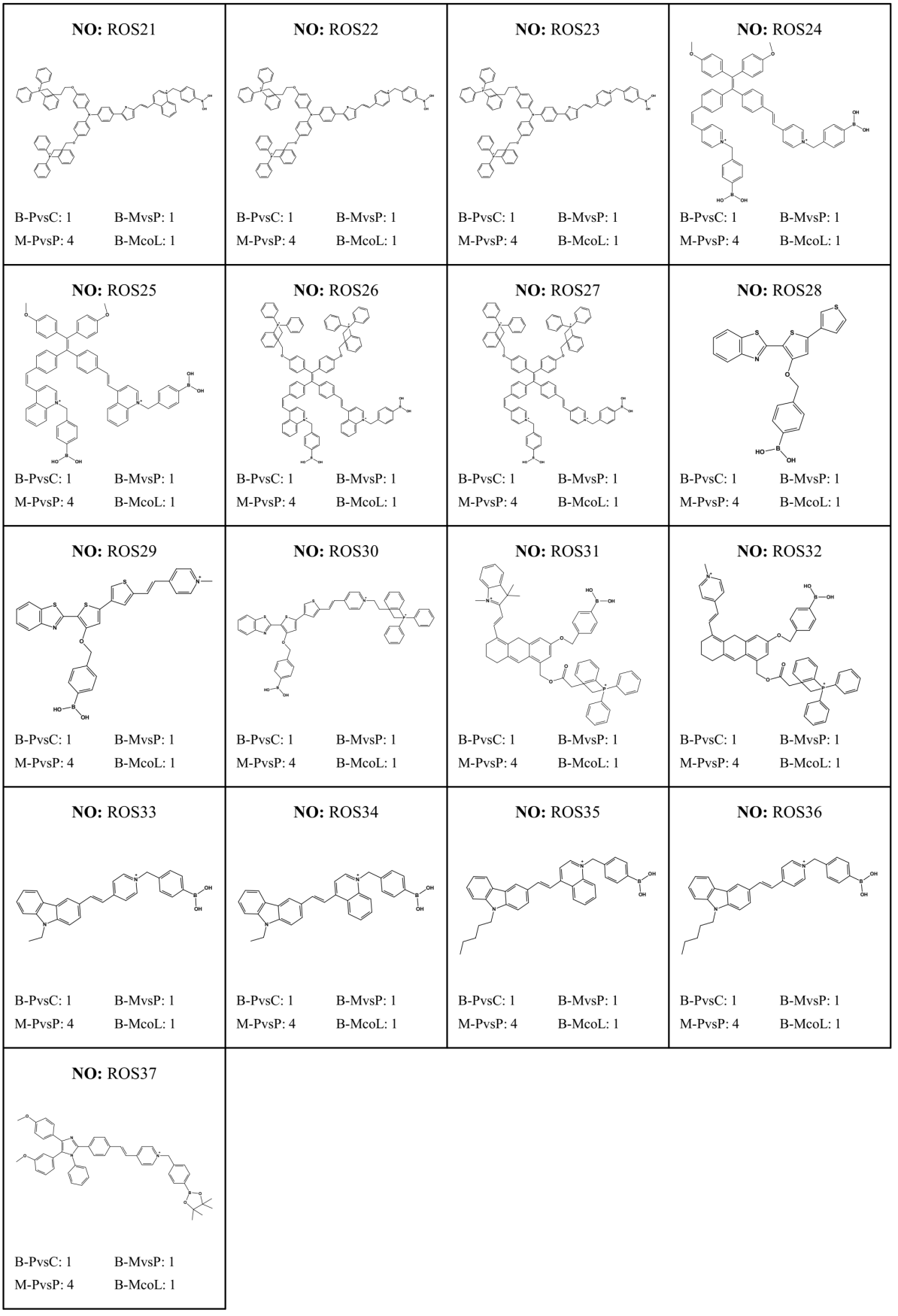


Table S11.

Molecular structure, identifier, and predicted labels by our machine learning framework for the designed 54 Viscosity-probes.


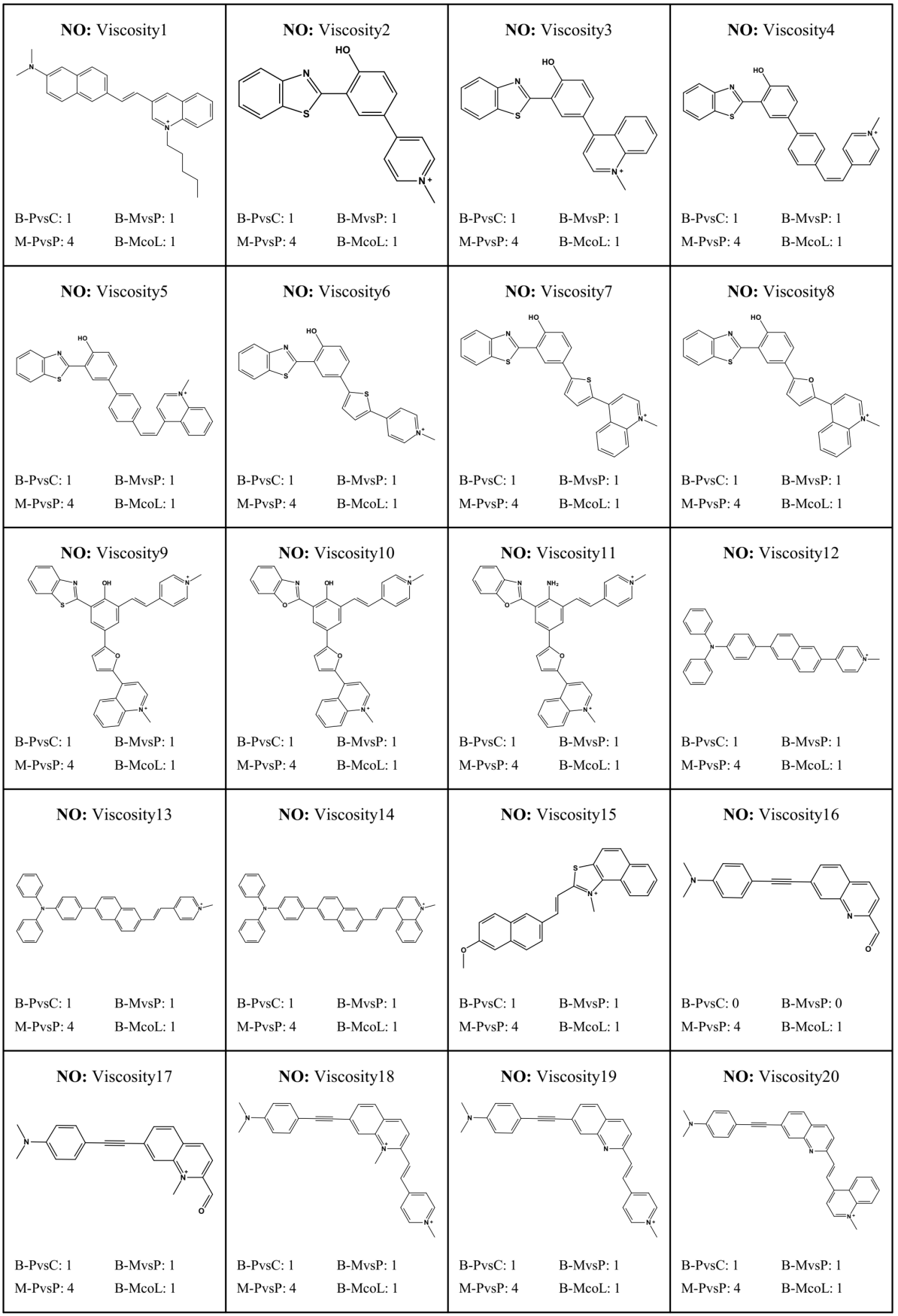


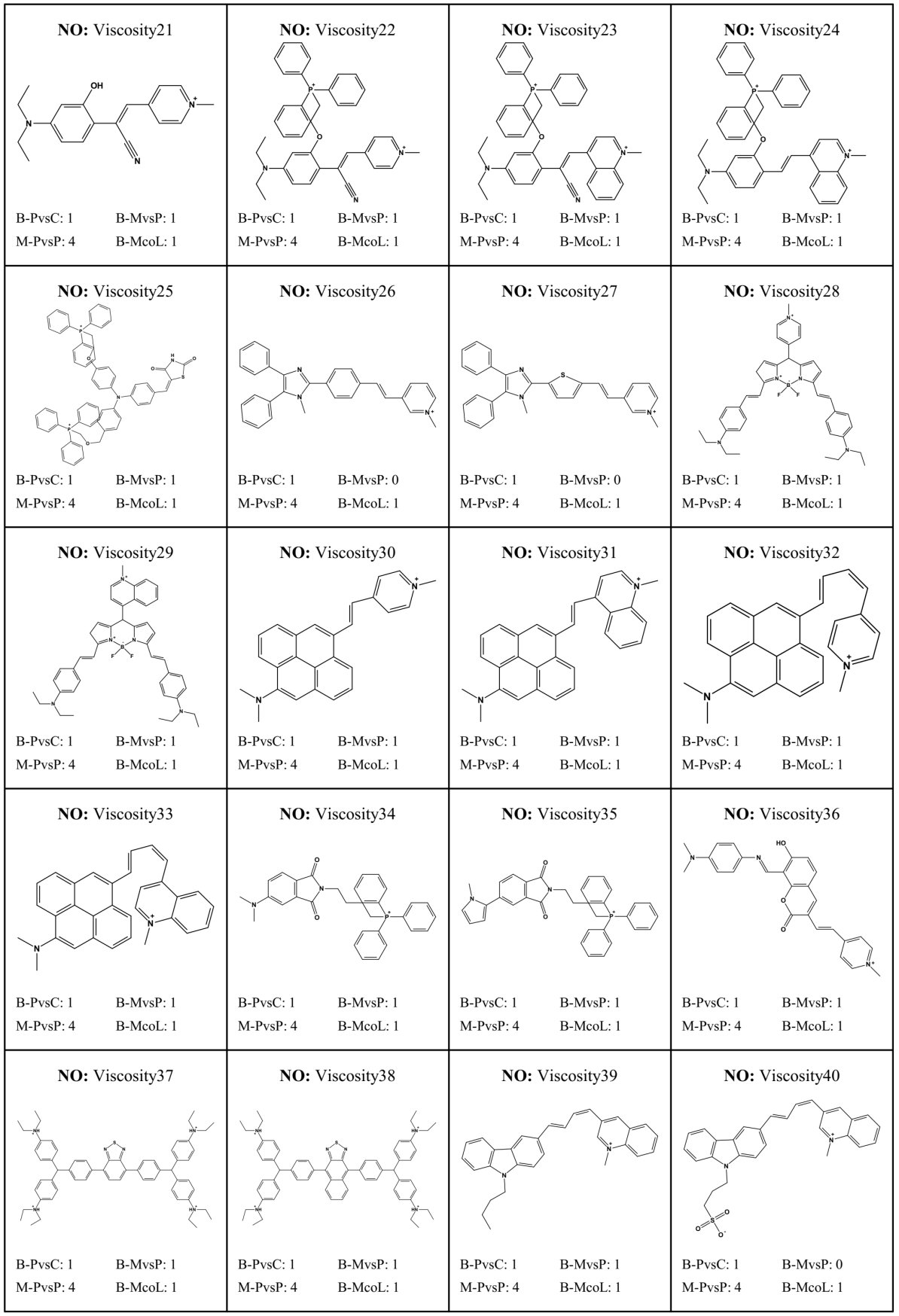


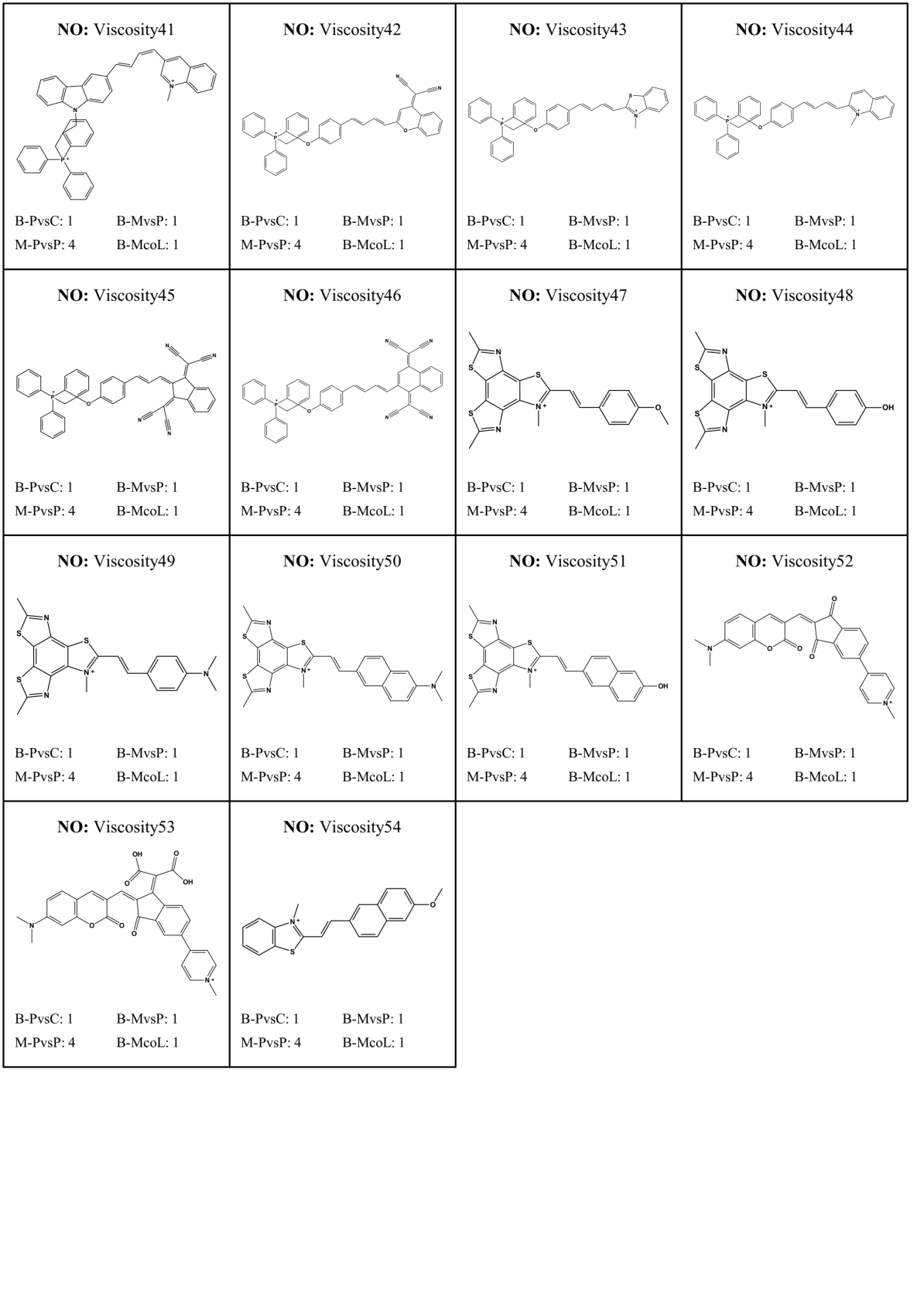


| Table S12. The performance of the models built by MolMapNet method. | | | | | | | | | |
| --- | --- | --- | --- | --- | --- | --- | --- | --- | --- |
| Model | MolMapNet Fmaps | Validation | | | | Test | | | |
|  |  | ACC | AUC | Recall | Precision | ACC | AUC | Recall | Precision |
| B-PvsC | MolDs+3FFs | 0.917 | 0.977 | 0.958 | 0.938 | 0.942 | 0.965 | 0.951 | 0.980 |
|  | MolDs+12FFs | 0.934 | 0.975 | 0.947 | 0.968 | 0.917 | 0.964 | 0.941 | 0.960 |
| B-MvsP | MolDs+3FFs | 0.826 | 0.892 | 0.865 | 0.847 | 0.826 | 0.894 | 0.827 | 0.880 |
|  | MolDs+12FFs | 0.820 | 0.908 | 0.885 | 0.825 | 0.832 | 0.895 | 0.857 | 0.866 |
| M-PvsP | MolDs+3FFs | 0.735 | 0.894 | 0.735 | 0.822 | 0.691 | 0.879 | 0.691 | 0.756 |
|  | MolDs+12FFs | 0.784 | 0.883 | 0.784 | 0.799 | 0.667 | 0.846 | 0.667 | 0.728 |
| B-McoL | MolDs+3FFs | 0.899 | 0.528 | 1.000 | 0.899 | 0.910 | 0.483 | 1.000 | 0.910 |
|  | MolDs+12FFs | 0.899 | 0.419 | 1.000 | 0.899 | 0.910 | 0.543 | 1.000 | 0.910 |

| Table S13. The coordinates of the optimized molecules | |
| --- | --- |
| Probe | Coordinates |
| P1-ALP S0 | C 6.63536900 -0.91327500 -0.34080300  C 6.18889400 -2.22617600 -0.11352500  C 4.84265400 -2.49945400 0.10491900  C 3.94817300 -1.42743700 0.09109500  C 4.38477600 -0.10155300 -0.13657900  C 5.74664300 0.15281500 -0.35454500  S 2.22588100 -1.43785400 0.32673600  C 2.20602500 0.30362000 0.10775600  N 3.37902400 0.83352600 -0.11871500  C 0.98631500 1.09241300 0.16967300  C -0.26555200 0.50276800 0.41371000  C -1.43518100 1.25117100 0.47792400  C -1.33697800 2.64126400 0.28660900  C -0.11781800 3.25307400 0.04531600  C 1.06516400 2.49948200 -0.02015000  O 2.21178800 3.12496800 -0.25202800  C -2.76903900 0.59035300 0.70719700  C -3.42410600 0.19465000 -0.61677500  N -4.73155200 -0.45609200 -0.41178700  C -4.78646100 -1.79036000 -0.23293400  C -5.99212000 -2.42126200 0.00996000  C -7.15729400 -1.65910900 0.06992100  C -7.07913300 -0.28040200 -0.11711000  C -5.84666700 0.29791100 -0.35744600  H 7.69893400 -0.72989900 -0.50926400  H 6.90899000 -3.04753400 -0.10791600  H 4.49853400 -3.52038200 0.28117500  H 6.08425600 1.17605000 -0.53029800  H -0.31832000 -0.58034100 0.56355500  H -2.23949100 3.25712200 0.33627900  H -0.04661800 4.33342400 -0.09555900  H 2.93465200 2.43398500 -0.26154700  H -3.44014800 1.27205000 1.25289200  H -2.64488400 -0.30846400 1.33092100  H -2.79250900 -0.50891300 -1.17445900  H -3.59182100 1.07191000 -1.25465300  H -3.84093900 -2.33014900 -0.29709100  H -6.00801100 -3.50293500 0.14542400  H -8.12063000 -2.13711300 0.25797800  H -7.96579700 0.35303900 -0.08322000  H -5.71886800 1.36908000 -0.51753700 |
| P1-ALP S1 | C 6.70697500 -0.67714000 -0.34055100  C 6.34382800 -2.02367200 -0.12369400  C 5.02435400 -2.39504700 0.09276300  C 4.06045000 -1.38400500 0.08870000  C 4.41461000 -0.02580600 -0.12893500  C 5.75813000 0.32778500 -0.34601400  S 2.35045300 -1.51767400 0.32500500  C 2.21362200 0.20516300 0.12475600  N 3.34967300 0.82401600 -0.10026600  C 0.97265800 0.94375400 0.18998000  C -0.25996200 0.35221500 0.42762400  C -1.44028500 1.10795100 0.47202100  C -1.36590300 2.52292300 0.28207000  C -0.17397200 3.14843800 0.04953500  C 1.02961800 2.38628200 -0.00712100  O 2.14210200 2.98923600 -0.22874000  C -2.76465500 0.45000800 0.66638300  C -3.48882600 0.19788800 -0.66835200  N -4.78648500 -0.39738000 -0.48703800  C -4.91451500 -1.77933600 -0.35396100  C -6.12169400 -2.34577800 -0.05512900  C -7.27658400 -1.54994700 0.14351100  C -7.12143900 -0.14754500 0.01275500  C -5.90828500 0.40530700 -0.28587400  H 7.75709000 -0.42875700 -0.50657300  H 7.11925000 -2.79260000 -0.12612300  H 4.75183900 -3.43823300 0.25930700  H 6.02499200 1.37257200 -0.51234800  H -0.31922500 -0.72759400 0.58262800  H -2.28388000 3.11225200 0.33030100  H -0.09964600 4.22648500 -0.09521600  H 2.90364600 2.26550300 -0.23092300  H -3.40246400 1.09232100 1.29496800  H -2.63595800 -0.50941400 1.18908500  H -2.87878200 -0.46304200 -1.30337200  H -3.60851000 1.14330600 -1.21954300  H -4.00760800 -2.35956400 -0.52064800  H -6.17240700 -3.43353000 0.02317400  H -8.23923000 -1.99517000 0.38635100  H -7.97213100 0.52361700 0.14585100  H -5.75211000 1.47766300 -0.39874500 |
| P2-VIS S0 | C -4.62415000 -1.08847000 -0.06269400  C -5.17269600 0.20148700 0.02282500  C -4.31374900 1.31978100 0.09025000  C -2.94668900 1.15279400 0.07194500  C -2.37237000 -0.13847700 -0.01400900  C -3.24560800 -1.24217300 -0.07983400  O -6.47834800 0.46490500 0.04635900  C -7.40763700 -0.59691700 -0.01838000  C -0.95448500 -0.37849100 -0.03729700  C 0.04330200 0.55113600 0.00833800  C 1.42530700 0.20602500 -0.00820200  N 2.43662200 1.08811800 -0.04044300  C 3.70471400 0.51180000 -0.01184000  C 3.64779100 -0.88974000 0.01429400  S 1.99438300 -1.42626700 0.03049700  C 4.93668700 1.17212100 -0.00617800  C 6.08996600 0.39724000 0.01449500  C 6.02836700 -1.00387700 0.03116300  C 4.80573500 -1.66578900 0.03286300  C 2.26390700 2.53227000 -0.08730000  H -5.26361000 -1.96935800 -0.11558700  H -4.76194000 2.31285000 0.15686000  H -2.31045800 2.03847600 0.12575400  H -2.82609000 -2.24936700 -0.14626400  H -8.40267900 -0.13841600 0.01336100  H -7.29631800 -1.27971900 0.83907600  H -7.30103000 -1.16616000 -0.95564900  H -0.67294300 -1.43646700 -0.10210300  H -0.19809500 1.61226200 0.07010500  H 4.99987600 2.26044400 -0.01087400  H 7.06237500 0.89356400 0.01986200  H 6.95217300 -1.58550900 0.04637200  H 4.75263700 -2.75560700 0.05012900  H 3.05012900 2.96247200 -0.71751400  H 2.32706300 2.95760100 0.92419600  H 1.29885800 2.78281800 -0.53607800 |
| P2-VIS S1 | C 4.64770300 -1.10128900 0.00004700  C 5.15431300 0.22147400 -0.00003900  C 4.25347300 1.31704400 -0.00006300  C 2.90179600 1.10805300 -0.00001900  C 2.35517400 -0.22019000 0.00006000  C 3.29103400 -1.30595800 0.00009200  O 6.44008100 0.52493600 -0.00008200  C 7.42937100 -0.49391800 -0.00009700  C 0.97337400 -0.49910500 0.00010800  C -0.05070500 0.47479700 0.00011900  C -1.40664900 0.17698000 0.00008400  N -2.43182000 1.10056800 0.00011600  C -3.68830700 0.54748600 0.00002800  C -3.66031500 -0.86853000 -0.00001900  S -2.02840600 -1.46013300 0.00000800  C -4.92407100 1.21490700 -0.00001100  C -6.08395600 0.45892100 -0.00007700  C -6.04746400 -0.94620700 -0.00011100  C -4.83448700 -1.61952800 -0.00008600  C -2.16001300 2.52658900 -0.00000300  H 5.32324000 -1.95530200 0.00009200  H 4.67385000 2.32374800 -0.00011800  H 2.23692800 1.97178700 -0.00004500  H 2.90370000 -2.32693500 0.00016400  H 8.39633400 0.01982100 -0.00015000  H 7.34595700 -1.12149200 -0.90004600  H 7.34604100 -1.12145600 0.89988400  H 0.68822100 -1.55405500 0.00015400  H 0.22284500 1.52967100 0.00014300  H -4.97876500 2.30252100 -0.00001200  H -7.04850000 0.96971100 -0.00010600  H -6.98018200 -1.51230900 -0.00016100  H -4.79616400 -2.70972000 -0.00012000  H -3.10151400 3.08084100 0.00040700  H -1.58884800 2.80634700 -0.89687100  H -1.58817300 2.80634200 0.89643200 |
| P3-ROS S0 | C -8.84752200 4.63977300 0.81141600  C -7.71725200 4.71928100 -0.01168200  C -6.93749900 3.58426800 -0.22812900  C -7.25161100 2.34889700 0.35299200  C -8.38264700 2.29070200 1.18792700  C -9.16717900 3.41218900 1.41192300  O -9.66888700 5.67298400 1.08373900  C -9.39364900 6.92942400 0.51540400  C -6.37931100 1.19079300 0.10576700  C -6.65694100 -0.17137000 0.19976600  N -5.46810500 -0.80704600 -0.11489600  C -4.54353500 0.17093600 -0.39664800  N -5.07975300 1.36725200 -0.26341400  C -7.89446700 -0.90040000 0.51828500  C -3.15222500 -0.03236000 -0.80822700  C -7.97103300 -1.79159400 1.59517200  C -9.14930600 -2.47513500 1.89350200  C -10.29040000 -2.27142200 1.10696500  C -10.22674500 -1.37878600 0.02464200  C -9.04791300 -0.70946700 -0.26354200  C -2.68195000 -1.18078400 -1.46656400  C -1.35227300 -1.27267000 -1.85633200  C -0.43902500 -0.23148600 -1.61039000  C -0.92029500 0.92478600 -0.96185400  C -2.24418400 1.02110500 -0.57489100  O -11.47283900 -2.87842300 1.31086600  C -11.60079200 -3.78716700 2.37799000  C 0.93896600 -0.39538100 -2.03774800  C 1.95043300 0.49254700 -1.87385900  C 3.31412000 0.29132700 -2.31224600  C 3.76973700 -0.85654400 -2.99580800  C 5.08712300 -0.96549600 -3.37377100  N 5.98008300 0.01154400 -3.10522800  C 5.58396700 1.12683600 -2.45443000  C 4.27924100 1.29048100 -2.05580700  C -5.26363500 -2.21761000 -0.07722600  C 7.38973800 -0.12027900 -3.52576800  C 8.34975500 -0.06280900 -2.36574900  C 9.33115600 0.93172300 -2.31513300  C 10.23264900 0.97596100 -1.25274600  C 10.17227900 0.03747400 -0.21310800  C 9.17976900 -0.95518400 -0.27461700  C 8.28231100 -1.01050700 -1.33554800  C -5.89089000 -3.03422300 -1.01985000  C -5.68940600 -4.41252800 -0.97557100  C -4.86261600 -4.97033800 0.00045400  C -4.24103800 -4.14793600 0.94071800  C -4.44466600 -2.76967200 0.90943500  B 11.17928400 0.09365500 0.97805500  O 11.20500100 -0.82928700 1.98292000  C 12.09742100 -0.33000100 3.00514400  C 13.00115600 0.67014900 2.19475400  O 12.12156400 1.07061200 1.11917300  C 14.20759000 -0.00280900 1.55013900  C 13.43396300 1.90550400 2.96162400  C 12.83620800 -1.50026600 3.62664000  C 11.22865700 0.36247400 4.04912800  H -7.43582000 5.65786800 -0.49049900  H -6.05467300 3.65254100 -0.86712900  H -8.64651400 1.35482000 1.68373200  H -10.04131600 3.36394800 2.06509500  H -10.17786600 7.61150000 0.86649900  H -9.41872500 6.89360700 -0.58706400  H -8.41224000 7.31747700 0.83733000  H -7.09469100 -1.95408000 2.22677800  H -9.16717600 -3.15764600 2.74357100  H -11.12167500 -1.23205300 -0.58377000  H -9.01287100 -0.02337200 -1.11324800  H -3.35740300 -2.00361700 -1.69947300  H -1.00896200 -2.17210400 -2.37398200  H -0.24933800 1.76070900 -0.75411000  H -2.60898000 1.92065200 -0.07676800  H -12.63373000 -4.15522700 2.35325200  H -11.41718900 -3.30124200 3.35102800  H -10.91307400 -4.64265800 2.26896600  H 1.14743200 -1.34673200 -2.53778400  H 1.76100300 1.44489000 -1.37205600  H 3.10143900 -1.68081700 -3.24350700  H 5.46727000 -1.83869400 -3.90458200  H 6.35228100 1.87751800 -2.26445300  H 4.00279300 2.20835900 -1.53520600  H 7.47072000 -1.07538100 -4.06111400  H 7.59966600 0.68219100 -4.24616900  H 9.39298700 1.67430400 -3.11549600  H 10.99681600 1.75724500 -1.22896600  H 9.11500400 -1.69950000 0.52331000  H 7.52368400 -1.79791200 -1.36232300  H -6.52818900 -2.58552000 -1.78431700  H -6.17881500 -5.05346000 -1.71243300  H -4.70391200 -6.05078800 0.02968000  H -3.59733200 -4.58133700 1.70954300  H -3.97067200 -2.11671900 1.64541900  H 14.96374200 -0.27894700 2.29853000  H 14.66806100 0.69771700 0.83829900  H 13.91577600 -0.90807900 0.99740900  H 14.05001800 2.54563300 2.31311900  H 14.03987200 1.62720100 3.83662400  H 12.57344100 2.49470200 3.30377100  H 13.34562900 -2.10922200 2.86873800  H 12.12264500 -2.14528400 4.16016500  H 13.58229100 -1.14681300 4.35370600  H 11.82628700 0.71832700 4.90025200  H 10.48545700 -0.35492100 4.42661000  H 10.68875900 1.21886100 3.61868100 |
| P3-ROS S1 | C -8.30990300 4.95953900 -0.09765200  C -7.22964600 4.73671000 -0.96004100  C -6.57107800 3.51313200 -0.93448300  C -6.96825000 2.48196000 -0.07382200  C -8.04796200 2.72664500 0.79498300  C -8.70582600 3.94229300 0.78454200  O -9.01306400 6.10368900 -0.04236400  C -8.66529300 7.17412200 -0.89370400  C -6.20841700 1.22803200 -0.06816400  C -6.64200900 -0.08534700 0.28145100  N -5.53031100 -0.86958300 0.17925300  C -4.47800700 -0.06071900 -0.25853900  N -4.91422000 1.20053200 -0.38574300  C -7.97623900 -0.57230900 0.61724100  C -3.13879000 -0.43707200 -0.56283500  C -8.22063700 -1.42741600 1.70129000  C -9.50305900 -1.87193600 1.99825300  C -10.58092300 -1.47664900 1.19634600  C -10.34964800 -0.62441600 0.10391000  C -9.07445000 -0.17680600 -0.17449000  C -2.62352600 -1.77128500 -0.60026600  C -1.31873700 -2.01146200 -0.92562000  C -0.39749200 -0.95763700 -1.23923700  C -0.92441300 0.37812100 -1.20921100  C -2.22830200 0.62003600 -0.89295300  O -11.85074100 -1.85721500 1.39485300  C -12.16153800 -2.71747400 2.47152200  C 0.93635900 -1.27031900 -1.55685500  C 1.94254600 -0.35190800 -1.87934800  C 3.27459200 -0.66685600 -2.20225100  C 3.80458200 -2.00013900 -2.25382100  C 5.10864700 -2.22416800 -2.57934700  N 5.96735200 -1.20465300 -2.86831700  C 5.50735800 0.08375900 -2.82933700  C 4.21520700 0.36979100 -2.51339900  C -5.50059300 -2.28169000 0.43158000  C 7.36965700 -1.46266600 -3.20737700  C 8.33865000 -0.96791700 -2.15633200  C 9.39541300 -0.12894800 -2.51292200  C 10.30480100 0.30643500 -1.55195100  C 10.17931800 -0.07662100 -0.21153600  C 9.10975700 -0.91594100 0.13550800  C 8.20297100 -1.35798300 -0.81963600  C -6.01008400 -3.15863800 -0.52222400  C -5.97835200 -4.52810400 -0.27301900  C -5.44045100 -5.01064800 0.91843500  C -4.93669200 -4.12351800 1.86859100  C -4.96897300 -2.75304500 1.63007000  B 11.19460200 0.41494200 0.86327200  O 11.16236700 0.01827100 2.16708600  C 12.10379100 0.83355200 2.90462600  C 13.06470200 1.34062600 1.76584600  O 12.20894500 1.28664000 0.59976700  C 14.23349200 0.39679400 1.50014200  C 13.56756400 2.76361500 1.93771500  C 12.76704400 -0.02546000 3.96739900  C 11.30050100 1.95418000 3.55760700  H -6.89618900 5.50727200 -1.65357200  H -5.72678700 3.34337200 -1.60377500  H -8.36232500 1.96310600 1.50637600  H -9.53652900 4.13902100 1.46400000  H -9.36589000 7.98831100 -0.67477400  H -8.76028200 6.89355000 -1.95511300  H -7.63743900 7.52340500 -0.70451700  H -7.40066000 -1.73767600 2.34791700  H -9.65208300 -2.52205100 2.85882100  H -11.19808600 -0.33178300 -0.51621000  H -8.91364800 0.47782100 -1.03203500  H -3.26706500 -2.61945000 -0.38829100  H -0.95694600 -3.04147300 -0.95449900  H -0.27473700 1.22191300 -1.44215300  H -2.60893800 1.64008900 -0.87596400  H -13.24293700 -2.89069800 2.42970000  H -11.90712800 -2.25802700 3.43991600  H -11.63761800 -3.68218200 2.37973500  H 1.18844100 -2.33334100 -1.54396700  H 1.69456300 0.71191000 -1.88682200  H 3.17931000 -2.86504500 -2.03746900  H 5.52677700 -3.22990300 -2.62671600  H 6.23655800 0.85933000 -3.06431400  H 3.89851700 1.41356600 -2.50124700  H 7.47313000 -2.54701000 -3.34520200  H 7.58837000 -0.99076400 -4.17558100  H 9.50913900 0.18607800 -3.55315300  H 11.12742400 0.96118900 -1.84868200  H 8.99011300 -1.22848900 1.17542400  H 7.37951600 -2.01217700 -0.52250400  H -6.42192500 -2.76807100 -1.45396500  H -6.37422300 -5.22147400 -1.01718000  H -5.41486700 -6.08524400 1.10882600  H -4.51739900 -4.49963500 2.80343400  H -4.57812900 -2.04678200 2.36456000  H 14.96909900 0.42655000 2.31545200  H 14.73473000 0.70502000 0.57193400  H 13.89255100 -0.64058100 1.37545000  H 14.21905700 3.02767900 1.09261900  H 14.15493600 2.85971100 2.86224500  H 12.74218000 3.48503100 1.96952800  H 13.21552700 -0.92910000 3.53729100  H 12.01779200 -0.33634100 4.70924300  H 13.55016100 0.54053000 4.49219200  H 11.93446600 2.58717700 4.19329200  H 10.51678700 1.50924700 4.18659300  H 10.81390000 2.59108400 2.80560600 |
